# Scanning Electrochemical Cell Microscopy for Sub-Micrometer Mass Spectrometric Studies of Electrochemical Reactions

**DOI:** 10.1021/acselectrochem.5c00095

**Published:** 2025-04-21

**Authors:** Lingjie Zhang, Madison E. Edwards, Oluwasegun J. Wahab, Hugo Y. Samayoa-Oviedo, Dallas P. Freitas, Xin Yan, Lane A. Baker

**Affiliations:** Department of Chemistry, 14736 Texas A&M University, College Station, Texas 77843, United States

**Keywords:** scanning electrochemical cell microscopy, single entity electrochemistry, scanning probe, mass spectrometry, nanoelectrospray ionization

## Abstract

Nanoscale electrochemistry has been significantly advanced through the utilization of nanopipettes, enabling precise electrode area confinement and localized measurements. In particular, scanning electrochemical cell microscopy (SECCM) has leveraged the use of nanopipettes to facilitate measurement of electrochemical processes with high spatiotemporal resolution. While nano electrochemistry is well-suited to study processes at the sub-micrometer level, there is a need for complementary analytical techniques that can enable the detection of intermediates and help to elucidate reaction pathways that occur in the small volumes. In this work, we demonstrate the coupling of SECCM with MS for the detection of reaction products formed by the oxidation of uric acid. Specifically, species generated at the tip of an SECCM probe could be delivered to a mass spectrometer via nanoelectrospray ionization and exhibit both stable ion signal and high sensitivity. We demonstrate that this workflow enables the detection of analytes generated from SECCM probes of 3 μm and 900 nm tip diameter, despite the low conversion ratio associated with the smaller nanopipette diameters. Results presented herein demonstrate the SECCM-MS workflow as a powerful approach to detect low-abundance species formed from micro- and nanoscale electrochemical reactions.

## Graphical abstract

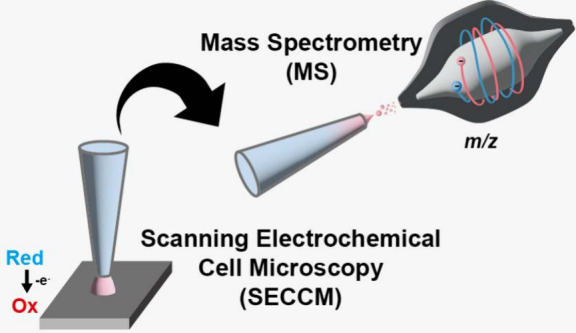

## Introduction

Scanning electrochemical cell microscopy (SECCM) is a nanoscale electrochemical technique which has proven adept at collecting high-resolution information related to the structure and activity of electrochemical interfaces.^1–3^ In a typical SECCM setup, a solution-filled nanopipette is scanned over regions of interest in a substrate.^2,4^ The substrate acts as the working electrode (WE) and a quasireference counter electrode (QRCE) is housed in the nanopipette. When the nanopipette and the substrate are brought in close proximity, an electrochemical cell is formed between the nanopipette and the substrate, where local electrochemical information related to the substrate in contact with this electrochemical cell can be measured. Applications of SECCM have grown rapidly^6–8^ and include the studies of passivation of electrodes used for Li-ion batteries^9^ and the measurement of heterogenous electron transfer rates in 2D carbon nanotube networks.^10^

SECCM is an especially compelling tool for the determination of nanoscale electrocatalytic reactivity. For instance, analyzing how different gold facets affect the competition between CO_2_ electroreduction and H_2_ evolution,^11^ enhancing mass transfer in hydrogen evolution reaction on Pt surfaces,^12^ mapping photoelectrochemical behavior of WSe_2_ nanosheets,^13^ and measuring electrocatalytic activity at individual nanoparticles.^14,15^ In electrocatalytic systems, SECCM can both provide information related to interfacial structure/activity and operate in regimes that are not mass transfer limited. A major challenge in electrochemical measurements is the inherent disconnect between the electron transfer event and the explicit chemical transformation, a disconnect that becomes even more difficult to address for complex reactions. As reactions become more complicated, the formation of intermediates and different branches of reaction pathways require advanced approaches to determine the chemical nature of the electron transfer event measured. Electrochemical tools, such as rotating ring disk electrodes,^16^ other generator-collector approaches,^17–20^ and hybrid SECCM-SECM^21–23^ for probing electrochemical reactions beyond electron transfer, have proven especially powerful for revealing chemical species. Complementary approaches, such as spectroelectrochemical techniques^24,25^ and SECCM-OM,^26–30^ have also proven informative routes to study electrochemical reactions. Mass spectrometry (MS) provides one of the most compelling approaches to understand the chemical consequences of electron transfer due to an inherently high specificity for chemical analysis.^31–38^ Towards this goal, differential electrochemical mass spectrometry (DEMS) has become a routine approach for electrocatalytic studies^39–41^ especially when gaseous species are involved. Flow-cells have also found application for integration of electrochemistry with mass spectrometry (EC-MS).^42–44^

Here, we combine SECCM with MS at the simplest level by utilizing the same nanopipette for both SECCM analysis and sample introduction to the mass spectrometer via nano-electrospray ionization (nESI). Studies reported here are rooted in nESI,^45–47^ (online methods), or nanodesorption electrospray ionization (for offline products generated on an electrode surface)^48,49^ that have been previously reported for EC-MS analyses. Here, we utilize a background-subtracted MS measurement to monitor the electrochemical oxidation of uric acid (UA) in the tip of a SECCM probe. SECCM feedback is utilized for control of the nanopipette at the electrode interface, and the inherent limits of detection for the configuration studied are considered. SECCM-MS takes advantage of the local concentration of species generated at the tip of the SECCM probe to enrich product concentrations at the tip of the probe, which allows for the detection of reaction products from SECCM despite low conversion ratios. The workflow described overcomes challenges associated with coupling both techniques such as avoiding high concentrations of non-volatile salt and large solvent volumes which are detrimental for sensitive MS analysis. On a broader scale, this study helps define prospects for coupling nanoEC-MS measurements by demonstrating a route for detection and identification of species produced from electrochemical reactions performed at micrometer and submicron levels.

## Experimental Section

### Chemicals and Solutions Preparation

Uric acid (UA, 99% purity) was purchased from TCI (Portland, OR), ammonium acetate (AA, ≥97% purity) was purchased from Avantor (Haryana, India), and hydroxybenzotriazole (HOBT, ACS grade) was purchased from Oakwood Products (Estill, NC). All solutions were prepared using DI water purified with a Barnstead GenPure Pro (resistivity 18.2 MΩ·cm, Thermo Scientific, Waltham, MA). Analyte solution consisted of 0.2 mM UA, 1 mM AA used as the supporting electrolyte, and 0.05 mM HOBT, which served as an internal reference.

### Bulk Electrochemical Measurements

Bulk cyclic voltammograms (CVs) were measured using an electrochemical workstation (CHI660C, CH Instruments, Bee Cave, TX) with a 3-electrode system at 0.05 V/s. For these measurements, Ag/AgCl was used as reference electrode (CH111, CH Instruments, Bee Cave, TX), Pt mesh as the counter electrode (Huanya, China), and a 1 mm diameter optically transparent carbon electrode (OTCE) was used as the working electrode.^50^

### Nanopipette Preparation

Borosilicate glass capillaries (B-100-58-10, Sutter Instruments, Novato, CA) were pulled by using a puller (model P-1000, Sutter Instruments). Pulling was optimized in two separate programs to generate glass nanopipettes with an I.D. of either 900 nm or 3 μm. Nanopipette tip size was characterized by SEM ().

### SECCM Measurements

CVs were also obtained with a home-built SECCM with a 2-electrode system and scanned at 1 V/s. A Ag/AgCl electrode was used as the quasi-reference counter electrode (QRCE) which was prepared by chloridizing a Ag wire (WPI, Sarasota, FL) with FeCl_3_. An OTCE surface coming from the same batch as that used for bulk electrochemical measurements served as the working electrode. SECCM current was measured using a current amplifier (Axopatch 200B, Molecular Devices, San Jose, CA), and the nanopipette probe was filled with the analyte solution. Coarse positioning was achieved with an XYZ stepper motor (MMP50, MCL, Madison, WI). An XYZ-piezo (Nano 3D200, MCL) was used for fine control and to approach the probe to the substrate electrode. The SECCM was controlled by a custom programmed LabVIEW (National Instruments, Austin, TX) interface that controlled a field-programmable gate array system (NI 9147, NI 9220, NI 9264, National Instruments) to collect data and control the piezo. The scanning electrochemical system was built atop a total internal reflection microscope (Cairn Research, Faversham, UK).

### Mass Spectrometry

Mass spectra were acquired using an Orbitrap Elite mass spectrometer instrument (Thermo Fisher Scientific, San Jose, CA) operated in negative ion mode. A platinum wire (0.50 mm diameter, WPI) was used to provide high voltage to the nanopipette for nESI. Samples were ionized by the application of 1.2-1.5 kV to the Pt wire. The capillary temperature was set to 275°C, and data was acquired in a mass range of *m/z* 100-300 using 2 micro scans and a maximum injection time of 500 ms. Collision induced dissociation (CID) with a normalized collision energy of 25-35 (arbitrary units) was used to perform tandem mass spectrometry (MS^2^) with a mass window of 0.5 in the ion trap. Initial data was analyzed using Xcalibur software, and mechanistic data files were converted to an MZML file by msconvert and processed using homemade Python scripts (Supporting Information).

### Finite Element Model (FEM) Simulation

FEM simulations of SECCM experiments were performed using COMSOL Multiphysics version 6.1. Further details of the SECCM simulations are provided in the Supporting Information.

## Results and Discussion

### SECCM-MS Workflow

The workflow for measuring product ions generated from SECCM using MS is illustrated in Figure 1. Initially, the nanopipette containing the analyte solution is positioned 0.5-1 cm from the mass spectrometer inlet to acquire an initial mass spectrum (Figure 1 ①). Next, the nanopipette is transferred to the SECCM setup and the electrochemical reaction is performed, after which the nanopipette is repositioned back in front of the mass spectrometer inlet (Figure 1 ②). The duration of this period ② including manual movement of the nanopipette, positioning of the tip at the interface, 300 s electrolysis via SECCM, and setting the nanopipette back up at the mass spectrometer interface typically required 8-9 min in total. During this process, the MS remains operational with the same settings and continually collects data to minimize spectrum to spectrum variations. The last step of the workflow (Figure 1 ③) consists of measuring the reaction products formed via SECCM with MS. By comparing the mass spectrum obtained in steps ① and ③, which represent solution composition pre- and post-SECCM, it is possible to determine the extent of product formation from the electrochemical reaction within the nanopipette.

**Figure 1:**
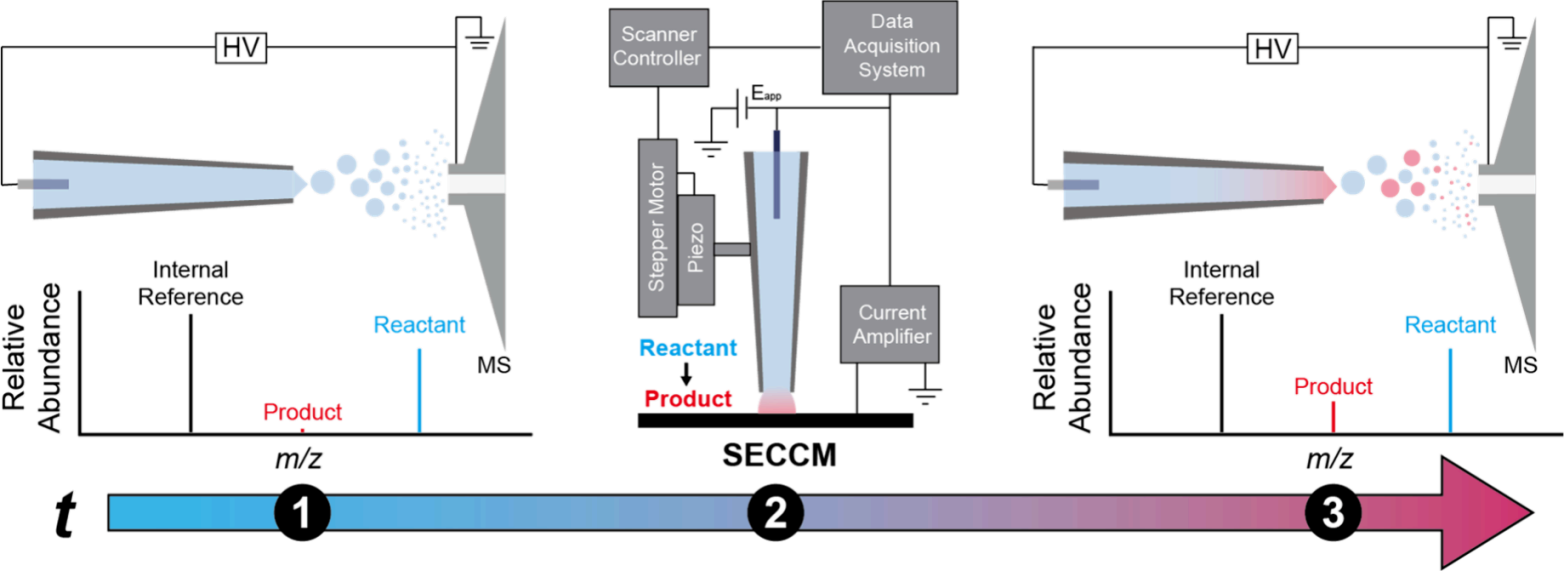
Schematic of SECCM-MS workflow. ① nESI-MS analysis of nanopipette (control). ② SECCM reaction using the same nanopipette to produce electrochemical product. ③ nESI-MS analysis of the same nanopipette post-reaction.

### Oxidation of Uric Acid in Bulk and in SECCM

The oxidation of uric acid (UA) was chosen as a model system to demonstrate the SECCM-MS workflow. Electrochemical oxidation of UA has been previously studied with a high level of attention paid to reaction product characterization by Toth and Yost in an online electrochemistry-MS workflow.^42^ Oxidation of UA **(1)** nominally involves the transfer of 2 protons and 2 electrons to produce 2,6,8-trioxopurine **(2)** as shown in Figure 2a.^51^ The SECCM-MS workflow described here ensures the MS signals obtained at different steps of the workflow are stable through the use of an internal standard hydroxybenzotriazole (HOBT). HOBT is oxidized at higher potentials than UA, does not interfere with the electrolysis of UA, and, most importantly, has a high ionization efficiency, making it a beneficial standard for MS analysis. To determine conditions for oxidative electrolysis of UA, bulk cyclic voltammetry (CV) of a solution containing UA and HOBT in an aqueous ammonium acetate solution (similar to the analyte solution used for SECCM-MS) was performed. In this system, ammonium acetate acts as an electrolyte required for electrochemical measurements and later also functions as an MS-friendly salt. Analyte solutions in ammonium acetate are commonly used for MS experiments because ammonium acetate forms neutral volatile species that do not interfere with analyte signal.^52^ High concentrations of non-volatile salts can result in analyte ion suppression^53^ and ion clustering^54^ which are detrimental for the detection of species generated in ②. The resulting CVs for UA and HOBT alone and in solution together are presented in Figure 2b. Both species display predominantly irreversible electron transfer with anodic peak potentials (E_p,a_) separated by ca. 400 mV (UA: E_p,a_ = 0.45 V, HOBT E_p,a_ = 0.85 V), with HOBT showing a small reductive wave (HOBT, E_p,c_ = 0.50 V). The CV in 0.1 M KCl shown in Figure S9 suggests that ammonium acetate does not affect the electrochemical reaction of UA. CVs collected with SECCM for the same samples are shown in Figure 2c. SECCM CVs were collected in different nanopipettes with slightly different sizes for each solution, and thus current magnitudes are normalized to facilitate comparison. Peak potentials between bulk CVs and SECCM CVS are similar, with the caveat that direct comparison is not possible due to differences in electrode dimensions, scan rates, and corresponding mass transfer conditions but lend confidence to peak assignments and selection of potentials for electrolysis. With these comparative sets of voltammetry, 0.75 V was determined to be a suitable potential to drive the electrolysis of UA, with minimal oxidation of HOBT.

**Figure 2:**
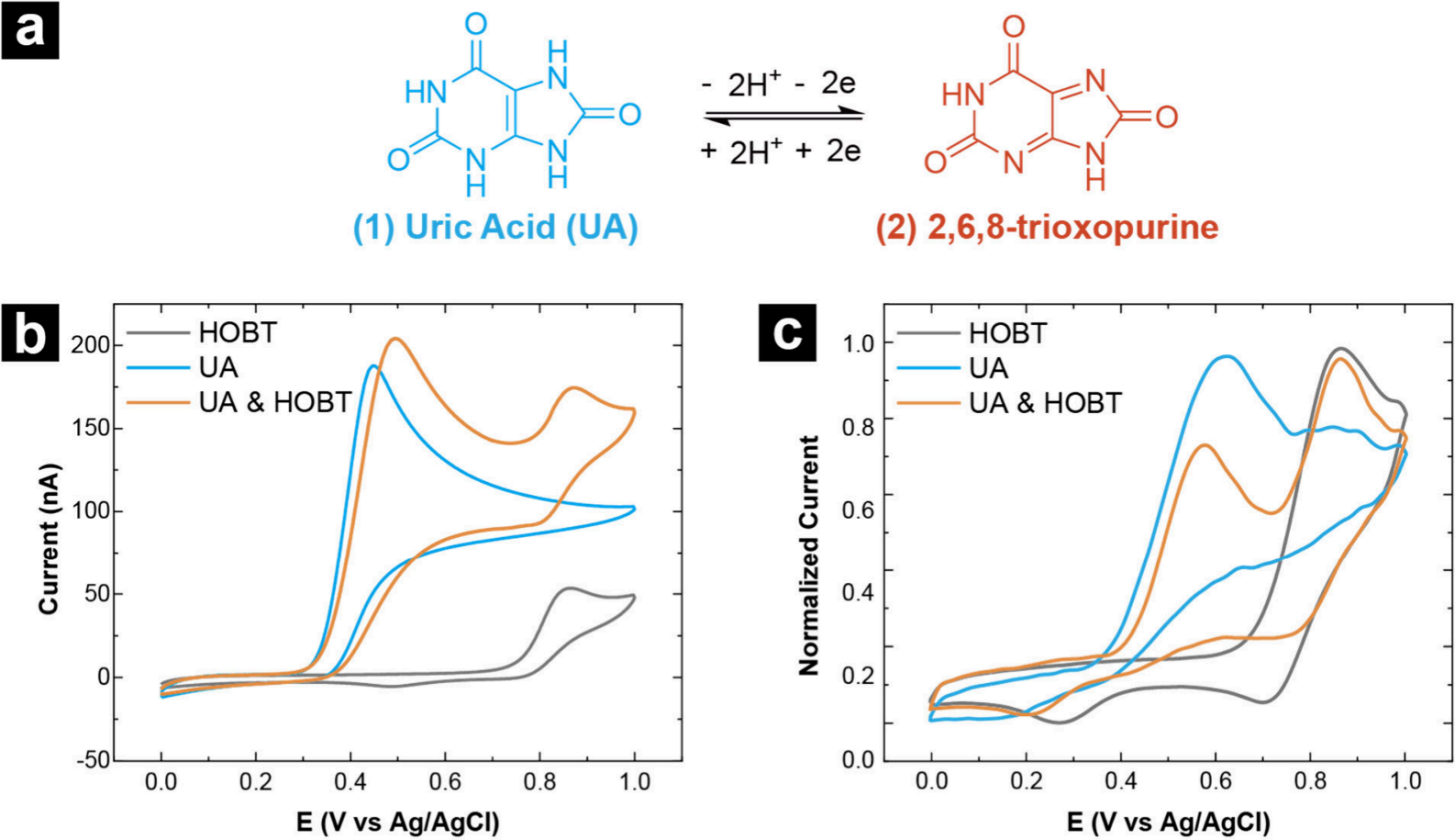
(a) UA oxidation full reaction in water. (b) Macro scale CV of UA and internal reference HOBT. Working electrode: OTCE, Reference electrode: Ag/AgCl, Counter electrode: Pt, Scan rate: 0.05 V/s, Supporting electrolyte: 1 mM AA. (c) SECCM CVs of UA and internal reference HOBT. SECCM probe: 3 μm diameter; working electrode: OTCE, QRCE: Ag/AgCl, scan rate: 1 V/s; Supporting electrolyte: 1 mM AA. Current in (c) is normalized to facilitate comparison between different SECCM probes.

### Detection of Analytes from SECCM with Mass Spectrometry

To examine the SECCM-MS workflow, we performed the oxidation of UA using a 3 μm diameter nanopipette filled with analyte solution. Initially, the nanopipette was placed in front of the mass spectrometer and the mass spectrum was collected for 0.7 min (period ①). After collecting this baseline, the nanopipette was transferred to the SECCM setup to perform the oxidative electrolysis of UA and then positioned back to the MS inlet (period ②). Lastly, mass spectra were collected for 0.8 min (period ③). While mass spectra post-SECCM were acquired for 0.8 min, the most relevant information comes from the first few seconds (3-5 MS scans) of MS analysis because the products are concentrated at the tip of the nanopipette. Figures 3a and 3b show the mass spectrum collected during period ① and the first 0.15 min of period ③, respectively, for a mass range from *m/z* 120 to 180 with the mass spectrum over a wider *m/z* range included in Figure S2. These mass spectra are normalized against the signal of the HOBT internal standard which helps eliminate variations in signal intensity arising from the positioning of the nanopipette with respect to the mass spectrometer inlet. The extracted ion chromatogram (XIC) of the internal reference (HOBT) serves as an indication of overall stability and is shown in Figure S3. The mass spectrum of period ① contains three abundant peaks at *m/z* 134.0361, 166.0137, and 167.0213. These peaks correspond to HOBT, an unidentified species present in the solvent, and reactant **(1)**, UA, respectively. Zooming into the region between *m/z* 165-166 reveals that product **(2)** (*m/z* = 165.0054), the oxidized form of UA, is already present in the solution before the SECCM reaction, which exists as an impurity present in the starting UA reagent, possibly from autooxidation. To confirm this hypothesis, we carried out a high-performance liquid chromatography (HPLC)-MS experiment (Figure S4) of the UA reagent (as received) dissolved in water, which found **(2)** present as a trace component in the standard material. HPLC-MS of the UA standard material in water showed **(2)** with a retention time that differed from UA **(1)** meaning (**2**) is not forming during electrospray (Figure S4). The mass spectrum post-SECCM in Figure 3b shows both HOBT and **(1)** with similar abundances as those in Figure 3a. This observation indicates that the majority of the solution’s composition remains unchanged pre- and post-SECCM, which is expected because the electrochemical reaction is confined to the tip of the nanopipette. Nevertheless, the inset in Figure 3b shows a ∼5-fold increase in the relative abundance of **(2)** post-SECCM which can be attributed to formation of product from the SECCM experiment in period ②, confirming that electrolysis products generated in SECCM can be detected using MS.

**Figure 3:**
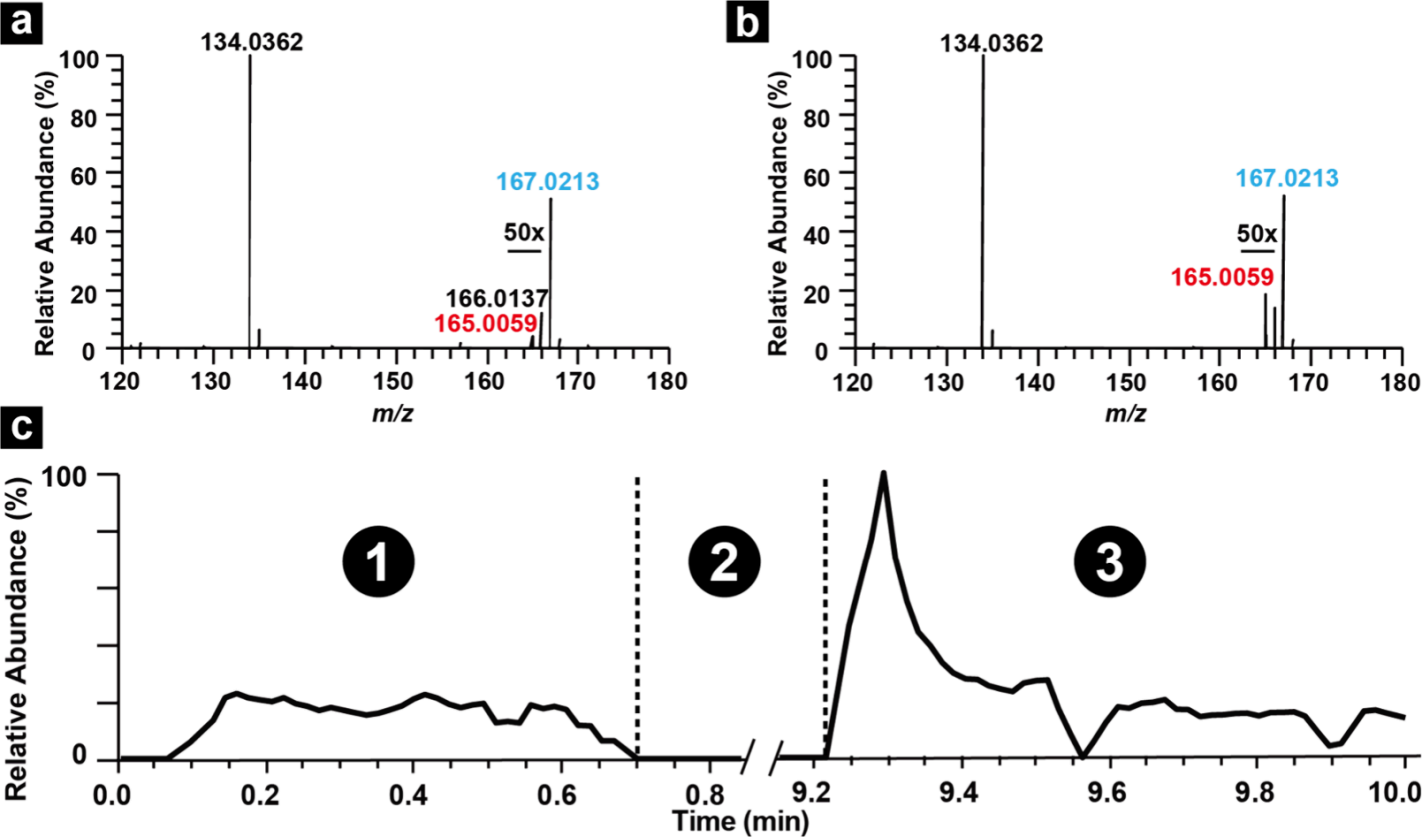
(a) Mass spectrum in period ①. (b) Mass spectrum in period ③. (c) XIC of product **(2)**.

Figure 3c shows the XIC of **(2)** and contains the three periods of the workflow: ① pre-SECCM, ② nanopipette transfer before SECCM, SECCM reaction, and nanopipette transfer after SECCM, as well as ③ post-SECCM. The XIC in moment ① shows a constant relative abundance of **(2)** and indicates that the nESI signal is stable. After the tip is repositioned in front of the mass spectrometer inlet post-SECCM, the XIC of **(2)** displays a sudden increase followed by an exponential decay with time. As mentioned, *vide supra*, the electrolysis step results in a higher concentration of products at the tip of the nanopipette, leading to the spike in relative abundance observed in Figure 3c. As solution is sprayed from the nanopipette, a decrease in the relative abundance of **(2)** is observed (*vide infra* for simulations of concentrations). The XIC of **(2)** during period ③ decays over time to the level pre-SECCM since this species is already present in the solution. The XIC profile in Figure 3c is reproducible as demonstrated by performing the same experiments with at least 30 nanopipettes on different days (Figure S5).

### Detection of Species Produced from SECCM Using MS

An especially compelling reason to employ EC-MS for the study of electrochemical reactions is the opportunity to observe intermediates and follow the chemical reactivity that cannot be revealed in a straightforward manner by electrochemical measurements. A previous study by Volk *et* *al*.^55^ reported that the oxidation of **(1)** to **(2)** is followed by multistep chemical reactions that include ammonolysis, hydrolysis, restructuring, loss of CO_2_, and loss of NH_3_. This reaction mechanism has been previously reported in the literature, and the reaction pathways, intermediates, and products are shown in Figure 4a. The SECCM-MS workflow used for this experiment used water as solvent and ammonium acetate as an electrolyte; therefore, we hypothesize that the reaction mechanism is similar to that proposed by Volk, *et* *al*., who used the same choice of solvent and electrolyte. The scheme in Figure 4a shows that the first step in the reaction mechanism involves the oxidation of **(1)** to **(2)** which is accompanied by the loss of two hydrogen ions. The oxidized product **(2)** can either undergo ammonolysis forming an imine amine **(4)** or hydrolysis to form **(3-1)**. Species **(3-1)** is an imine alcohol that, after restructuring, produces **(3-2)**. Decomposition of **(4)** and **(3-2)** through CHNO and CO_2_ loss, respectively, produces bicyclic imidazoline **(5)**. The hydrolysis of **(5)** produces **(6-1)**, which after restructuring forms allantoin **(6-2)**. Allantoin **(6-2)** is the product commonly known to form from oxidation of **(1)** since species **(2)-(5)** are challenging to isolate in solution.^55–57^

**Figure 4:**
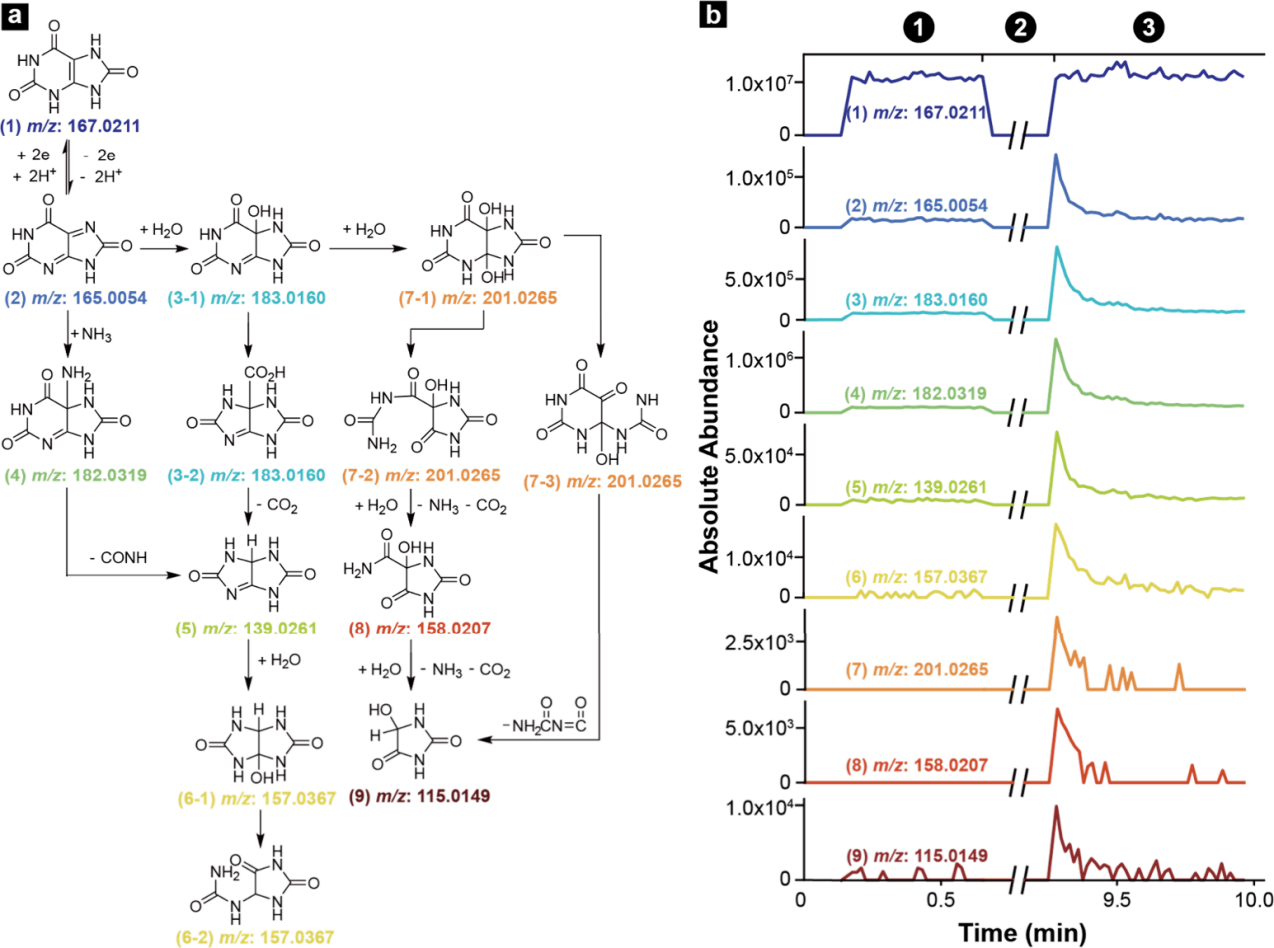
(a) Detailed reaction of UA oxidation in the SECCM. (b) XIC of reactant and detected products.

In addition to the detection of **(2)**, which is formed electrochemically from **(1)**, a closer look into the mass spectra pre- and post-SECCM reveals species **(3)-(6)** which are products formed from the chemical evolution of **(2)** in solution. The mass assignment of species **(3)**-**(6)** was determined based on their accurate masses, and their corresponding XICs are shown in Figure 4b. The XIC of **(1)** in Figure 4b shows that this species has a stable absolute abundance pre- and post-SECCM reaction which suggests the concentration of **(1)** in the nanopipette did not significantly change during the SECCM reaction. The XIC of **(2)** has a distinct XIC compared to the one measured for **(1)**. The XIC of **(2)** shows this ion with low abundance pre-SECCM, followed by a significant increase post-SECCM electrolysis. The exponential decay in the total abundance of **(2)** in period ③ is caused by the concentration gradient, as described earlier. Chemically generated species **(3)**-**(6)** have a concentration profile similar to that shown for **(2)**, a significant increase in abundance post-SECCM with an exponential decay within a few seconds. The spike in absolute abundance followed by an exponential decay in signal indicates that a particular species was formed through SECCM. In contrast, if a species was already present in the SECCM tip and its presence is not attributed to the SECCM step, the abundance should remain constant in ① and ③. Figure S6 shows the results of a control experiment in which experimental conditions were kept the same but without performing the electrolysis step. The XIC profiles in Figure S6 have constant abundances during the entire experiment and do not show the spike and exponential decay characteristic of species formed during the electrolysis step.

The mass spectrum post-SECCM also shows the presence of other species that are not formed through the reaction pathway described above. The XICs in Figure 4b have a similar profile as species **(2)-(6)**, suggesting these species were produced after the electrolysis step. These species have been identified previously in literature and correspond to an alternate pathway in which **(3-1)** is further hydrolyzed to form **(7-1)**.^55^ The restructuring of **(7-1)** produces two different isomers **(7-2)** and **(7-3)**. Species **(7-2)** degrades to form **(8)** after losing CO_2_ and NH_3_ with water gained, and a similar reaction produces 5-hydroxyhydantion **(9)** from **(8)**. Alternatively, **(9)** can also be formed after the loss of NH_2_CONCO from **(7-3)**.

Some species in the reaction mechanism depicted in Figure 4a are isomers that are challenging to distinguish without other characterization techniques. The high resolution of the Orbitrap mass spectrometer allows determination of the exact mass of products, and the CID of these products can then be used to obtain structural isomer identification. Most of the species in Figure 4a have complex fragmentation patterns that also complicate their accurate isomer identification. Nevertheless, species **(6-1)** and **(6-2)** are isomers that have distinct MS^2^ spectra which have been reported previously.^58,59^ The MS^2^ results for **(6)** are shown in Figure S7, and the mass assignments are listed in Table S1. The observed species correspond to the loss of H_2_O, CH_2_NO, CH_4_NO, and CH_4_N_2_O from the precursor ion forming product ions with *m/z* 139.00, 113.00, 111.00, and 97.00, respectively. From these, ions with *m/z* 113.00, 111.00, and 97.00 are expected fragment ions from the **(6-2)** precursor. The ion at *m/z* 139.00 corresponds to a water loss and is not a reported mass loss from **(6-2)**.^58,59^ Such a fragment ion may be produced from the fragmentation of the **(6-1)** precursor which is formed from water addition to **(5)**.

Altogether, the results presented in Figure 4 show that SECCM-MS enables the measurement of electrochemically generated species within a submicron area. The high sensitivity and specificity of MS using this approach further allowed us to detect species formed from the direct oxidation of UA and the ones formed from subsequent chemical reactions in solution, information that could not be obtained by electrochemical means alone.

### Detection of Species Produced from SECCM Using Submicron (Nano)­pipettes

An advantage of SECCM is the ability to tune the nanopipette tip diameter and thus enable control over the reaction surface area. By probing smaller surface areas, the properties of individual particles,^60^ electron transfer processes,^61^ and enhanced resolution in imaging experiments can be achieved.^62,63^ In SECCM-MS, as presented here, the tip diameter has an impact on the distribution of species observed after SECCM. The nanopipette size has a direct influence on the effective meniscus contact diameter, overall resistance of the system,^64,65^ and mass transport rates^4,66–69^ which might decrease the formation yield of reaction products. While the amount of analyte delivered to the mass spectrometer may decrease, nanopipettes used for ion generation in MS can offer advantages for the detection of low-abundant species^70^ such as lower flow rates,^71^ increased signal-to-noise ratio (SNR),^72^ and higher tolerance to buffer composition and salt concentration.^73–75^ Figure 5 shows the XIC of species **(1)**-**(9)** obtained when the oxidation of UA was performed using a 900 nm diameter nanopipette (SEM image of nanopipette shown in Figure S1b). The absolute abundance of these species measured in the first moments of period ③ is approximately 5 times lower when using the 900 nm nanopipette (Figure 5) compared to the measurements with a 3 μm tip (Figure 4). Lower abundances of species **(1)**-**(9)** in Figure 5 suggest a low reaction yield in the 900 nm tip during electrolysis which is likely caused by the relatively small distance over which concentrations are perturbed for nanopipettes. The XICs in Figure 5 also reveal that, for species **(2)-(5)** and **(7)**-**(9)**, the signal post-SECCM is sufficiently higher than the pre-SECCM signal, showing that there was a change in concentration as a result of the SECCM reaction. While the concentration of products in the nanopipette is low in the 900 nm tip, detection using MS is still possible because of the high SNR facilitated by nESI. An approach to establish whether an analyte is produced through SECCM is to determine if the signal post-SECCM is above the average (μ) signal pre-SECCM by three times the standard deviation (σ). In Figure 5 the region enclosed by μ + 3σ is shown by a color-coded, shaded rectangle. This analysis shows that species **(2)**-**(5)** and **(7)**-**(9)** have a relative abundance post-SECCM at ∼8.5 min that is high enough to be above the threshold value. Meanwhile, species **(6)** has roughly the same abundance before and after the SECCM reaction, thus indicating that if this species was formed during the electrolysis step, they could not be detected using MS. Overall, the result from this section demonstrates that most of the reaction products from the oxidation of UA can be detected regardless of the nanopipette size. Furthermore, the high SNR provided by nESI enables the detection of reaction products despite their initial low concentration in the 900 nm tip.

**Figure 5:**
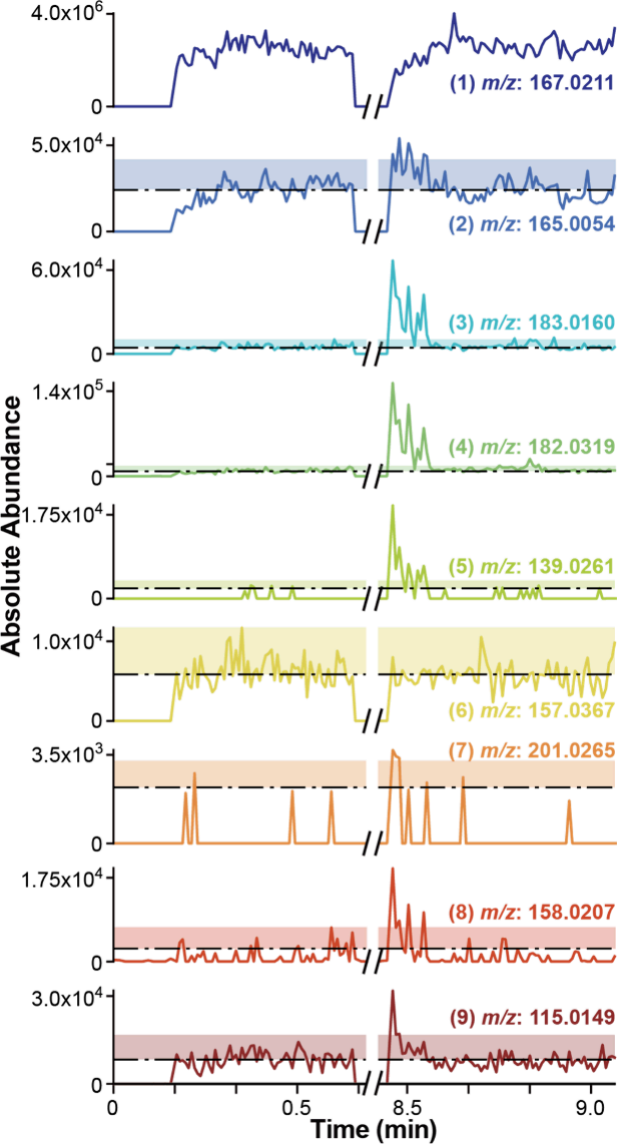
XIC of all reaction related species within a 900 nm diameter nanopipette. The dotted line represents the average absolute abundance pre-SECCM, and the colored area represents the interval deviation of 3σ from the μ of the pre-SECCM signal.

### FEM Simulation

To better understand the amount of product species generated through SECCCM and diffusion in the nanopipette, COMSOL was used to simulate two stages: the SECCM reaction and then diffusion. In the simulation, the reaction was run for five min in a 3 μm nanopipette to mimic the experimental oxidation conditions and then remove the droplet section to let the rest pipette only have diffusion to mimic the pipette transfer period. This resulted in an increased amount of **(2)** at the tip of the nanopipette as shown in Figure 6a and 6b, respectively. These figures show that the concentration of **(2)** gradually decreases from the tip of the nanopipette toward the interior of the pipette, but the slope is less steep after diffusion. Because the reaction initially occurred inside the droplet at the tip of the nanopipette, **(2)** slowly diffuses toward the inside of the nanopipette. After the pipette and droplet are separated, **(2)** continues to diffuse into the pipette. A comparison of the concentration profile of **(2)** (Figure 6d) with its experimentally measured XIC (Figure 6e) shows an agreement between them and demonstrates that the XIC reflects the concentration of **(2)** alongside the vertical axis of the nanopipette since an estimated nESI flow rate at 70 nL/min was measured. Indeed, the sharp increase in the abundance of **(2)** as soon as the nanopipette is placed back in front of the mass spectrometer in period ③ is a result of the accumulation of products at the tip of the nanopipette. As the solution flows from the nanopipette during nESI, the concentration of **(2)** gradually decreases from a high concentration to the pre-SECCM level observed in period ①. Overall, the results of the COMSOL simulation indicate that there is a high correlation between the measured XIC and the concentration profiles in the nanopipette of species generated through SECCM. An interesting and potentially significant caveat to this simulation is the role of wetting and material transfer from the SECCM probe to the surface – described frequently in the literature as a “footprint”. The net result of SECCM footprints is likely loss of solution with the highest concentration – that at the very tip of the pipette – which remains at the interface and is not effectively transferred to the MS. In this instance, detection limits might be improved in the future by taking advantage of surface chemistry to balance interfacial energy to minimize solution loss by contact meniscus adhesion.

**Figure 6:**
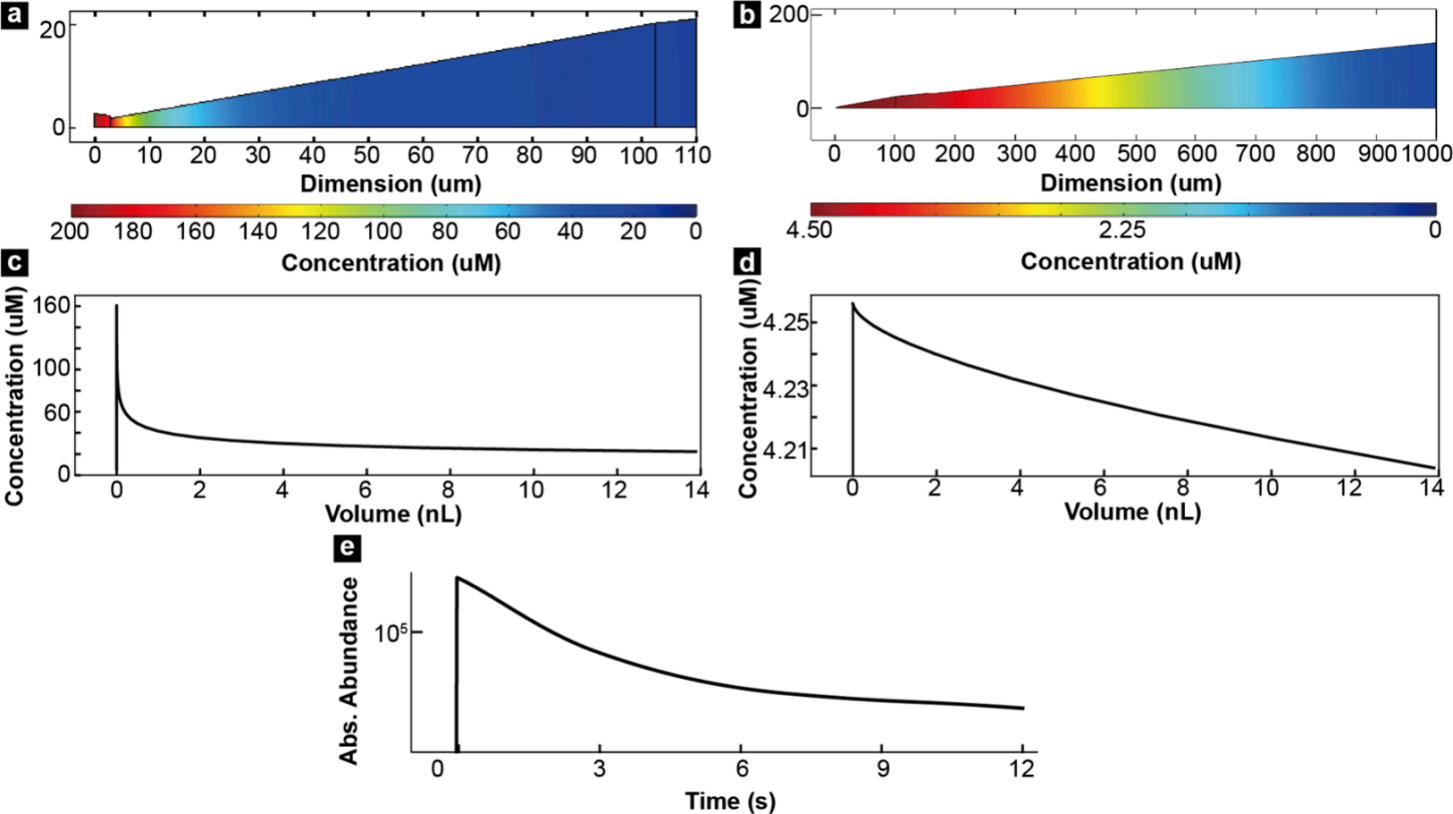
(a) COMSOL simulation of production distribution in nanopipette tip after 300 s SECCM reaction. (b) COMSOL simulation of production distribution in nanopipette tip after 300 s SECCM reaction and 90 s diffusion. (c) Concentration profile along volume from nanopipette tip to up in (a). (d) Concentration profile along volume from nanopipette tip to up in (b). (e) XIC of product in ③.

## Conclusion

In this study, we have developed a novel workflow by combining SECCM with MS to provide complementary characterization of the reaction products produced through SECCM. The main advantage of integrating SECCM with MS is the high specificity for the detection of species formed by an electron transfer process. High sensitivity is achieved by the accumulation of species at the tip of the nanopipette during SECCM and the high SNR offered by nESI-MS. The use of ammonium acetate as electrolyte in SECCM-MS prevents unwanted salt clustering during nESI which otherwise would be detrimental for the detection of species produced through SECCM. In addition, despite the small solution volumes containing the analytes, the ion signal in SECCM-MS is sufficient to perform further structural characterization of the analytes through MS^2^ fragmentation. This work shows that SECCM-MS provides good sensitivity and specificity for the detection of reaction products formed after an electrochemical transformation by using UA as a model system. Species **(2)**-**(9)** are the previously reported species formed following the oxidation of UA in bulk solution, and all these species were detected with SECCM-MS using 3 μm diameter tips. Furthermore, the same species (excluding **(6)**) were detected when a 900 nm tip was used for SECCM-MS, which further demonstrates that the formation and detection of products can be achieved with smaller tip diameters despite lower reaction volumes. In addition, the performed finite element simulations show that the experimentally measured XICs accurately represent the concentration profiles of species along the nanopipette vertical axis. We envision that by further implementation of this workflow characterization of complex reactions occurring in small volumes can be realized. Further, SECCM-MS can be used to detect changes in the distribution of products generated through SECCM which can be used to probe surface composition with sub-micron level spatial resolution.

## Supplementary Material





## Notes

Additional experimental details, materials, and methods, including simulation model reports (PDF)
Simulation model report (PDF)
