# Supplementary material for: Scanning Electrochemical Cell Microscopy for Sub-Micrometer Mass Spectrometric Studies of Electrochemical Reactions
Source: ACS Electrochem. 2025 Apr 21;1(7):1066–75. doi: 10.1021/acselectrochem.5c00095 (PMC12235642; doi:10.1021/acselectrochem.5c00095)
Supplement: Supplementary file 1 [file ec5c00095_si_001.pdf]

Supporting Information

Scanning Electrochemical Cell Microscopy for Sub-micrometer Mass Spectrometric Studies of Electrochemical Reactions

Lingjie Zhang[1], Madison E. Edwards[1], Oluwasegun J. Wahab, Hugo Y. Samayoa-Oviedo, Dallas P. Freitas, Xin Yan* and Lane A. Baker*

Department of Chemistry, Texas A&M University, College Station, Texas 77843

*Corresponding Authors:
Lane A. Baker - Department of Chemistry, Texas A&M University, College Station, Texas 77843
Email: lane.baker@tamu.edu

Xin Yan - Department of Chemistry, Texas A&M University, College Station, Texas 77843
Email: xyan@tamu.edu

Table of Contents

S1. Characterization of Nanopipette…………………………………………………..S-3

S2. Full range mass spectra from Figure 3 …………………..………..………………S-4

S3. Stability of the MS signal …………………..……………………………………...S-5

S4. Purity Test of the Analyte.…………………..…………………….………………S-6

S5. Reproducibility………………………….…………………………………………...S-7

S6. Blank Experiment ….…………………….……………………………………...S-8

S7. MS^2 of 157 m/z ……………………………………………………………………..S-9

S8. Finite Element Method (FEM) Simulations……………………………………..S-10

S9. CV comparison of different electrolyte………………..…………….…………..S-11

S10. Python Code for plotting the XIC and calculating the standard deviation of the signal.………………..………………………………………..…………………....S-12

Supplemental Methods

S1. Characterization of Nanopipettes

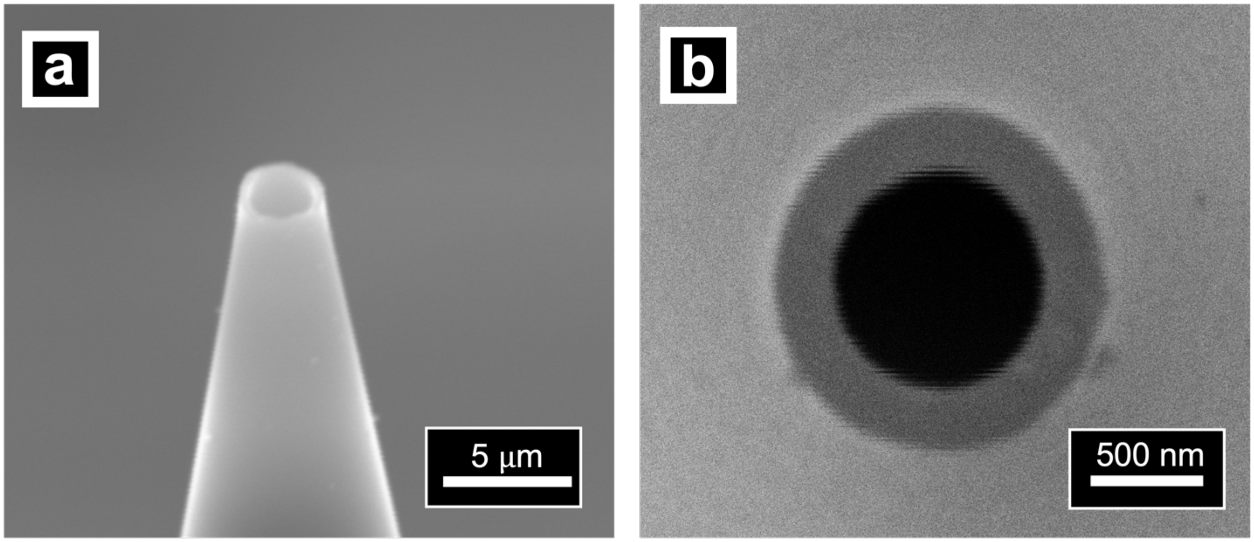

Figure S1. Scanning electron micrographs of (a) the side view of a ~3 µm I.D. borosilicate single barrel nanopipette and (b) the top view of a ~900 nm I.D. borosilicate single barrel nanopipette.

Both sizes of nanopipettes fabricated as described in the methods section showed a consistent geometry.

S2. Full range mass spectra from Figure 3

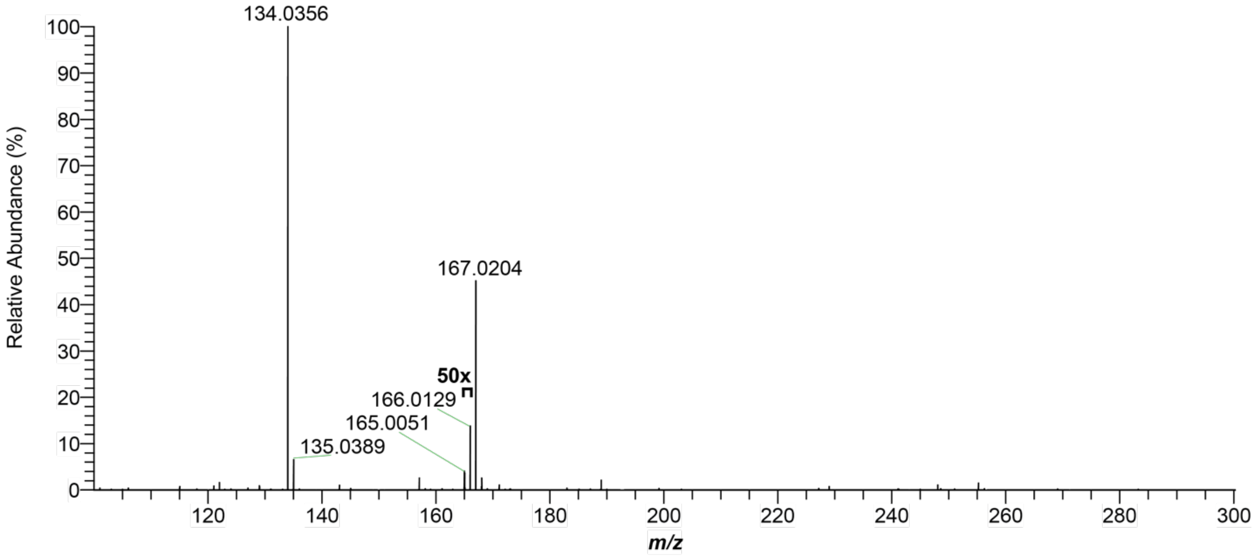

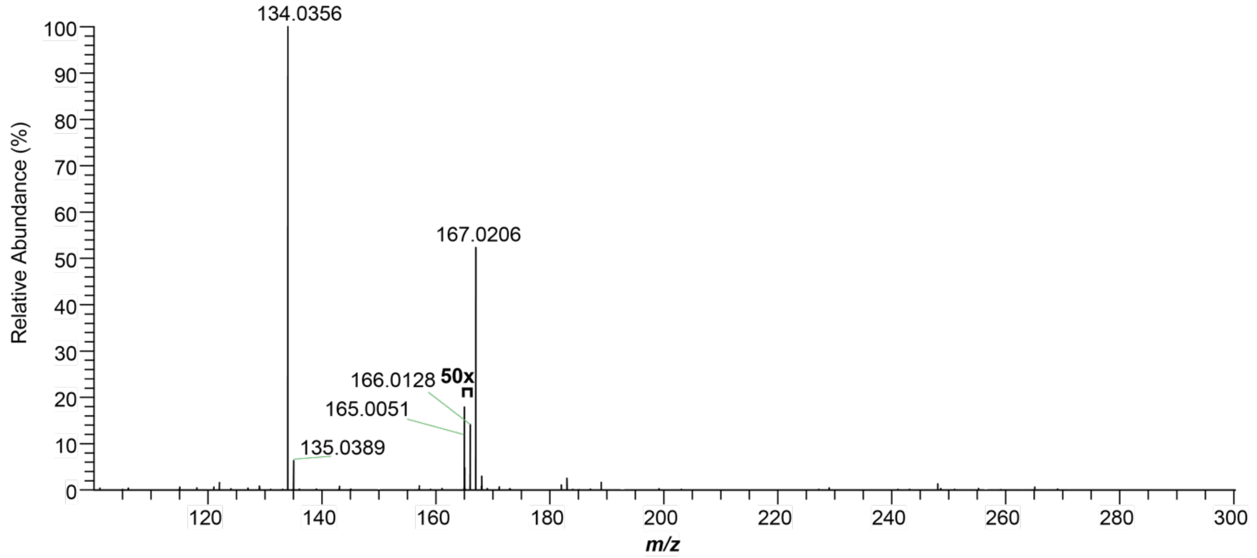

Figure S2. Full range mass spectra from **Figure 3** showing the mass spectrum from moment ❶ shown at the top panel while the bottom panel shows the mass spectrum from moment ❸. The 165-167 *m/z* range is zoomed 50 times to show low abundant species.

S3. Stability of the MS signal

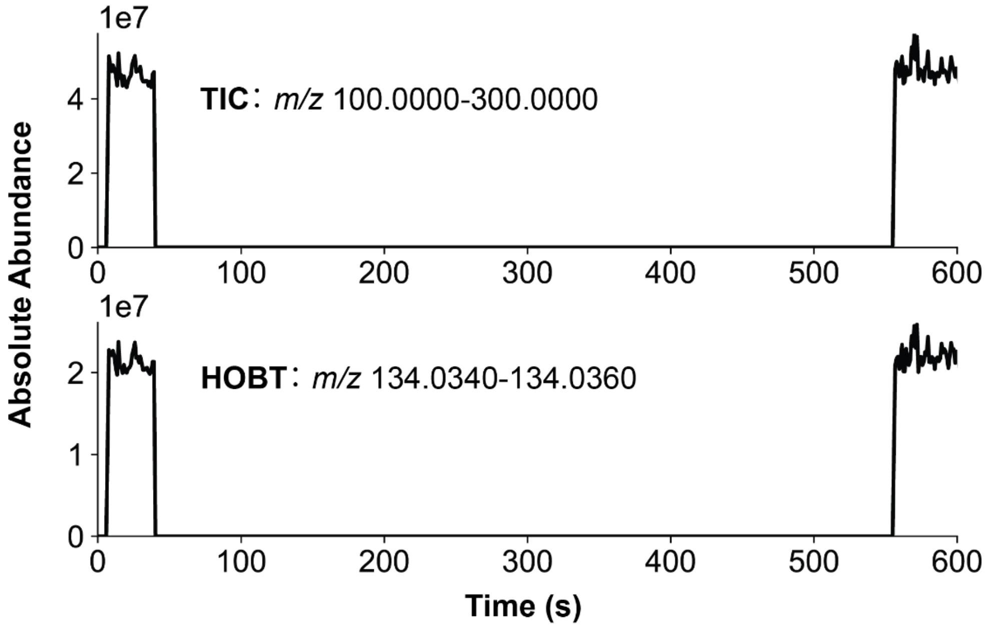

Figure S3. TIC (upper) and XIC of HOBT (bottom) from a 3 μm nanopipette during the whole experiment.

Both the TIC and XIC of HOBT have a stable signal pre- and post-SECCM, which demonstrate the stability of the signal during the whole SECCM-MS workflow.

S4. Purity Test of the Analyte

An Ultimate 3000 UHPLC system (Thermo Fisher Scientific) coupled with a Q Exactive orbitrap mass spectrometer (Thermo Fisher Scientific) was applied for LC-MS analysis. An aliquot of 2 μL of sample was injected into an Accucore™ 150 Amide HILIC HPLC Columns (Thermo Fisher Scientific). The sampler draw speed was set to 5 μL/s, sampler dispense speed was set to 20 μL/s, sampler wash time was 5 sec with a wash speed of 32 μL/s, and sampler temperature was set to 5 °C.

The mobile phase used water with 10 mM ammonium acetate (solvent A) and acetonitrile (solvent B). The column separation was carried out at 30 °C (column temperature) with a flow rate of 0.2 mL/min. The elute gradient was optimized as follows: 90% B at 0-3 min, 90-40% B at 3-20 min, 40-90% B at 20-20.1 min, and held at 90% B at 20.1-30 min.

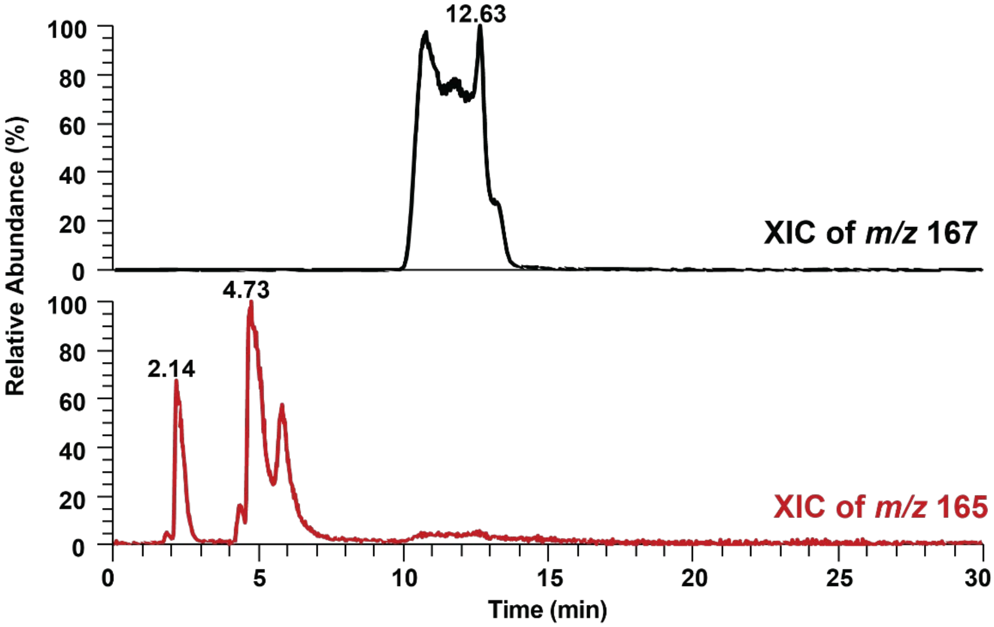

Figure S4. HPLC-MS results from the analysis of a solution of uric acid prepared in HPLC grade water. The upper panel shows the XIC of **(1)** and the bottom shows the XIC of **(2).**

To demonstrate that the presence of **(2)** in moment ❶ (pre-SECCM) arises from contamination in the reagent bottle, we performed an HPLC-MS experiment with the results shown in **Figure S4**. The chromatograms show that product **(2)** is observed

minutes before the reagent **(1)**, thus confirming that **(2)** is not formed from **(1)** and instead is a separate component in the sample mixture.

S5. Reproducibility

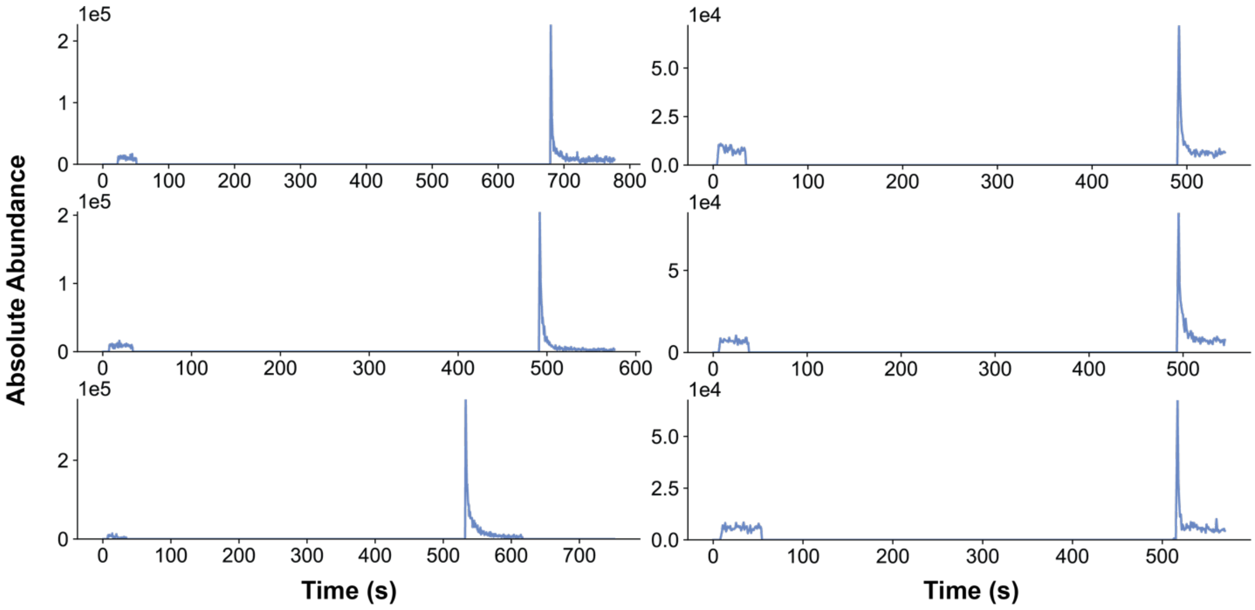

Figure S5. XIC of **(2)** from multiple nanopipettes.

To show the reproducibility of our SECCM-MS workflow from pipette to pipette, we performed the workflow with thirty different pipettes, in different days, and 83% pipettes worked. In **Figure S5** we randomly choose and show 6 XIC data of **(2)** among the 83%, these data shows high reproducibility.

S6. Blank Experiment

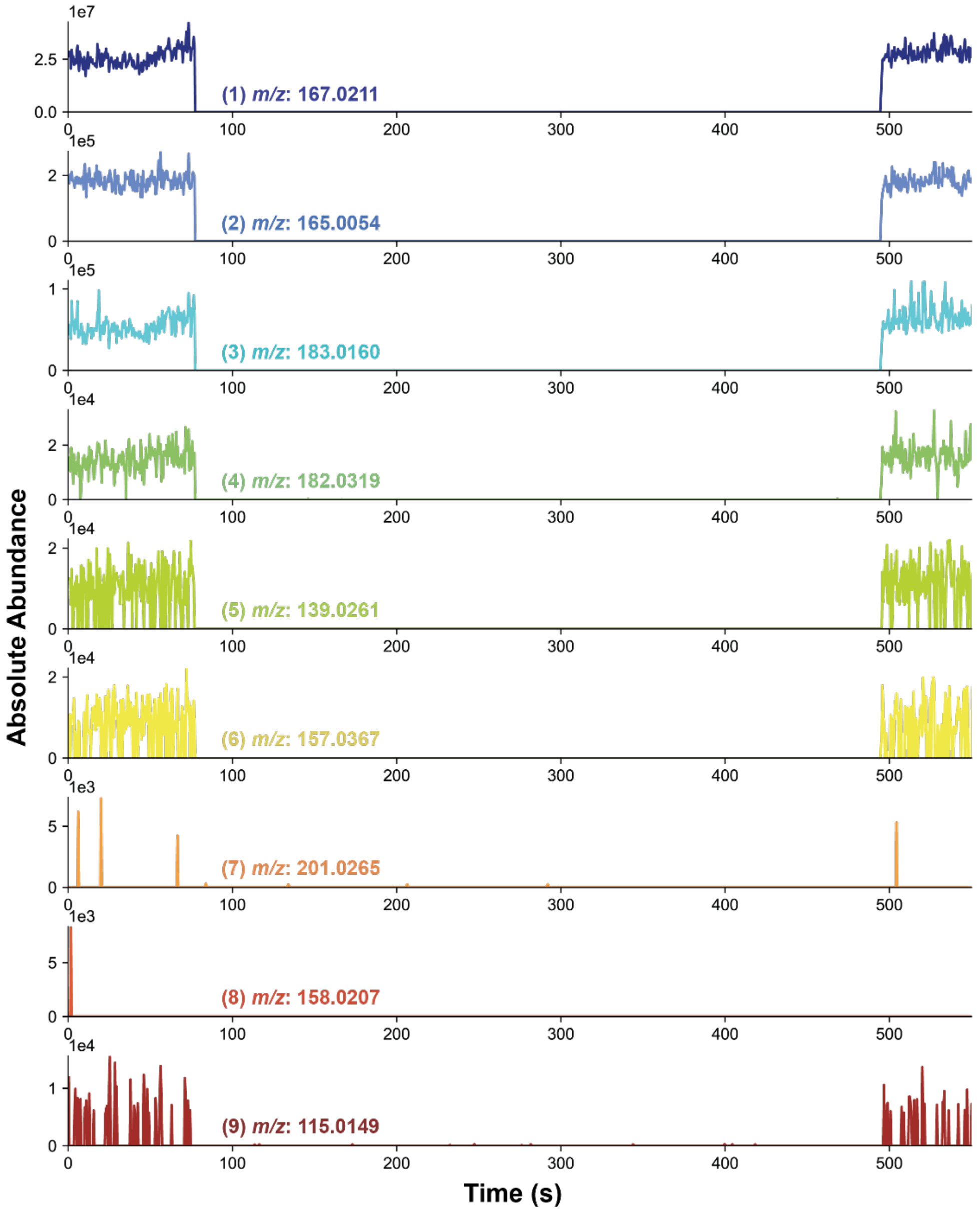

Figure S6. XIC of all intermediates and products of a blank experiment.

To confirm that the increase in the relative abundances of intermediates and products is caused by the SECCM reaction, we designed and performed a blank experiment. In the blank, we keep all the conditions the same as in **Figure 1** but without performing electrolysis on the surface in moment ❷. **Figure S6** shows that, when no electrolysis is performed, the relative abundances of all the species remain the same pre- and post-SECCM.

S7. MS^2 of 157 *m/z*

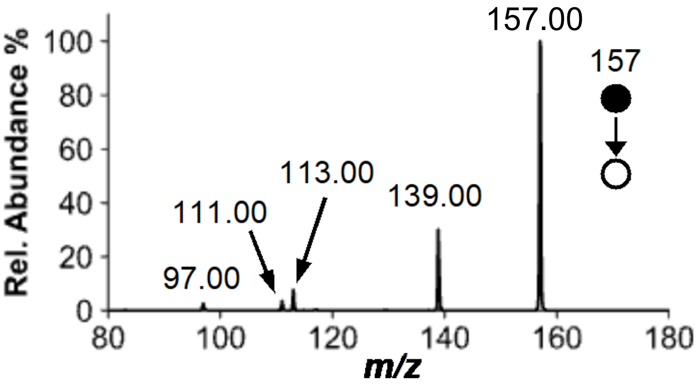

Figure S7. MS^2 of *m/z* at 157.

Table S1. Mass assignments of *m/z*.

m/z	**Assignment**
**139.00**	**$[C_4H_3N_4O_2]^-$**
**113.00**	**$[C_3H_3N_3O_2]^-$**
**111.00**	**$[C_3HN_3O_2]^-$**
**97.00**	**$[C_3HN_2O_2]^-$**

S8. Finite Element Method (FEM) Simulations

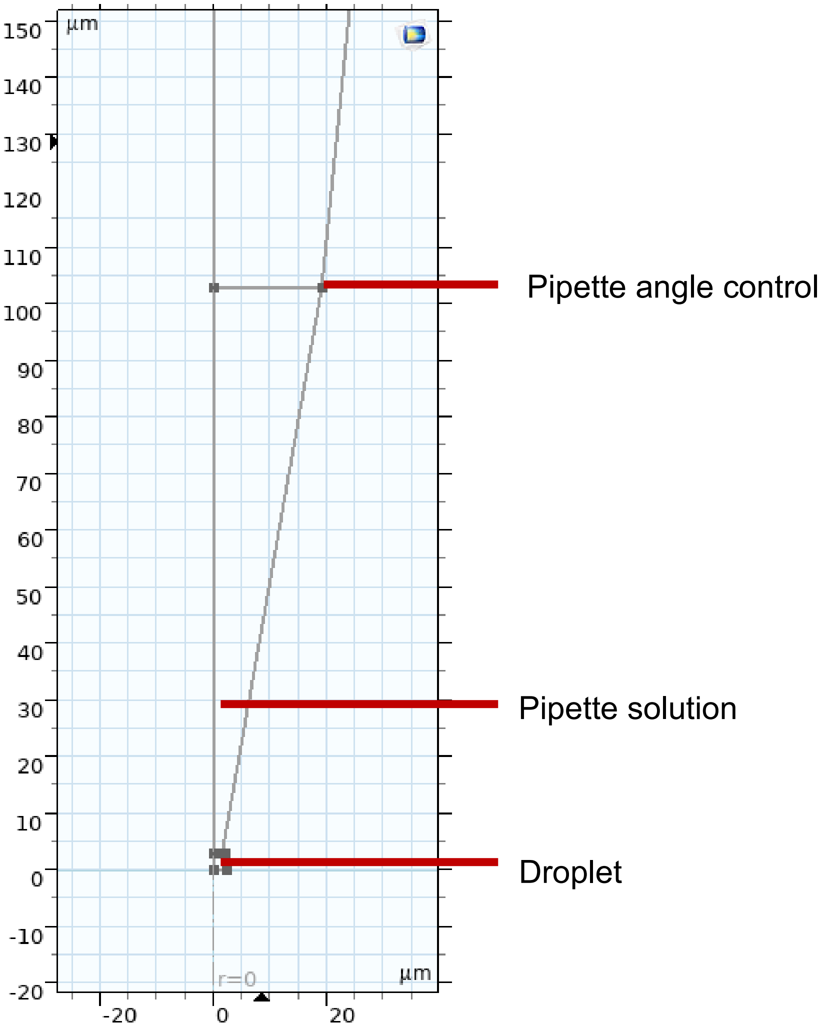

Figure S8. 2D axis symmetric geometric setup of the SECCM nanopipette in COMSOL.

Finite element method simulations of SECCM were performed using COMSOL Multiphysics v6.1 based on the model previously reported by Wahab, O. J.; *et.al.*[1] and the experimental conditions of the nanopipette tips produced in this work.

S9. CV comparison of different electrolyte

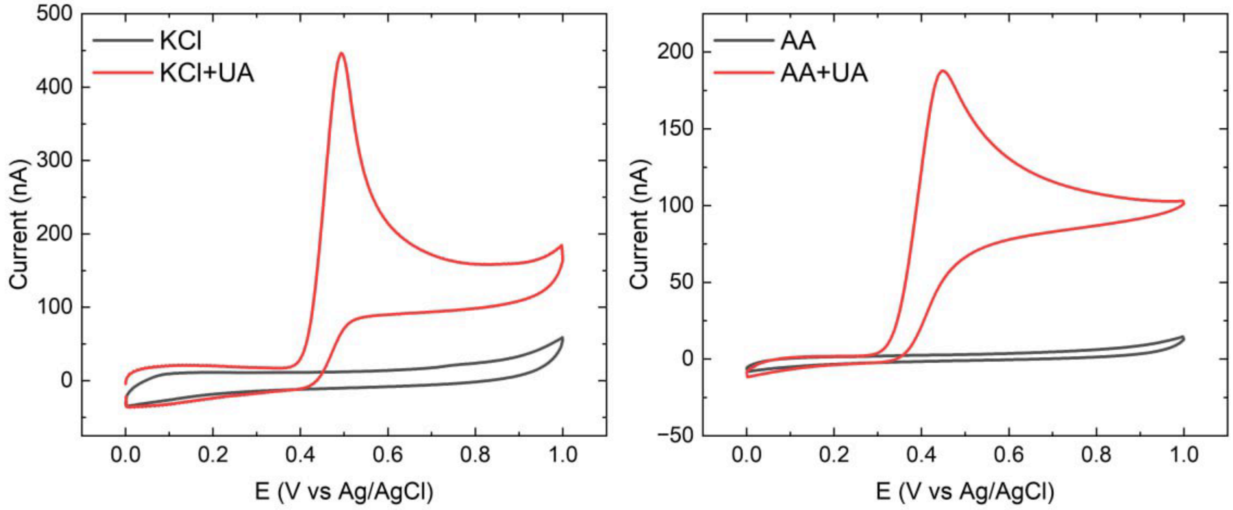

Figure S9. Left is CV of 0.1 M KCl and 0.1 M KCl with 0.2 mM UA. Right is CV of 1 mM AA and 1 mM AA with 0.2 mM UA. All CVs measured with OTCE as working electrode, Ag/AgCl as reference electrode and Pt as counter electrode. Scan rate is 0.05 V/s.

S10. Python Code for plotting the XIC and calculating the standard deviation of the signal

```
import matplotlib.pyplot as plt
import numpy as np
import pyopenms

# define the mz range, the start and end of the blank, and the filename here
mz_range = (167.0204, 167.0206)
blank_start = 10
blank_end = 70
norm = False
filename = "./pipette1.mzML"


def read_mzml(filename):
    exp = pyopenms.MSExperiment()
    pyopenms.MzMLFile().load(filename, exp)
    return exp


exp = read_mzml(filename)
scans = exp.size()

full_rt = []
full_int = []
for spec in exp:
    rt = spec.getRT()
    full_rt.append(rt)
    mz, intensity = spec.get_peaks()
    sum_int = 0
    for i in range(len(mz)):
        if mz[i] >= mz_range[0] and mz[i] <= mz_range[1]:
            sum_int += intensity[i]

    tic = np.sum(intensity)

# normalize the intensity
if norm is True:
    full_int = np.array(full_int)
    full_int = full_int / np.max(full_int)
    plt.ylim(0, 1)
else:
    full_int = np.array(full_int)
    plt.ylim(0, np.max(full_int))


plt.plot(full_rt, full_int, color="black")
```

```
plt.gca().spines["top"].set_visible(False)
plt.gca().spines["right"].set_visible(False)
plt.gcf().set_size_inches(8, 2)
plt.ticklabel_format(axis="y", style="sci", scilimits=(0, 0))

plt.rcParams["font.sans-serif"] = "Arial"
plt.rcParams["font.family"] = "sans-serif"
plt.rcParams["font.size"] = 12

blank_int = full_int[blank_start:blank_end]
blank_int_without_0 = []
for i in blank_int:
    if i != 0:
        full_int_without_0.append(i)

ave = np.mean(blank_int_without_0)
sgima = np.std(blank_int_without_0)

plt.axhline(y=ave, linestyle="-.")
plt.axhline(y=ave + 3 * sgima, linestyle="--")
# plt.axhline(y=ave - 3 * sgima, linestyle="--")

plt.show()
```

References

(1) Wahab, O. J.; Kang, M.; Meloni, G. N.; Daviddi, E.; Unwin, P. R. Nanoscale Visualization of Electrochemical Activity at Indium Tin Oxide Electrodes. *Anal. Chem.* **2022**, *94* (11), 4729–4736. https://doi.org/10.1021/acs.analchem.1c05168.
