# Supplementary material for: Scanning Electrochemical Cell Microscopy for Sub-Micrometer Mass Spectrometric Studies of Electrochemical Reactions
Source: ACS Electrochem. 2025 Apr 21;1(7):1066–75. doi: 10.1021/acselectrochem.5c00095 (PMC12235642; doi:10.1021/acselectrochem.5c00095)
Supplement: Supplementary file 2 [file ec5c00095_si_002.pdf]

Supporting Information: COMSOL model report

Scanning Electrochemical Cell Microscopy for Sub-micrometer Mass Spectrometric Studies of Electrochemical Reactions

Lingjie Zhang[1], Madison E. Edwards[1], Oluwasegun J. Wahab, Hugo Y. Samayoa-Oviedo, Dallas P. Freitas, Xin Yan* and Lane A. Baker*

Department of Chemistry, Texas A&M University, College Station, Texas 77843

*Corresponding Authors:
Lane A. Baker - Department of Chemistry, Texas A&M University, College Station, Texas 77843
Email: lane.baker@tamu.edu

Xin Yan - Department of Chemistry, Texas A&M University, College Station, Texas 77843
Email: xyan@tamu.edu

Comsol 6.1

Report date	Mar 31, 2025, 12:52:48 AM

Contents

1. Global Definitions....4
1.1. Parameters....4
1.2. Shared Properties....6
2. Component 1....7
2.1. Definitions....7
2.2. Geometry 1....9
2.3. Materials....11
2.4. Transport of Diluted Species....16
2.5. Mesh 1....31
3. Study 1....33
3.1. SECCM....33
3.2. Pipette Transfer....35
3.3. Solver Configurations....38
4. Results....79
4.1. Datasets....79
4.2. Derived Values....84
4.3. Tables....85
4.4. Plot Groups....87

1 Global Definitions

Date	Mar 29, 2025, 9:52:06 PM

GLOBAL SETTINGS

Name	2Step.mph
Path	/home/zlj/2Step.mph
Version	COMSOL Multiphysics 6.1 (Build: 252)
Unit system	SI

USED PRODUCTS

Chemical Reaction Engineering Module
COMSOL Multiphysics

COMPUTER INFORMATION

CPU	AMD EPYC 7402P 24-Core Processor, 24 cores, 124.89 GB RAM
Operating system	Linux

1.1 PARAMETERS

PARAMETERS 1

**Name**	**Expression**	**Value**	**Description**
Dh	2*Tr	3E−6 m	Droplet height
Tr	1.5[um]	1.5E−6 m	Radius of nanopipet
Dr	1.6*Tr	2.4E−6 m	Radius of droplet
theta	10[deg]	0.17453 rad	pipet cone angle
Tt	500[nm]	5E−7 m	nanopipet edge thickness
Doxi	6.97e-6 [cm^2/s]	6.97E−10 m^2/s	Diffusion coefficient of oxidized species - Resazurin (RZ)
Dred	6.97e-6 [cm^2/s]	6.97E−10 m^2/s	Diffusion coefficient of reduced species - Resorufin (RF)
Rconst	R_const	8.3145 J/(mol·K)	Gas Constant
F	F_const	96485 C/mol	Faraday's Constant
T	298 [K]	298 K	Absolute Temp
alpha	0.5	0.5	Transfer Coefficent
k0	10 [cm/s]	0.1 m/s	Standard rate constant
E0	0.5 [V]	0.5 V	Formal Potential for reduction to RF
C0	0.2[mmol/L]	0.2 mol/m^3	Initial Concentration

Name	Expression	Value	Description
Eapp	0.75[V]	0.75 V	Pulse potential for reaction
t	5*60[s]	300 s	duration
n	2	2	number of electron for RZ to RF reduction
iapp	60[mA/cm^2]	600 A/m²	Current density
Bw	100[um]	1E−4 m	solution box width
Bh	Bw	1E−4 m	solution box height
d	IR0*2	3E−6 m	probe-substrate distances parameter
ang_o	0.06[rad]	0.06 rad	pipette semiangle
ang_in	0.17[rad]	0.17 rad	
lp_h	100.5[um]	1.005E−4 m	
factor	1.0	1	(MINEQL value)
lp	0.5[cm]	0.005 m	
fmw	100[nm]	1E−7 m	
ang_in_2	0.10[rad]	0.1 rad	
ang_o_2	ang_in_2 + 3[deg]	0.15236 rad	
lin	500[um]	5E−4 m	
IR0	1500[nm]	1.5E−6 m	
IR100	1.4*IR0	2.1E−6 m	
IR1000	1.6*IR0	2.4E−6 m	
IR10000	4.8*IR0	7.2E−6 m	
IR100000	IR50000 + ((100[um] - 50[um])*tan(ang_in_2))	1.9083E−5 m	
IR200	1.45*IR0	2.175E−6 m	
IR2000	1.66*IR0	2.49E−6 m	
IR50	1.2*IR0	1.8E−6 m	
IR500	1.5*IR0	2.25E−6 m	
IR5000	3*IR0	4.5E−6 m	
IR50000	IR10000 + ((40[um])*tan(ang_in))	1.4066E−5 m	
IRlp	OR100000 + ((lp - 100[um])*tan(ang_in_2))	5.0785E−4 m	
OR0	1.2*IR0	1.8E−6 m	
OR100	1.3*IR100	2.73E−6 m	
OR1000	1.6*IR1000	3.84E−6 m	
OR10000	1.5*IR10000	1.08E−5 m	

Name	Expression	Value	Description
OR100000	OR50000 + ((100[um] - 50[um])*tan(ang_o))	1.6206E−5 m	
OR200	1.4*IR200	3.045E−6 m	
OR2000	1.5*IR2000	3.735E−6 m	
OR50	1.21*IR50	2.178E−6 m	
OR500	1.5*IR500	3.375E−6 m	
OR5000	1.25*IR5000	5.625E−6 m	
OR50000	OR10000 + ((50[um] - 10[um])*tan(ang_o))	1.3203E−5 m	
ORlp	OR100000 + ((lp - 100[um])*tan(ang_o_2))	7.686E−4 m	
wf	1.5	1.5	wetting factor
cap_rad	0.35[mm]	3.5E−4 m	
cap_length	4.5[cm]	0.045 m	
elec_pos	3[cm]	0.03 m	
perm	78	78	Relative permittivity water 25 degrees

1.2 SHARED PROPERTIES

1.2.1 Default Model Inputs

Tag	cminpt

2 Component 1

Date	Feb 26, 2023, 5:56:07 PM

SETTINGS

Description	Value
Unit system	Same as global system (SI)
Geometry shape function	Automatic

SPATIAL FRAME COORDINATES

First	Second	Third
r	phi	z

MATERIAL FRAME COORDINATES

First	Second	Third
R	PHI	Z

GEOMETRY FRAME COORDINATES

First	Second	Third
Rg	PHIg	Zg

MESH FRAME COORDINATES

First	Second	Third
Rm	PHIm	Zm

2.1 DEFINITIONS

2.1.1 Variables

Variables 1

SELECTION

Geometric entity level	Entire model

Name	Expression	Unit	Description
Koxi	k0*exp((1 - alpha)*n*F*(Eapp - E0)/(Rconst*T))	m/s	
Kred	k0*exp((-alpha)*n*F*(Eapp - E0)/(Rconst*T))	m/s	

2.1.2 Probes

Boundary Probe 1

Probe type	Boundary probe

SELECTION

Geometric entity level	Boundary
Selection	Geometry geom1: Dimension 1: Boundary 2

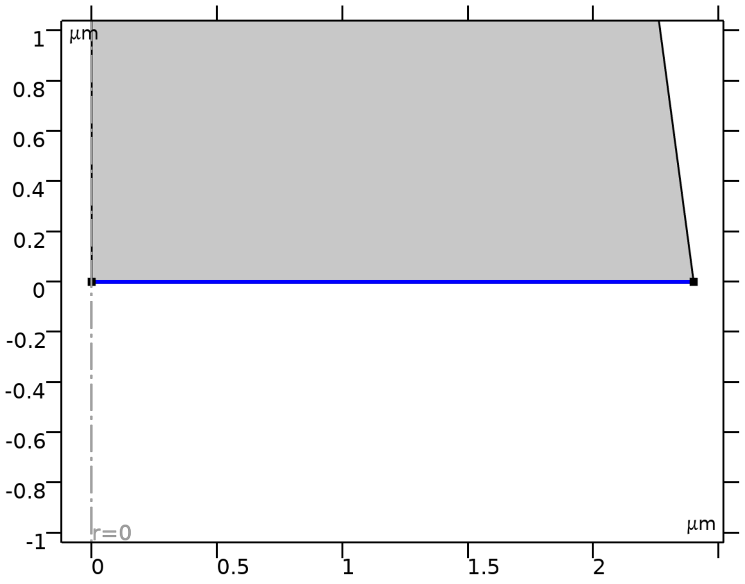

Selection

PROBE TYPE

**Description**	**Value**
Type	Integral

EXPRESSION

**Description**	**Value**
Expression	(tds.ntflux_Cox)*F*2
Table and plot unit	nA
Description	(tds.ntflux_Cox)*F*2

TABLE AND WINDOW SETTINGS

**Description**	**Value**
Output table	Probe Table 1
Plot window	Probe Plot 1

2.1.3 Coordinate Systems

Boundary System 1

Coordinate system type	Boundary system
Tag	sys1

COORDINATE NAMES

First	Second	Third
t1	to	n

SETTINGS

Description	Value
Frame	Geometry configuration
Axis	z

2.2 GEOMETRY 1

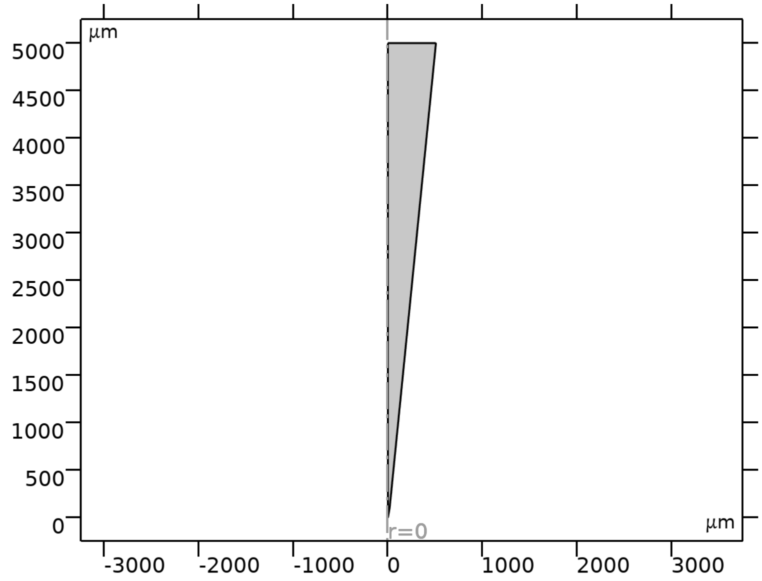

Geometry 1

UNITS

Length unit	µm
Angular unit	deg

GEOMETRY STATISTICS

Description	Value
Space dimension	2
Number of domains	3
Number of boundaries	11
Number of vertices	9

2.2.1 Lower pipet_level 1 (pol6)

SELECTIONS OF RESULTING ENTITIES

Description	Value
Resulting objects selection	On

OBJECT TYPE

Description	Value
Type	Solid

COORDINATES

Description	Value
Data source	Table

COORDINATES

r (µm)	z (µm)
0	Dh
Tr	Dh
IR100000	100000[nm]+Dh
0	100000[nm]+Dh

2.2.2 Lower level 2 (pol4)

SELECTIONS OF RESULTING ENTITIES

Description	Value
Resulting objects selection	On

OBJECT TYPE

Description	Value
Type	Solid

COORDINATES

Description	Value
Data source	Table

COORDINATES

r (µm)	z (µm)
0	100000[nm]+Dh
IR100000	100000[nm]+Dh
IRlp	0.5[cm]+Dh
0	0.5[cm]+Dh

2.2.3 Cornerd droplet (pol2)

SELECTIONS OF RESULTING ENTITIES

Description	Value
Resulting objects selection	On

OBJECT TYPE

Description	Value
Type	Solid

COORDINATES

Description	Value
Data source	Table

COORDINATES

r (µm)	z (µm)
0	Dh
Tr	Dh
Tr+Tt	Dh
Dr	0
0	0

2.3 MATERIALS

2.3.1 Water

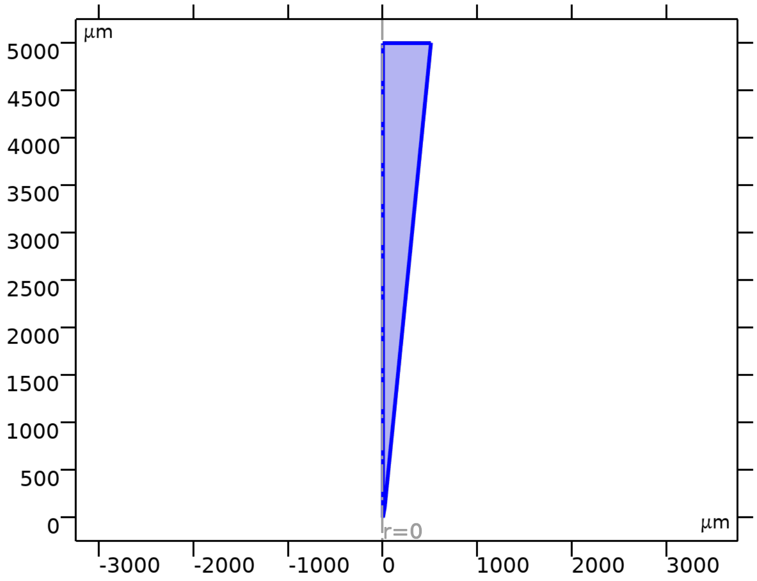

Water

SELECTION

Geometric entity level	Domain

Selection	Geometry geom1: Dimension 2: All domains

BASIC

Description	Value	Unit
Coefficient of thermal expansion	alpha_p(T)	1/K
Bulk viscosity	muB(T)	Pa·s
Dynamic viscosity	eta(T)	Pa·s
Ratio of specific heats	gamma_w(T)	1
Electrical conductivity	5.5E-6	S/m
Heat capacity at constant pressure	Cp(T)	J/(kg·K)
Density	rho(T)	kg/m^3
Thermal conductivity	k(T)	W/(m·K)
Speed of sound	cs(T)	m/s

FUNCTIONS

Function name	Type
eta	Piecewise
Cp	Piecewise
rho	Piecewise
k	Piecewise
cs	Interpolation
alpha_p	Analytic
gamma_w	Analytic
muB	Analytic

Piecewise

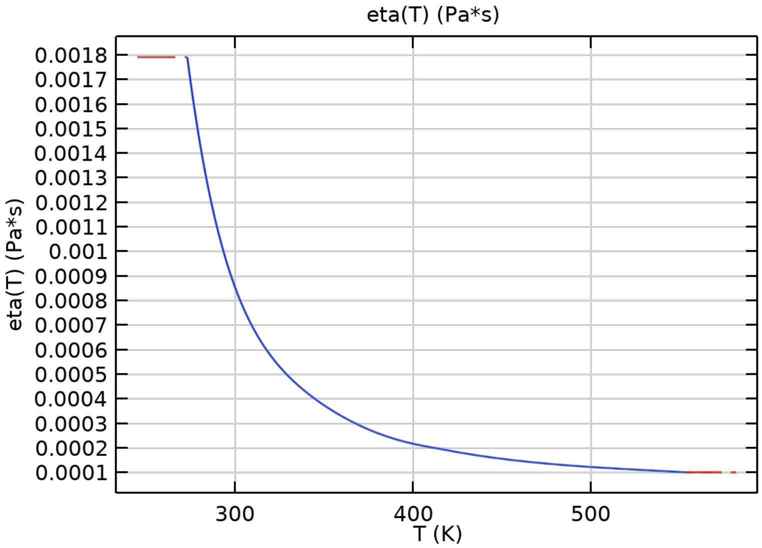

eta

Piecewise 2

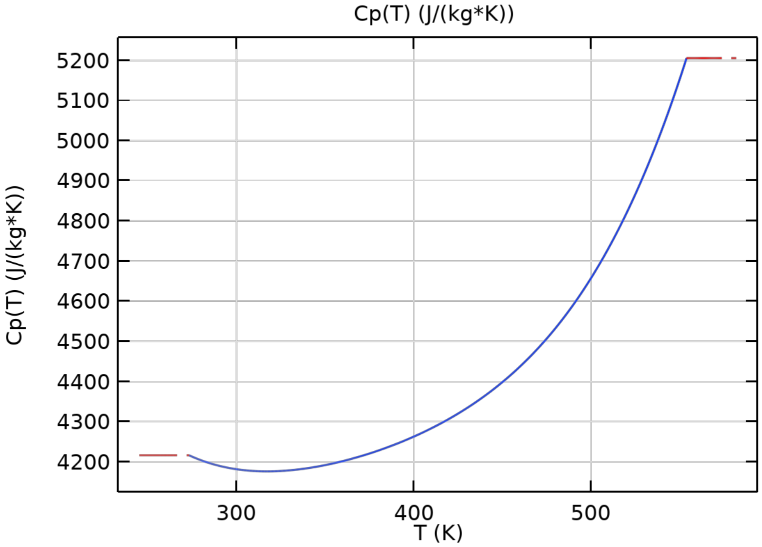

Cp

Piecewise 3

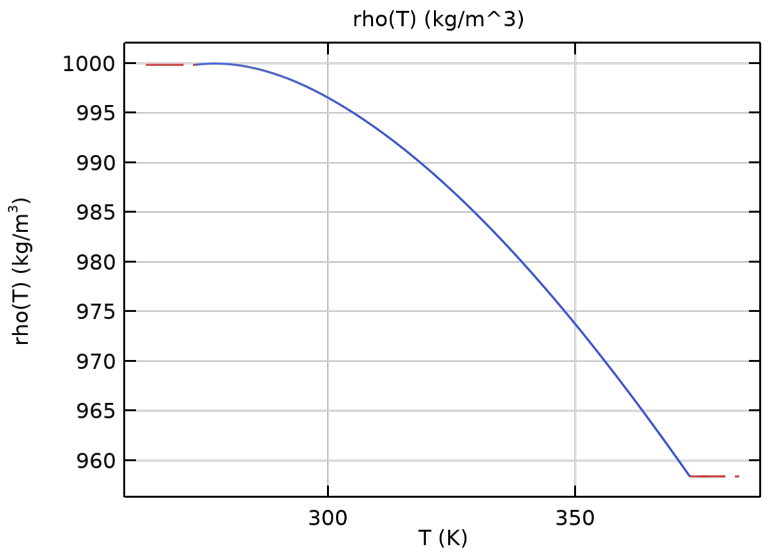

rho

Piecewise 4

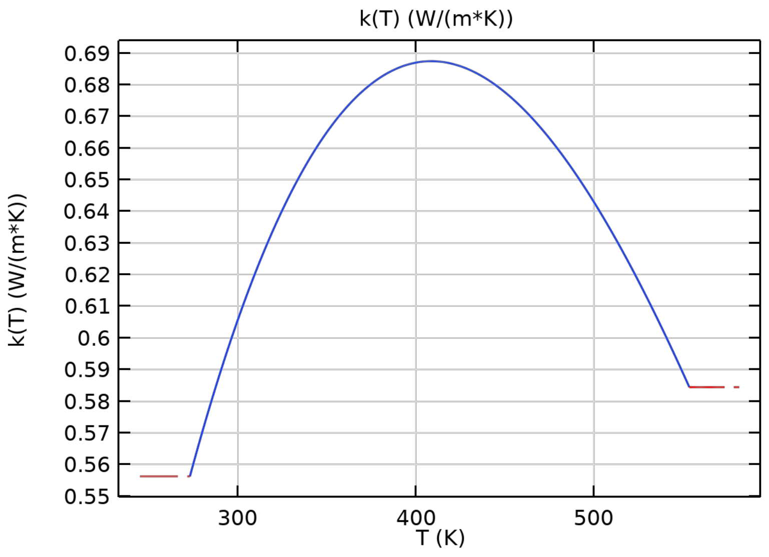

k

Interpolation

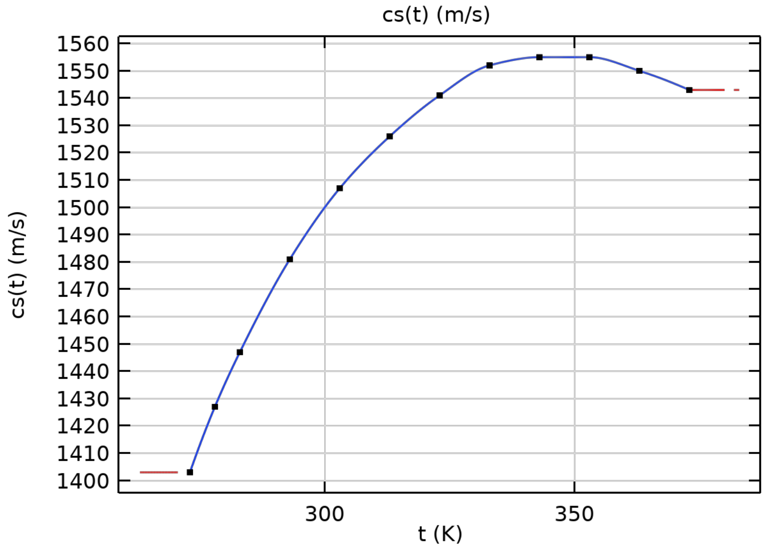

cs

Analytic

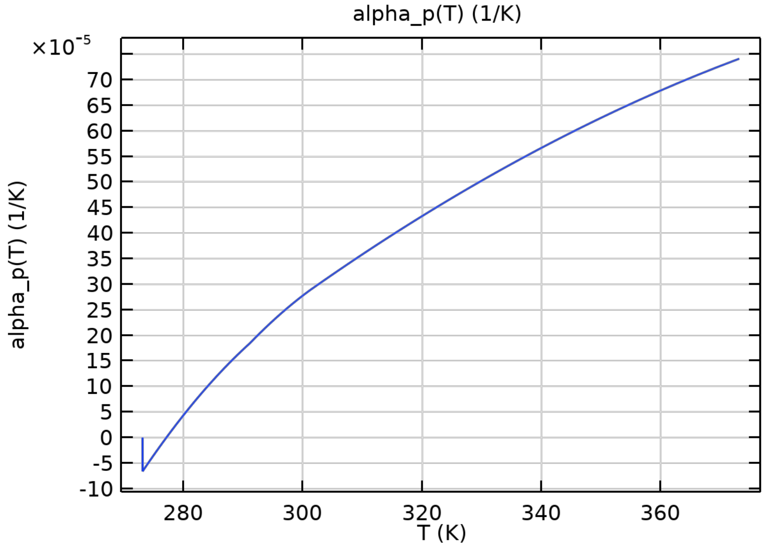

alpha_p

Analytic 2

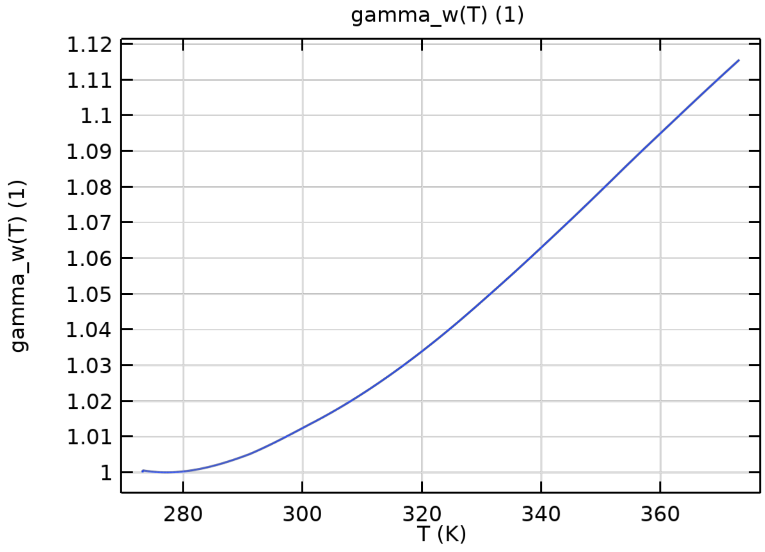

gamma_w

Analytic 3

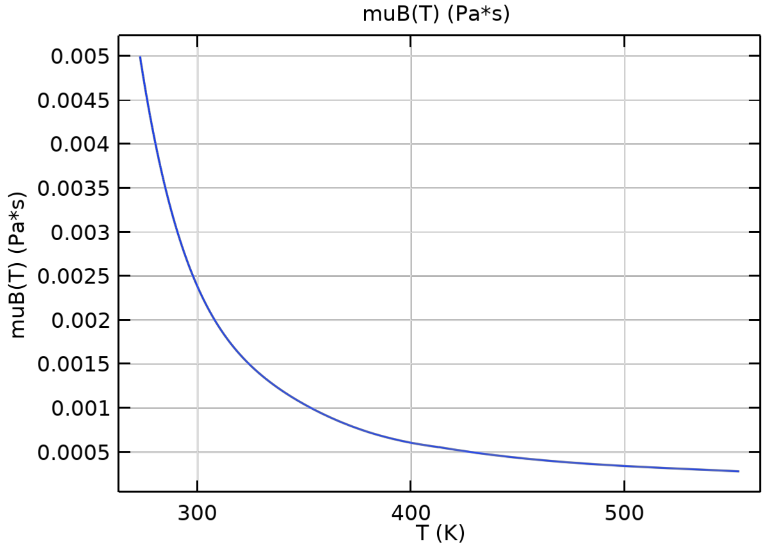

muB

2.4 TRANSPORT OF DILUTED SPECIES

USED PRODUCTS

Chemical Reaction Engineering Module
COMSOL Multiphysics

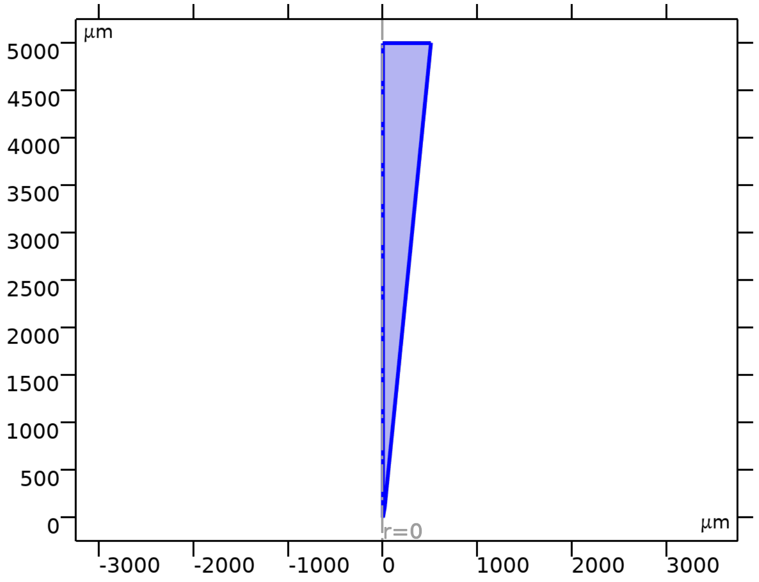

Transport of Diluted Species

SELECTION

Geometric entity level	Domain
Selection	Geometry geom1: Dimension 2: All domains

EQUATIONS

$$\frac{\partial c_i}{\partial t} + \nabla \cdot \mathbf{J}_i = R_i$$
$$\mathbf{J}_i = -D_i \nabla c_i$$

2.4.1 Interface Settings

Discretization

SETTINGS

Description	Value
Concentration	Linear

SETTINGS

Description	Value
Equation form	Study controlled

Species Activity

SETTINGS

Description	Value
Species activity	Ideal

Transport Mechanisms

SETTINGS

Description	Value
Convection	Off
Migration in electric field	Off
Mass transfer in porous media	Off

2.4.2 Variables

Name	Expression	Unit	Description	Selection	Details
tds.d	1	1	Out-of-plane geometry extension	Global	
tds.f_Cox	1	1	Activity coefficient	Domains 1–3	
tds.f_Cred	1	1	Activity coefficient	Domains 1–3	
tds.nr	nr	1	Normal vector, r-component	Boundaries 4, 6	
tds.nphi	0	1	Normal vector, phi-component	Boundaries 4, 6	
tds.nz	nz	1	Normal vector, z-component	Boundaries 4, 6	
tds.nr	dnr	1	Normal vector, r-component	Boundaries 1–3, 5, 7–11	
tds.nphi	0	1	Normal vector, phi-component	Boundaries 1–3, 5, 7–11	
tds.nz	dnz	1	Normal vector, z-component	Boundaries 1–3, 5, 7–11	
tds.nrmesh	nrmesh	1	Normal vector (mesh), r-component	Boundaries 4, 6	
tds.nphimesh	0	1	Normal vector (mesh), phi-component	Boundaries 4, 6	
tds.nzmesh	nzmesh	1	Normal vector (mesh), z-component	Boundaries 4, 6	
tds.nrmesh	dnrmesh	1	Normal vector (mesh), r-component	Boundaries 1–3, 5, 7–11	
tds.nphimesh	0	1	Normal vector (mesh), phi-component	Boundaries 1–3, 5, 7–11	

Name	Expression	Unit	Description	Selection	Details
tds.nzmesh	dnzmesh	1	Normal vector (mesh), z-component	Boundaries 1–3, 5, 7–11	
tds.nrc	root.nrc/tds.ncLen	1	Normal vector, r-component	Boundaries 1–11	
tds.nphic	0	1	Normal vector, phi-component	Boundaries 1–11	
tds.nzc	root.nzc/tds.ncLen	1	Normal vector, z-component	Boundaries 1–11	
tds.ncLen	sqrt(root.nrc^2+root.nzc^2+eps)	1	Help variable	Boundaries 1–11	
tds.R_Cox	0	mol/(m³·s)	Total rate expression	Domains 1–3	+ operation
tds.cP_Cox	0	mol/kg	Concentration species adsorbed to the solid	Domains 1–3	+ operation
tds.cP_Cox	0	mol/kg	Concentration species adsorbed to the solid	Boundaries 1–11	+ operation
tds.KP_Cox	0	m³/kg	Adsorption isotherm, first concentration derivative	Domains 1–3	+ operation
tds.KP_Cox	0	m³/kg	Adsorption isotherm, first concentration derivative	Boundaries 1–11	+ operation
tds.Rads_Cox	0	mol/(m³·s)	Total adsorption rate	Domains 1–3	+ operation
tds.DiT_Cox	0	m²/s	Turbulent diffusivity	Domains 1–3	
tds.cVar_Cox	Cox	mol/m³	Species	Boundaries 1–11	
tds.cVar_Cox	Cox	mol/m³	Species	Points 1–9	
tds.R_Cred	0	mol/(m³·s)	Total rate expression	Domains 1–3	+ operation
tds.cP_Cred	0	mol/kg	Concentration species adsorbed to the solid	Domains 1–3	+ operation
tds.cP_Cred	0	mol/kg	Concentration species adsorbed to the solid	Boundaries 1–11	+ operation

Name	Expression	Unit	Description	Selection	Details
tds.KP_Cred	0	m^3/kg	Adsorption isotherm, first concentration derivative	Domains 1–3	+ operation
tds.KP_Cred	0	m^3/kg	Adsorption isotherm, first concentration derivative	Boundaries 1–11	+ operation
tds.Rads_Cred	0	mol/(m^3·s)	Total adsorption rate	Domains 1–3	+ operation
tds.DiT_Cred	0	m^2/s	Turbulent diffusivity	Domains 1–3	
tds.cVar_Cred	Cred	mol/m^3	Species	Boundaries 1–11	
tds.cVar_Cred	Cred	mol/m^3	Species	Points 1–9	
tds.poro	1	1	Porosity	Domains 1–3	
tds.theta_g	0	1	Gas volume fraction	Domains 1–3	
tds.theta_l	1	1	Liquid volume fraction	Domains 1–3	
tds.theta	tds.poro	1	Mobile fluid volume fraction	Domains 1–3	

2.4.3 Transport Properties 1

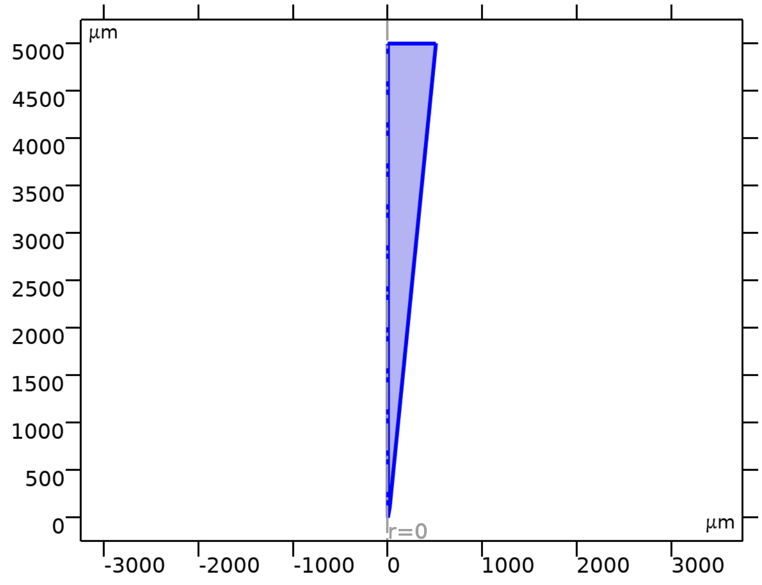

Transport Properties 1

SELECTION

Geometric entity level	Domain
Selection	Geometry geom1: Dimension 2: All domains

EQUATIONS

$$\frac{\partial c_i}{\partial t} + \nabla \cdot \mathbf{J}_i = R_i$$

$$\mathbf{J}_i = -D_i \nabla c_i$$

Diffusion

SETTINGS

Description	Value	Unit
Source	Material	
Material	Water (mat1)	
Diffusion coefficient	User defined	
Diffusion coefficient	Doxi	m^2/s
Diffusion coefficient	User defined	
Diffusion coefficient	Dred	m^2/s

Coordinate System Selection

SETTINGS

Description	Value
Coordinate system	Global coordinate system

Model Input

SETTINGS

Description	Value
Temperature	Common model input

Variables

Name	Expression	Unit	Description	Selection	Details
domflux.Coxr	2*tds.dflux_Coxr*pi*r*tds.d	mol/(m·s)	Domain flux, r-component	Domains 1–3	
domflux.Coxz	2*tds.dflux_Coxz*pi*r*tds.d	mol/(m·s)	Domain flux, z-component	Domains 1–3	
domflux.Credr	2*tds.dflux_Credr*pi*r*tds.d	mol/(m·s)	Domain flux, r-component	Domains 1–3	
domflux.Credz	2*tds.dflux_Credz*pi*r*tds.d	mol/(m·s)	Domain flux, z-component	Domains 1–3	

Name	Expression	Unit	Description	Selection	Details
tds.ndflux_Cox	tds.bndFlux_Cox	mol/(m²·s)	Normal diffusive flux	Boundaries 2, 4, 6–11	
tds.ntflux_Cox	tds.bndFlux_Cox	mol/(m²·s)	Normal total flux	Boundaries 2, 4, 6–11	
tds.ndflux_Cred	tds.bndFlux_Cred	mol/(m²·s)	Normal diffusive flux	Boundaries 2, 4, 6–11	
tds.ntflux_Cred	tds.bndFlux_Cred	mol/(m²·s)	Normal total flux	Boundaries 2, 4, 6–11	
tds.DF_Coxrr	Doxi	m²/s	Fluid diffusion coefficient, rr-component	Domains 1–3	
tds.DF_Coxphir	0	m²/s	Fluid diffusion coefficient, phir-component	Domains 1–3	
tds.DF_Coxzr	0	m²/s	Fluid diffusion coefficient, zr-component	Domains 1–3	
tds.DF_Coxrphi	0	m²/s	Fluid diffusion coefficient, rphi-component	Domains 1–3	
tds.DF_Coxphiphi	Doxi	m²/s	Fluid diffusion coefficient, phiphi-component	Domains 1–3	
tds.DF_Coxzphi	0	m²/s	Fluid diffusion coefficient, zphi-component	Domains 1–3	
tds.DF_Coxrz	0	m²/s	Fluid diffusion coefficient, rz-component	Domains 1–3	
tds.DF_Coxphiz	0	m²/s	Fluid diffusion coefficient, phiz-component	Domains 1–3	
tds.DF_Coxzz	Doxi	m²/s	Fluid diffusion coefficient, zz-component	Domains 1–3	
tds.D_Coxrr	tds.DF_Coxrr+tds.DiT_Cox	m²/s	Diffusion coefficient, rr-component	Domains 1–3	+ operation
tds.D_Coxphir	tds.DF_Coxphir	m²/s	Diffusion coefficient, phir-component	Domains 1–3	+ operation

Name	Expression	Unit	Description	Selection	Details
tds.D_Coxzr	tds.DF_Coxzr	m²/s	Diffusion coefficient, zr-component	Domains 1–3	+ operation
tds.D_Coxrphi	tds.DF_Coxrphi	m²/s	Diffusion coefficient, rphi-component	Domains 1–3	+ operation
tds.D_Coxphiphi	tds.DF_Coxphiphi+tds.DiT_Cox	m²/s	Diffusion coefficient, phiphi-component	Domains 1–3	+ operation
tds.D_Coxzphi	tds.DF_Coxzphi	m²/s	Diffusion coefficient, zphi-component	Domains 1–3	+ operation
tds.D_Coxrz	tds.DF_Coxrz	m²/s	Diffusion coefficient, rz-component	Domains 1–3	+ operation
tds.D_Coxphiz	tds.DF_Coxphiz	m²/s	Diffusion coefficient, phiz-component	Domains 1–3	+ operation
tds.D_Coxzz	tds.DF_Coxzz+tds.DiT_Cox	m²/s	Diffusion coefficient, zz-component	Domains 1–3	+ operation
tds.DF_Credrr	Dred	m²/s	Fluid diffusion coefficient, rr-component	Domains 1–3	
tds.DF_Credphir	0	m²/s	Fluid diffusion coefficient, phir-component	Domains 1–3	
tds.DF_Credzr	0	m²/s	Fluid diffusion coefficient, zr-component	Domains 1–3	
tds.DF_Credrphi	0	m²/s	Fluid diffusion coefficient, rphi-component	Domains 1–3	
tds.DF_Credphiphi	Dred	m²/s	Fluid diffusion coefficient, phiphi-component	Domains 1–3	
tds.DF_Credzphi	0	m²/s	Fluid diffusion coefficient, zphi-component	Domains 1–3	
tds.DF_Credrz	0	m²/s	Fluid diffusion coefficient, rz-	Domains 1–3	

Name	Expression	Unit	Description	Selection	Details
			component		
tds.DF_Credphiz	0	m^2/s	Fluid diffusion coefficient, phiz-component	Domains 1–3	
tds.DF_Credzz	Dred	m^2/s	Fluid diffusion coefficient, zz-component	Domains 1–3	
tds.D_Credrr	tds.DF_Credrr+tds.DiT_Cred	m^2/s	Diffusion coefficient, rr-component	Domains 1–3	+ operation
tds.D_Credphir	tds.DF_Credphir	m^2/s	Diffusion coefficient, phir-component	Domains 1–3	+ operation
tds.D_Credzr	tds.DF_Credzr	m^2/s	Diffusion coefficient, zr-component	Domains 1–3	+ operation
tds.D_Credrphi	tds.DF_Credrphi	m^2/s	Diffusion coefficient, rphi-component	Domains 1–3	+ operation
tds.D_Credphiphi	tds.DF_Credphiphi+tds.DiT_Cred	m^2/s	Diffusion coefficient, phiphi-component	Domains 1–3	+ operation
tds.D_Credzphi	tds.DF_Credzphi	m^2/s	Diffusion coefficient, zphi-component	Domains 1–3	+ operation
tds.D_Credrz	tds.DF_Credrz	m^2/s	Diffusion coefficient, rz-component	Domains 1–3	+ operation
tds.D_Credphiz	tds.DF_Credphiz	m^2/s	Diffusion coefficient, phiz-component	Domains 1–3	+ operation
tds.D_Credzz	tds.DF_Credzz+tds.DiT_Cred	m^2/s	Diffusion coefficient, zz-component	Domains 1–3	+ operation
tds.Dav_Cox	0.5*(tds.D_Coxrr+tds.D_Coxzz)	m^2/s	Average diffusion coefficient	Domains 1–3	
tds.Dav_Cred	0.5*(tds.D_Credrr+tds.D_Credzz)	m^2/s	Average diffusion coefficient	Domains 1–3	
tds.tflux_Coxr	tds.dflux_Coxr	$mol/(m^2 \cdot s)$	Total flux, r-component	Domains 1–3	+ operation
tds.tflux_Coxphi	tds.dflux_Coxphi	$mol/(m^2 \cdot s)$	Total flux, phi-	Domains 1–3	+ operation

Name	Expression	Unit	Description	Selection	Details
			component		
tds.tflux_Coxz	tds.dflux_Coxz	mol/(m²·s)	Total flux, z-component	Domains 1–3	+ operation
tds.dfluxMag_Cox	sqrt(tds.dflux_Coxr^2+tds.dflux_Coxphi^2+tds.dflux_Coxz^2)	mol/(m²·s)	Diffusive flux magnitude	Domains 1–3	
tds.tfluxMag_Cox	sqrt(tds.tflux_Coxr^2+tds.tflux_Coxphi^2+tds.tflux_Coxz^2)	mol/(m²·s)	Total flux magnitude	Domains 1–3	
tds.dpflux_Coxr	0	mol/(m²·s)	Dispersive flux, r-component	Domains 1–3	
tds.dpflux_Coxphi	0	mol/(m²·s)	Dispersive flux, phi-component	Domains 1–3	
tds.dpflux_Coxz	0	mol/(m²·s)	Dispersive flux, z-component	Domains 1–3	
tds.tflux_Credr	tds.dflux_Credr	mol/(m²·s)	Total flux, r-component	Domains 1–3	+ operation
tds.tflux_Credphi	tds.dflux_Credphi	mol/(m²·s)	Total flux, phi-component	Domains 1–3	+ operation
tds.tflux_Credz	tds.dflux_Credz	mol/(m²·s)	Total flux, z-component	Domains 1–3	+ operation
tds.dfluxMag_Cred	sqrt(tds.dflux_Credr^2+tds.dflux_Credphi^2+tds.dflux_Credz^2)	mol/(m²·s)	Diffusive flux magnitude	Domains 1–3	
tds.tfluxMag_Cred	sqrt(tds.tflux_Credr^2+tds.tflux_Credphi^2+tds.tflux_Credz^2)	mol/(m²·s)	Total flux magnitude	Domains 1–3	
tds.dpflux_Credr	0	mol/(m²·s)	Dispersive flux, r-component	Domains 1–3	
tds.dpflux_Credphi	0	mol/(m²·s)	Dispersive flux, phi-component	Domains 1–3	
tds.dpflux_Credz	0	mol/(m²·s)	Dispersive flux, z-component	Domains 1–3	
tds.dflux_Coxr	-tds.D_Coxrr*Coxr-tds.D_Coxrz*Coxz	mol/(m²·s)	Diffusive flux, r-component	Domains 1–3	+ operation
tds.dflux_Coxphi	-tds.D_Coxphir*Coxr-tds.D_Coxphiz*Coxz	mol/(m²·s)	Diffusive flux, phi-component	Domains 1–3	+ operation

Name	Expression	Unit	Description	Selection	Details
tds.dflux_Coxz	-tds.D_Coxzr*Coxr-tds.D_Coxzz*Coxz	mol/(m²·s)	Diffusive flux, z-component	Domains 1–3	+ operation
tds.grad_Coxr	Coxr	mol/m⁴	Concentration gradient, r-component	Domains 1–3	
tds.grad_Coxphi	0	mol/m⁴	Concentration gradient, phi-component	Domains 1–3	
tds.grad_Coxz	Coxz	mol/m⁴	Concentration gradient, z-component	Domains 1–3	
tds.dflux_Credr	-tds.D_Credrr*Credr-tds.D_Credrz*Credz	mol/(m²·s)	Diffusive flux, r-component	Domains 1–3	+ operation
tds.dflux_Credphi	-tds.D_Credphir*Credr-tds.D_Credphiz*Credz	mol/(m²·s)	Diffusive flux, phi-component	Domains 1–3	+ operation
tds.dflux_Credz	-tds.D_Credzr*Credr-tds.D_Credzz*Credz	mol/(m²·s)	Diffusive flux, z-component	Domains 1–3	+ operation
tds.grad_Credr	Credr	mol/m⁴	Concentration gradient, r-component	Domains 1–3	
tds.grad_Credphi	0	mol/m⁴	Concentration gradient, phi-component	Domains 1–3	
tds.grad_Credz	Credz	mol/m⁴	Concentration gradient, z-component	Domains 1–3	
tds.bndFlux_Cox	0.25*(uflux_spatial(Cox)-dflux_spatial(Cox))/(pi*r*tds.d)	mol/(m²·s)	Boundary flux	Boundaries 4, 6	Meta
tds.bndFlux_Cox	-dflux_spatial(Cox)/tds.d	mol/(m²·s)	Boundary flux	Boundaries 1, 3, 5	
tds.bndFlux_Cox	-0.5*dflux_spatial(Cox)/(pi*r*tds.d)	mol/(m²·s)	Boundary flux	Boundaries 2, 7–11	Meta
tds.bndFlux_Cred	0.25*(uflux_spatial(Cred)-	mol/(m²·s)	Boundary flux	Boundaries 4, 6	Meta

Name	Expression	Unit	Description	Selection	Details
	dflux_spatial(Cred))/(pi*r*tds.d)				
tds.bndFlux_Cred	-dflux_spatial(Cred)/tds.d	mol/(m²·s)	Boundary flux	Boundaries 1, 3, 5	
tds.bndFlux_Cred	-0.5*dflux_spatial(Cred)/(pi*r*tds.d)	mol/(m²·s)	Boundary flux	Boundaries 2, 7–11	Meta
tds.Rlin_Cox	0	1/s	Linear source term coefficient	Domains 1–3	+ operation
tds.Res_Cox	d(Cox,t)-Cox*tds.Rlin_Cox-tds.R_Cox	mol/(m³·s)	Equation residual	Domains 1–3	
tds.Rlin_Cred	0	1/s	Linear source term coefficient	Domains 1–3	+ operation
tds.Res_Cred	d(Cred,t)-Cred*tds.Rlin_Cred-tds.R_Cred	mol/(m³·s)	Equation residual	Domains 1–3	

Shape functions

Name	Shape function	Unit	Description	Shape frame	Selection
Cox	Lagrange (Linear)	mol/m³	Concentration	Spatial	Domains 1–3
Cred	Lagrange (Linear)	mol/m³	Concentration	Spatial	Domains 1–3

Weak Expressions

Weak expression	Integration order	Integration frame	Selection
2*(-Coxt*test(Cox)+tds.dflux_Coxr*test(Coxr)+tds.dflux_Coxz*test(Coxz))*tds.d*pi*r	2	Spatial	Domains 1–3
2*(-Credt*test(Cred)+tds.dflux_Credr*test(Credr)+tds.dflux_Credz*test(Credz))*tds.d*pi*r	2	Spatial	Domains 1–3
2*tds.streamline*(isScalingSystemDomain==0)*tds.d*pi*r	2	Spatial	Domains 1–3
2*tds.crosswind*(isScalingSystemDomain==0)*tds.d*pi*r	4	Spatial	Domains 1–3

2.4.4 Axial Symmetry 1

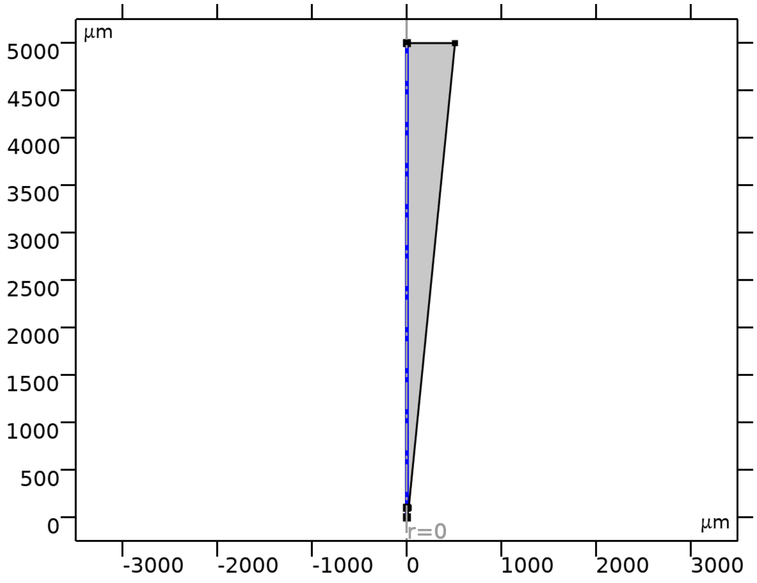

Axial Symmetry 1

SELECTION

Geometric entity level	Boundary
Selection	Geometry geom1: Dimension 1: All boundaries

2.4.5 No Flux 1

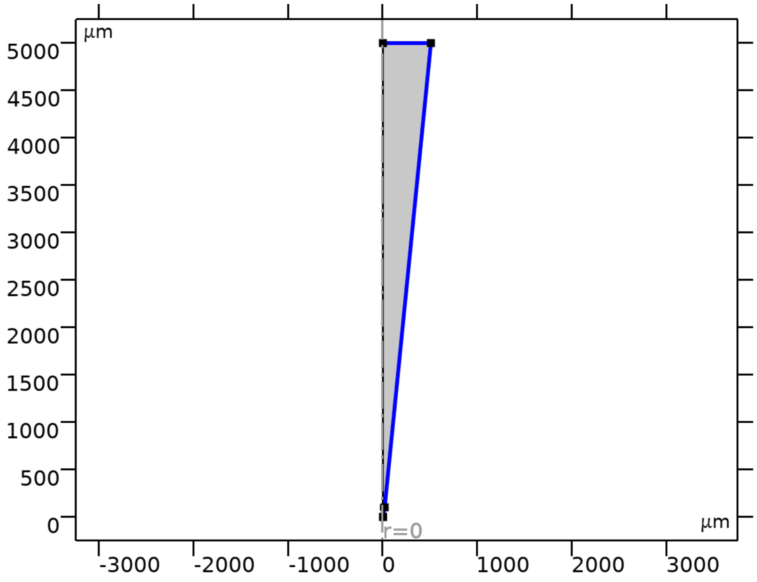

No Flux 1

SELECTION

Geometric entity level	Boundary

Selection	Geometry geom1: Dimension 1: All boundaries

EQUATIONS

$$-\mathbf{n} \cdot \mathbf{J}_i = 0$$

2.4.6 Initial Values 1

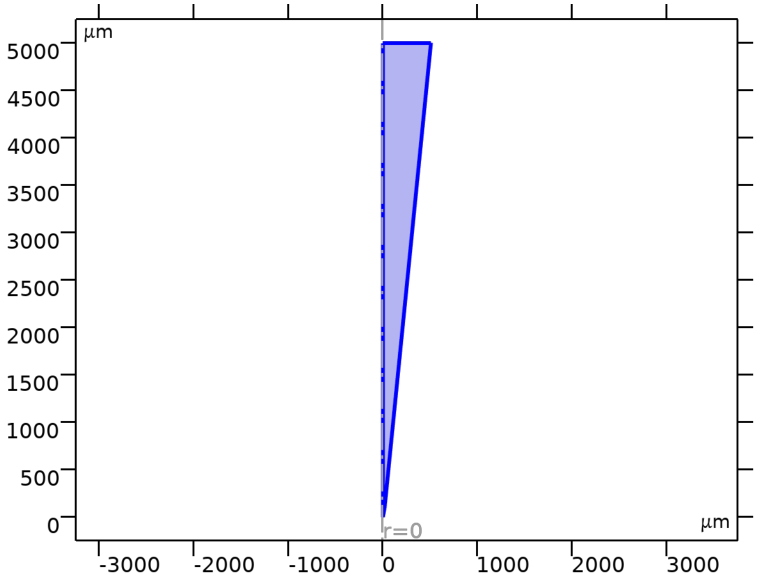

Initial Values 1

SELECTION

Geometric entity level	Domain
Selection	Geometry geom1: Dimension 2: All domains

Initial Values

SETTINGS

Description	Value	Unit
Concentration	{0, C0}	mol/m^3

Variables

Name	Expression	Unit	Description	Selection	Details
tds.c0_Cox	0	mol/m^3	Concentration	Domains 1–3	+ operation
tds.c0_Cred	C0	mol/m^3	Concentration	Domains 1–3	+ operation

2.4.7 Flux - BV option 2

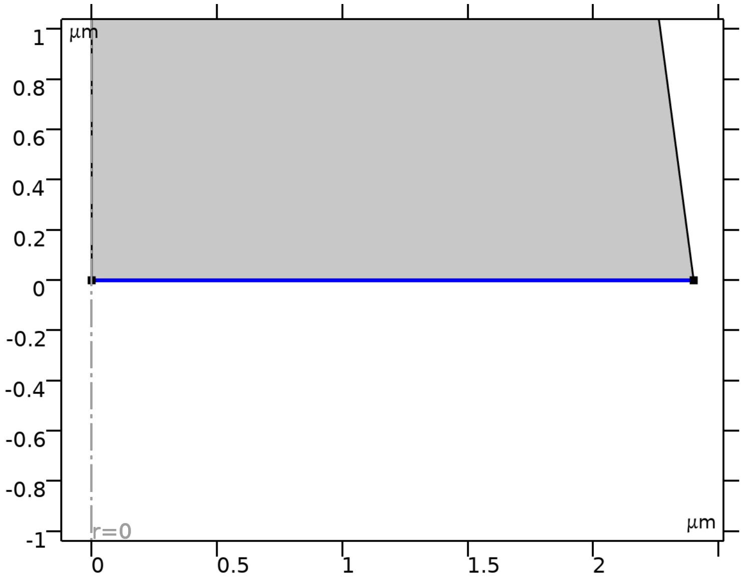

Flux - BV option 2

SELECTION

Geometric entity level	Boundary
Selection	Geometry geom1: Dimension 1: Boundary 2

EQUATIONS

$$-\mathbf{n}\cdot\mathbf{J}_i = J_{0,i}$$

Inward Flux

SETTINGS

Description	Value	Unit
Flux type	General inward flux	
Species Cox	On	
Species Cred	On	
	{-Kred*Cox + Koxi*Cred, Kred*Cox - Koxi*Cred}	mol/(m²·s)

Variables

Name	Expression	Unit	Description	Selection
tds.fl3.nmflow_Cox	tds.fl3.int(2*tds.ntflux_Cox*pi*r)*tds.d	mol/s	Normal molar flow rate	Global
tds.fl3.nmflow_Cred	tds.fl3.int(2*tds.ntflux_Cred*pi*r)*tds.d	mol/s	Normal molar flow rate	Global

Weak Expressions

Weak expression	Integration order	Integration frame	Selection
2*(-Kred*Cox+Koxi*Cred)*test(Cox)*tds.d*pi*r	2	Spatial	Boundary 2
2*(Kred*Cox-Koxi*Cred)*test(Cred)*tds.d*pi*r	2	Spatial	Boundary 2

2.5 MESH 1

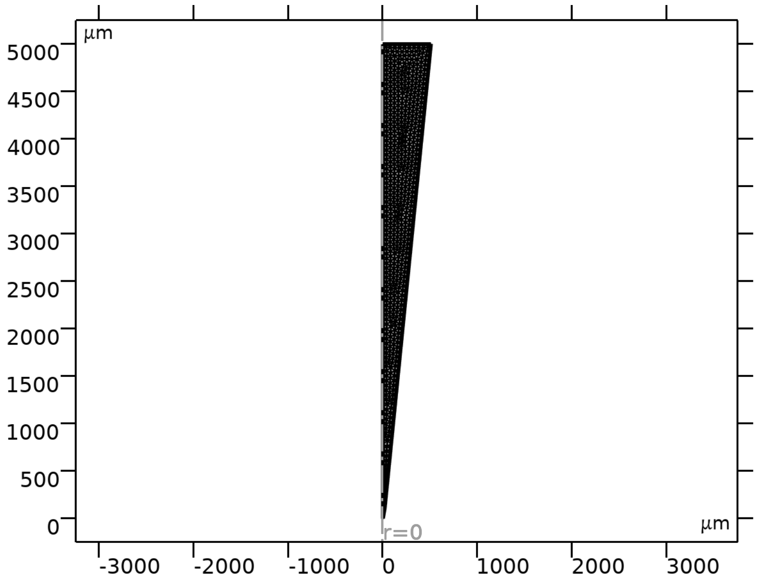

Mesh 1

MESH STATISTICS

Description	Value
Status	Complete mesh
Mesh vertices	975
Triangles	1649
Edge elements	304
Vertex elements	9
Number of elements	1649
Minimum element quality	0.5763
Average element quality	0.9011
Element area ratio	4.4234E-5
Mesh area	1.292E6 μm^2

2.5.1 Size (size)

SETTINGS

Description	Value
Maximum element size	50
Curvature factor	0.2
Predefined size	Extremely fine

2.5.2 Free Triangular 1 (ftri1)

SELECTION

Geometric entity level	Domain
Selection	Remaining

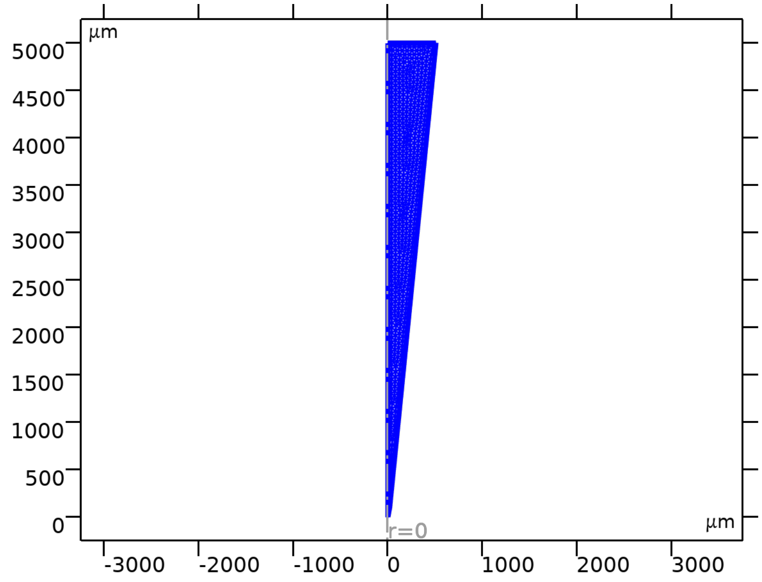

Free Triangular 1

SETTINGS

Description	Value
Number of iterations	4
Maximum element depth to process	4
Last build time	0
Built with	COMSOL 6.1.0.252 (win64)|2025 - 03 - 30T16:08:55.317793100

3 Study 1

COMPUTATION INFORMATION

Computation time	13 s

3.1 SECCM

Times	Unit
range(0.0001,0.0001,0.001) range(0.001,0.001,1.0001) range(1,1,120) range(120,10,300)	s

STUDY SETTINGS

Description	Value
Include geometric nonlinearity	Off

STUDY SETTINGS

Description	Value
Output times	{1E-4, 2E-4, 3E-4, 4E-4, 5E-4, 6E-4, 7E-4, 8E-4, 9E-4, 0.001, 0.001, 0.002, 0.003, 0.004, 0.005, 0.006, 0.007, 0.008, 0.009, 0.01, 0.011, 0.012, 0.013, 0.014, 0.015, 0.016, 0.017, 0.018, 0.019, 0.02, 0.021, 0.022, 0.023, 0.024, 0.025, 0.026, 0.027, 0.028, 0.029, 0.03, 0.031, 0.032, 0.033, 0.034, 0.035, 0.036, 0.037, 0.038, 0.039, 0.04, 0.041, 0.042, 0.043, 0.044, 0.045, 0.046, 0.047, 0.048, 0.049, 0.05, 0.051, 0.052, 0.053, 0.054, 0.055, 0.056, 0.057, 0.058, 0.059, 0.06, 0.061, 0.062, 0.063, 0.064, 0.065, 0.066, 0.067, 0.068, 0.069, 0.07, 0.071, 0.072, 0.073, 0.074, 0.075, 0.076, 0.077, 0.078, 0.079, 0.08, 0.081, 0.082, 0.083, 0.084, 0.085, 0.086, 0.087, 0.088, 0.089, 0.09, 0.091, 0.092, 0.093, 0.094, 0.095, 0.096, 0.097, 0.098, 0.099, 0.1, 0.101, 0.102, 0.103, 0.104, 0.105, 0.106, 0.107, 0.108, 0.109, 0.11, 0.111, 0.112, 0.113, 0.114, 0.115, 0.116, 0.117, 0.118, 0.119, 0.12, 0.121, 0.122, 0.123, 0.124, 0.125, 0.126, 0.127, 0.128, 0.129, 0.13, 0.131, 0.132, 0.133, 0.134, 0.135, 0.136, 0.137, 0.138, 0.139, 0.14, 0.141, 0.142, 0.143, 0.144, 0.145, 0.146, 0.147, 0.148, 0.149, 0.15, 0.151, 0.152, 0.153, 0.154, 0.155, 0.156, 0.157, 0.158, 0.159, 0.16, 0.161, 0.162, 0.163, 0.164, 0.165, 0.166, 0.167, 0.168, 0.169, 0.17, 0.171, 0.172, 0.173, 0.174, 0.175, 0.176, 0.177, 0.178, 0.179, 0.18, 0.181, 0.182, 0.183, 0.184, 0.185, 0.186, 0.187, 0.188, 0.189, 0.19, 0.191, 0.192, 0.193, 0.194, 0.195, 0.196, 0.197, 0.198, 0.199, 0.2, 0.201, 0.202, 0.203, 0.204, 0.205, 0.206, 0.207, 0.208, 0.209, 0.21, 0.211, 0.212, 0.213, 0.214, 0.215, 0.216, 0.217, 0.218, 0.219, 0.22, 0.221, 0.222, 0.223, 0.224, 0.225, 0.226, 0.227, 0.228, 0.229, 0.23, 0.231, 0.232, 0.233, 0.234, 0.235, 0.236, 0.237, 0.238, 0.239, 0.24, 0.241, 0.242, 0.243, 0.244, 0.245, 0.246, 0.247, 0.248, 0.249, 0.25, 0.251, 0.252, 0.253, 0.254, 0.255, 0.256, 0.257, 0.258, 0.259, 0.26, 0.261, 0.262, 0.263, 0.264, 0.265, 0.266, 0.267, 0.268, 0.269, 0.27, 0.271, 0.272, 0.273, 0.274, 0.275, 0.276, 0.277, 0.278, 0.279, 0.28, 0.281, 0.282, 0.283, 0.284, 0.285, 0.286, 0.287, 0.288, 0.289, 0.29, 0.291, 0.292, 0.293, 0.294, 0.295, 0.296, 0.297, 0.298, 0.299, 0.3, 0.301, 0.302, 0.303, 0.304, 0.305, 0.306, 0.307, 0.308, 0.309, 0.31, 0.311, 0.312, 0.313, 0.314, 0.315, 0.316, 0.317, 0.318, 0.319, 0.32, 0.321, 0.322, 0.323, 0.324, 0.325, 0.326, 0.327, 0.328, 0.329, 0.33, 0.331, 0.332, 0.333, 0.334, 0.335, 0.336, 0.337, 0.338, 0.339, 0.34, 0.341, 0.342, 0.343, 0.344, 0.345, 0.346, 0.347, 0.348, 0.349, 0.35, 0.351, 0.352, 0.353, 0.354, 0.355, 0.356, 0.357, 0.358, 0.359, 0.36, 0.361, 0.362, 0.363, 0.364, 0.365, 0.366, 0.367, 0.368, 0.369, 0.37, 0.371, 0.372, 0.373, 0.374, 0.375, 0.376, 0.377, 0.378, 0.379, 0.38, 0.381, 0.382, 0.383, 0.384, 0.385, 0.386, 0.387, 0.388, 0.389, 0.39, 0.391, 0.392, 0.393, 0.394, 0.395,

Description	Value
	0.396, 0.397, 0.398, 0.399, 0.4, 0.401, 0.402, 0.403, 0.404, 0.405, 0.406, 0.407, 0.408, 0.409, 0.41, 0.411, 0.412, 0.413, 0.414, 0.415, 0.416, 0.417, 0.418, 0.419, 0.42, 0.421, 0.422, 0.423, 0.424, 0.425, 0.426, 0.427, 0.428, 0.429, 0.43, 0.431, 0.432, 0.433, 0.434, 0.435, 0.436, 0.437, 0.438, 0.439, 0.44, 0.441, 0.442, 0.443, 0.444, 0.445, 0.446, 0.447, 0.448, 0.449, 0.45, 0.451, 0.452, 0.453, 0.454, 0.455, 0.456, 0.457, 0.458, 0.459, 0.46, 0.461, 0.462, 0.463, 0.464, 0.465, 0.466, 0.467, 0.468, 0.469, 0.47, 0.471, 0.472, 0.473, 0.474, 0.475, 0.476, 0.477, 0.478, 0.479, 0.48, 0.481, 0.482, 0.483, 0.484, 0.485, 0.486, 0.487, 0.488, 0.489, 0.49, 0.491, 0.492, 0.493, 0.494, 0.495, 0.496, 0.497, 0.498, 0.499, 0.5, 0.501, 0.502, 0.503, 0.504, 0.505, 0.506, 0.507, 0.508, 0.509, 0.51, 0.511, 0.512, 0.513, 0.514, 0.515, 0.516, 0.517, 0.518, 0.519, 0.52, 0.521, 0.522, 0.523, 0.524, 0.525, 0.526, 0.527, 0.528, 0.529, 0.53, 0.531, 0.532, 0.533, 0.534, 0.535, 0.536, 0.537, 0.538, 0.539, 0.54, 0.541, 0.542, 0.543, 0.544, 0.545, 0.546, 0.547, 0.548, 0.549, 0.55, 0.551, 0.552, 0.553, 0.554, 0.555, 0.556, 0.557, 0.558, 0.559, 0.56, 0.561, 0.562, 0.563, 0.564, 0.565, 0.566, 0.567, 0.568, 0.569, 0.57, 0.571, 0.572, 0.573, 0.574, 0.575, 0.576, 0.577, 0.578, 0.579, 0.58, 0.581, 0.582, 0.583, 0.584, 0.585, 0.586, 0.587, 0.588, 0.589, 0.59, 0.591, 0.592, 0.593, 0.594, 0.595, 0.596, 0.597, 0.598, 0.599, 0.6, 0.601, 0.602, 0.603, 0.604, 0.605, 0.606, 0.607, 0.608, 0.609, 0.61, 0.611, 0.612, 0.613, 0.614, 0.615, 0.616, 0.617, 0.618, 0.619, 0.62, 0.621, 0.622, 0.623, 0.624, 0.625, 0.626, 0.627, 0.628, 0.629, 0.63, 0.631, 0.632, 0.633, 0.634, 0.635, 0.636, 0.637, 0.638, 0.639, 0.64, 0.641, 0.642, 0.643, 0.644, 0.645, 0.646, 0.647, 0.648, 0.649, 0.65, 0.651, 0.652, 0.653, 0.654, 0.655, 0.656, 0.657, 0.658, 0.659, 0.66, 0.661, 0.662, 0.663, 0.664, 0.665, 0.666, 0.667, 0.668, 0.669, 0.67, 0.671, 0.672, 0.673, 0.674, 0.675, 0.676, 0.677, 0.678, 0.679, 0.68, 0.681, 0.682, 0.683, 0.684, 0.685, 0.686, 0.687, 0.688, 0.689, 0.69, 0.691, 0.692, 0.693, 0.694, 0.695, 0.696, 0.697, 0.698, 0.699, 0.7, 0.701, 0.702, 0.703, 0.704, 0.705, 0.706, 0.707, 0.708, 0.709, 0.71, 0.711, 0.712, 0.713, 0.714, 0.715, 0.716, 0.717, 0.718, 0.719, 0.72, 0.721, 0.722, 0.723, 0.724, 0.725, 0.726, 0.727, 0.728, 0.729, 0.73, 0.731, 0.732, 0.733, 0.734, 0.735, 0.736, 0.737, 0.738, 0.739, 0.74, 0.741, 0.742, 0.743, 0.744, 0.745, 0.746, 0.747, 0.748, 0.749, 0.75, 0.751, 0.752, 0.753, 0.754, 0.755, 0.756, 0.757, 0.758, 0.759, 0.76, 0.761, 0.762, 0.763, 0.764, 0.765, 0.766, 0.767, 0.768, 0.769, 0.77, 0.771, 0.772, 0.773, 0.774, 0.775, 0.776, 0.777, 0.778, 0.779, 0.78, 0.781, 0.782, 0.783, 0.784, 0.785, 0.786, 0.787, 0.788, 0.789, 0.79, 0.791, 0.792, 0.793, 0.794, 0.795, 0.796, 0.797, 0.798, 0.799, 0.8, 0.801, 0.802, 0.803, 0.804, 0.805, 0.806, 0.807, 0.808, 0.809, 0.81, 0.811, 0.812, 0.813, 0.814, 0.815, 0.816, 0.817, 0.818, 0.819, 0.82, 0.821, 0.822, 0.823, 0.824, 0.825, 0.826, 0.827, 0.828, 0.829, 0.83, 0.831, 0.832, 0.833, 0.834, 0.835, 0.836, 0.837, 0.838, 0.839, 0.84, 0.841, 0.842, 0.843, 0.844, 0.845, 0.846, 0.847, 0.848, 0.849, 0.85, 0.851, 0.852, 0.853, 0.854, 0.855, 0.856, 0.857, 0.858, 0.859, 0.86, 0.861, 0.862, 0.863, 0.864, 0.865, 0.866, 0.867, 0.868, 0.869, 0.87, 0.871, 0.872, 0.873, 0.874, 0.875, 0.876, 0.877, 0.878, 0.879, 0.88, 0.881, 0.882, 0.883, 0.884, 0.885, 0.886, 0.887, 0.888, 0.889, 0.89, 0.891, 0.892, 0.893, 0.894, 0.895, 0.896, 0.897, 0.898, 0.899, 0.9, 0.901, 0.902, 0.903, 0.904, 0.905, 0.906, 0.907, 0.908, 0.909, 0.91, 0.911, 0.912, 0.913, 0.914, 0.915, 0.916, 0.917, 0.918, 0.919, 0.92, 0.921, 0.922, 0.923, 0.924, 0.925, 0.926, 0.927, 0.928, 0.929, 0.93, 0.931, 0.932, 0.933, 0.934, 0.935, 0.936, 0.937, 0.938, 0.939, 0.94, 0.941, 0.942, 0.943, 0.944, 0.945, 0.946, 0.947, 0.948, 0.949, 0.95, 0.951, 0.952, 0.953, 0.954, 0.955, 0.956, 0.957, 0.958, 0.959, 0.96, 0.961, 0.962, 0.963, 0.964, 0.965, 0.966, 0.967, 0.968, 0.969, 0.97, 0.971, 0.972, 0.973, 0.974, 0.975, 0.976, 0.977, 0.978, 0.979, 0.98, 0.981, 0.982, 0.983, 0.984, 0.985, 0.986, 0.987, 0.988, 0.989, 0.99, 0.991, 0.992, 0.993, 0.994, 0.995, 0.996, 0.997, 0.998, 0.999, 1, 1, 2, 3, 4, 5, 6, 7, 8, 9, 10, 11, 12, 13, 14, 15, 16, 17, 18, 19, 20, 21, 22, 23, 24, 25, 26, 27, 28, 29, 30, 31, 32, 33, 34, 35, 36, 37, 38, 39, 40, 41, 42, 43,

Description	Value
	44, 45, 46, 47, 48, 49, 50, 51, 52, 53, 54, 55, 56, 57, 58, 59, 60, 61, 62, 63, 64, 65, 66, 67, 68, 69, 70, 71, 72, 73, 74, 75, 76, 77, 78, 79, 80, 81, 82, 83, 84, 85, 86, 87, 88, 89, 90, 91, 92, 93, 94, 95, 96, 97, 98, 99, 100, 101, 102, 103, 104, 105, 106, 107, 108, 109, 110, 111, 112, 113, 114, 115, 116, 117, 118, 119, 120, 120, 130, 140, 150, 160, 170, 180, 190, 200, 210, 220, 230, 240, 250, 260, 270, 280, 290, 300}

PHYSICS AND VARIABLES SELECTION

Physics interface	Discretization
Transport of Diluted Species (tds)	physics

MESH SELECTION

Geometry	Mesh
Geometry 1 (geom1)	mesh1

3.2 PIPETTE TRANSFER

Times	Unit
range(300,0.001,301), range(301,1,390)	s

STUDY SETTINGS

Description	Value
Include geometric nonlinearity	Off

STUDY SETTINGS

Description	Value
Output times	{300, 300, 300, 300, 300, 300.01, 300.01, 300.01, 300.01, 300.01, 300.01, 300.01, 300.01, 300.01, 300.01, 300.02, 300.02, 300.02, 300.02, 300.02, 300.02, 300.02, 300.02, 300.02, 300.02, 300.03, 300.03, 300.03, 300.03, 300.03, 300.03, 300.03, 300.03, 300.03, 300.03, 300.04, 300.04, 300.04, 300.04, 300.04, 300.04, 300.04, 300.04, 300.04, 300.04, 300.05, 300.05, 300.05, 300.05, 300.05, 300.05, 300.05, 300.05, 300.05, 300.05, 300.06, 300.06, 300.06, 300.06, 300.06, 300.06, 300.06, 300.06, 300.06, 300.06, 300.07, 300.07, 300.07, 300.07, 300.07, 300.07, 300.07, 300.07, 300.07, 300.07, 300.08, 300.08, 300.08, 300.08, 300.08, 300.08, 300.08, 300.08, 300.08, 300.08, 300.09, 300.09, 300.09, 300.09, 300.09, 300.09, 300.09, 300.09, 300.09, 300.09, 300.1, 300.1, 300.1, 300.1, 300.1, 300.1, 300.1, 300.1, 300.1, 300.1, 300.11, 300.11, 300.11, 300.11, 300.11, 300.11, 300.11, 300.11, 300.11, 300.11, 300.12, 300.12, 300.12, 300.12, 300.12, 300.12, 300.12, 300.12, 300.12, 300.12, 300.13, 300.13, 300.13, 300.13, 300.13, 300.13, 300.13, 300.13, 300.13, 300.13, 300.14, 300.14, 300.14, 300.14, 300.14, 300.14, 300.14, 300.14, 300.14, 300.14, 300.15, 300.15, 300.15, 300.15, 300.15, 300.15, 300.15, 300.15, 300.15, 300.15, 300.16, 300.16, 300.16, 300.16, 300.16, 300.16, 300.16, 300.16, 300.16, 300.16, 300.17, 300.17, 300.17, 300.17, 300.17, 300.17, 300.17, 300.17, 300.17, 300.17, 300.18, 300.18, 300.18, 300.18, 300.18, 300.18, 300.18, 300.18, 300.18, 300.18, 300.19, 300.19, 300.19, 300.19, 300.19, 300.19, 300.19, 300.19, 300.19, 300.19, 300.2, 300.2, 300.2, 300.2, 300.2, 300.2, 300.2, 300.2, 300.2, 300.2, 300.21, 300.21, 300.21, 300.21, 300.21, 300.21, 300.21, 300.21,

Description	Value
	300.21, 300.21, 300.22, 300.22, 300.22, 300.22, 300.22, 300.22, 300.22, 300.22, 300.22, 300.22, 300.23, 300.23, 300.23, 300.23, 300.23, 300.23, 300.23, 300.23, 300.23, 300.23, 300.24, 300.24, 300.24, 300.24, 300.24, 300.24, 300.24, 300.24, 300.24, 300.24, 300.25, 300.25, 300.25, 300.25, 300.25, 300.25, 300.25, 300.25, 300.25, 300.25, 300.26, 300.26, 300.26, 300.26, 300.26, 300.26, 300.26, 300.26, 300.26, 300.26, 300.27, 300.27, 300.27, 300.27, 300.27, 300.27, 300.27, 300.27, 300.27, 300.27, 300.28, 300.28, 300.28, 300.28, 300.28, 300.28, 300.28, 300.28, 300.28, 300.28, 300.29, 300.29, 300.29, 300.29, 300.29, 300.29, 300.29, 300.29, 300.29, 300.29, 300.3, 300.3, 300.3, 300.3, 300.3, 300.3, 300.3, 300.3, 300.3, 300.3, 300.31, 300.31, 300.31, 300.31, 300.31, 300.31, 300.31, 300.31, 300.31, 300.31, 300.32, 300.32, 300.32, 300.32, 300.32, 300.32, 300.32, 300.32, 300.32, 300.32, 300.33, 300.33, 300.33, 300.33, 300.33, 300.33, 300.33, 300.33, 300.33, 300.33, 300.34, 300.34, 300.34, 300.34, 300.34, 300.34, 300.34, 300.34, 300.34, 300.34, 300.35, 300.35, 300.35, 300.35, 300.35, 300.35, 300.35, 300.35, 300.35, 300.35, 300.36, 300.36, 300.36, 300.36, 300.36, 300.36, 300.36, 300.36, 300.36, 300.36, 300.37, 300.37, 300.37, 300.37, 300.37, 300.37, 300.37, 300.37, 300.37, 300.37, 300.38, 300.38, 300.38, 300.38, 300.38, 300.38, 300.38, 300.38, 300.38, 300.38, 300.39, 300.39, 300.39, 300.39, 300.39, 300.39, 300.39, 300.39, 300.39, 300.39, 300.4, 300.4, 300.4, 300.4, 300.4, 300.4, 300.4, 300.4, 300.4, 300.4, 300.41, 300.41, 300.41, 300.41, 300.41, 300.41, 300.41, 300.41, 300.41, 300.41, 300.42, 300.42, 300.42, 300.42, 300.42, 300.42, 300.42, 300.42, 300.42, 300.42, 300.43, 300.43, 300.43, 300.43, 300.43, 300.43, 300.43, 300.43, 300.43, 300.43, 300.44, 300.44, 300.44, 300.44, 300.44, 300.44, 300.44, 300.44, 300.44, 300.44, 300.45, 300.45, 300.45, 300.45, 300.45, 300.45, 300.45, 300.45, 300.45, 300.45, 300.46, 300.46, 300.46, 300.46, 300.46, 300.46, 300.46, 300.46, 300.46, 300.46, 300.47, 300.47, 300.47, 300.47, 300.47, 300.47, 300.47, 300.47, 300.47, 300.47, 300.48, 300.48, 300.48, 300.48, 300.48, 300.48, 300.48, 300.48, 300.48, 300.48, 300.49, 300.49, 300.49, 300.49, 300.49, 300.49, 300.49, 300.49, 300.49, 300.49, 300.5, 300.5, 300.5, 300.5, 300.5, 300.5, 300.5, 300.5, 300.5, 300.5, 300.51, 300.51, 300.51, 300.51, 300.51, 300.51, 300.51, 300.51, 300.51, 300.51, 300.52, 300.52, 300.52, 300.52, 300.52, 300.52, 300.52, 300.52, 300.52, 300.52, 300.53, 300.53, 300.53, 300.53, 300.53, 300.53, 300.53, 300.53, 300.53, 300.53, 300.54, 300.54, 300.54, 300.54, 300.54, 300.54, 300.54, 300.54, 300.54, 300.54, 300.55, 300.55, 300.55, 300.55, 300.55, 300.55, 300.55, 300.55, 300.55, 300.55, 300.56, 300.56, 300.56, 300.56, 300.56, 300.56, 300.56, 300.56, 300.56, 300.56, 300.57, 300.57, 300.57, 300.57, 300.57, 300.57, 300.57, 300.57, 300.57, 300.57, 300.58, 300.58, 300.58, 300.58, 300.58, 300.58, 300.58, 300.58, 300.58, 300.58, 300.59, 300.59, 300.59, 300.59, 300.59, 300.59, 300.59, 300.59, 300.59, 300.59, 300.6, 300.6, 300.6, 300.6, 300.6, 300.6, 300.6, 300.6, 300.6, 300.6, 300.61, 300.61, 300.61, 300.61, 300.61, 300.61, 300.61, 300.61, 300.61, 300.61, 300.62, 300.62, 300.62, 300.62, 300.62, 300.62, 300.62, 300.62, 300.62, 300.62, 300.63, 300.63, 300.63, 300.63, 300.63, 300.63, 300.63, 300.63, 300.63, 300.63, 300.64, 300.64, 300.64, 300.64, 300.64, 300.64, 300.64, 300.64, 300.64, 300.64, 300.65, 300.65, 300.65, 300.65, 300.65, 300.65, 300.65, 300.65, 300.65, 300.65, 300.66, 300.66, 300.66, 300.66, 300.66, 300.66, 300.66, 300.66, 300.66, 300.66, 300.67, 300.67, 300.67, 300.67, 300.67, 300.67, 300.67, 300.67, 300.67, 300.67, 300.68, 300.68, 300.68, 300.68, 300.68, 300.68, 300.68, 300.68, 300.68, 300.68, 300.69, 300.69, 300.69, 300.69, 300.69, 300.69, 300.69, 300.69, 300.69, 300.69, 300.7, 300.7, 300.7, 300.7, 300.7, 300.7, 300.7, 300.7, 300.7, 300.7, 300.71, 300.71, 300.71, 300.71, 300.71, 300.71, 300.71, 300.71, 300.71, 300.71, 300.72, 300.72, 300.72, 300.72, 300.72, 300.72, 300.72, 300.72, 300.72, 300.72, 300.73, 300.73, 300.73, 300.73, 300.73, 300.73, 300.73, 300.73, 300.73, 300.73, 300.74, 300.74, 300.74, 300.74, 300.74, 300.74, 300.74, 300.74, 300.74, 300.74, 300.75,

Description	Value
	300.75, 300.75, 300.75, 300.75, 300.75, 300.75, 300.75, 300.75, 300.75, 300.76, 300.76, 300.76, 300.76, 300.76, 300.76, 300.76, 300.76, 300.76, 300.76, 300.77, 300.77, 300.77, 300.77, 300.77, 300.77, 300.77, 300.77, 300.77, 300.77, 300.78, 300.78, 300.78, 300.78, 300.78, 300.78, 300.78, 300.78, 300.78, 300.78, 300.79, 300.79, 300.79, 300.79, 300.79, 300.79, 300.79, 300.79, 300.79, 300.79, 300.8, 300.8, 300.8, 300.8, 300.8, 300.8, 300.8, 300.8, 300.8, 300.8, 300.81, 300.81, 300.81, 300.81, 300.81, 300.81, 300.81, 300.81, 300.81, 300.81, 300.82, 300.82, 300.82, 300.82, 300.82, 300.82, 300.82, 300.82, 300.82, 300.82, 300.83, 300.83, 300.83, 300.83, 300.83, 300.83, 300.83, 300.83, 300.83, 300.83, 300.84, 300.84, 300.84, 300.84, 300.84, 300.84, 300.84, 300.84, 300.84, 300.84, 300.85, 300.85, 300.85, 300.85, 300.85, 300.85, 300.85, 300.85, 300.85, 300.85, 300.86, 300.86, 300.86, 300.86, 300.86, 300.86, 300.86, 300.86, 300.86, 300.86, 300.87, 300.87, 300.87, 300.87, 300.87, 300.87, 300.87, 300.87, 300.87, 300.87, 300.88, 300.88, 300.88, 300.88, 300.88, 300.88, 300.88, 300.88, 300.88, 300.88, 300.89, 300.89, 300.89, 300.89, 300.89, 300.89, 300.89, 300.89, 300.89, 300.89, 300.9, 300.9, 300.9, 300.9, 300.9, 300.9, 300.9, 300.9, 300.9, 300.9, 300.91, 300.91, 300.91, 300.91, 300.91, 300.91, 300.91, 300.91, 300.91, 300.91, 300.92, 300.92, 300.92, 300.92, 300.92, 300.92, 300.92, 300.92, 300.92, 300.92, 300.93, 300.93, 300.93, 300.93, 300.93, 300.93, 300.93, 300.93, 300.93, 300.93, 300.94, 300.94, 300.94, 300.94, 300.94, 300.94, 300.94, 300.94, 300.94, 300.94, 300.95, 300.95, 300.95, 300.95, 300.95, 300.95, 300.95, 300.95, 300.95, 300.95, 300.96, 300.96, 300.96, 300.96, 300.96, 300.96, 300.96, 300.96, 300.96, 300.96, 300.97, 300.97, 300.97, 300.97, 300.97, 300.97, 300.97, 300.97, 300.97, 300.97, 300.98, 300.98, 300.98, 300.98, 300.98, 300.98, 300.98, 300.98, 300.98, 300.98, 300.99, 300.99, 300.99, 300.99, 300.99, 300.99, 300.99, 300.99, 300.99, 300.99, 301, 301, 301, 301, 301, 301, 301, 302, 303, 304, 305, 306, 307, 308, 309, 310, 311, 312, 313, 314, 315, 316, 317, 318, 319, 320, 321, 322, 323, 324, 325, 326, 327, 328, 329, 330, 331, 332, 333, 334, 335, 336, 337, 338, 339, 340, 341, 342, 343, 344, 345, 346, 347, 348, 349, 350, 351, 352, 353, 354, 355, 356, 357, 358, 359, 360, 361, 362, 363, 364, 365, 366, 367, 368, 369, 370, 371, 372, 373, 374, 375, 376, 377, 378, 379, 380, 381, 382, 383, 384, 385, 386, 387, 388, 389, 390}

VALUES OF DEPENDENT VARIABLES

Description	Value
Settings	For selections
Selections	{Lower level 2, Lower pipet_level 1}

PHYSICS AND VARIABLES SELECTION

Physics interface	Discretization
Transport of Diluted Species (tds)	physics

MESH SELECTION

Geometry	Mesh
Geometry 1 (geom1)	mesh1

3.3 SOLVER CONFIGURATIONS

3.3.1 Solution 1

Compile Equations: SECCM (st1)

STUDY AND STEP

Description	Value
Use study	Study 1
Use study step	SECCM

LOG

```
<---- Compile Equations: SECCM in Study 1/Solution 1 (sol1) --------------------
Started at Mar 30, 2025, 4:08:54 PM.
Running on Intel(R) Core(TM) i7-10750H CPU at 2.60 GHz.
Using 1 socket with 6 cores in total on AYNN.
Available memory: 16.25 GB.
Geometry shape function: Linear Lagrange
Number of vertex elements: 9
Number of boundary elements: 304
Number of elements: 1649
Minimum element quality: 0.5763
Time: 1 s.
Physical memory: 1.5 GB
Virtual memory: 1.86 GB
Ended at Mar 30, 2025, 4:08:56 PM.
----- Compile Equations: SECCM in Study 1/Solution 1 (sol1) ------------------->
```

Dependent Variables 1 (v1)

GENERAL

Description	Value
Defined by study step	SECCM

RESIDUAL SCALING

Description	Value
Method	Manual

INITIAL VALUE CALCULATION CONSTANTS

Constant name	Initial value source
t	range(0.0001,0.0001,0.001) range(0.001,0.001,1.0001) range(1,1,120) range(120,10,300)
timestep	0.3[s]
timestep	0.09[s]

LOG

```
<---- Dependent Variables 1 in Study 1/Solution 1 (sol1) -----------------------
Started at Mar 30, 2025, 4:08:56 PM.
Solution time: 0 s.
Physical memory: 1.43 GB
Virtual memory: 1.81 GB
Ended at Mar 30, 2025, 4:08:56 PM.
----- Dependent Variables 1 in Study 1/Solution 1 (sol1) ----------------------->
```

Concentration (comp1.Cox) (comp1_Cox)

GENERAL

Description	Value
Field components	comp1.Cox
Internal variables	{comp1.uflux.Cox, comp1.dflux.Cox, comp1.tds.dt2Inv_Cox}

Concentration (comp1.Cred) (comp1_Cred)

GENERAL

Description	Value
Field components	comp1.Cred
Internal variables	{comp1.uflux.Cred, comp1.dflux.Cred, comp1.tds.dt2Inv_Cred}

Time-Dependent Solver 1 (t1)

GENERAL

Description	Value
Defined by study step	SECCM
Output times	{1E-4, 2E-4, 3E-4, 4E-4, 5E-4, 6E-4, 7E-4, 8E-4, 9E-4, 0.001, 0.001, 0.002, 0.003, 0.004, 0.005, 0.006, 0.007, 0.008, 0.009, 0.01, 0.011, 0.012, 0.013, 0.014, 0.015, 0.016, 0.017, 0.018, 0.019, 0.02, 0.021, 0.022, 0.023, 0.024, 0.025, 0.026, 0.027, 0.028, 0.029, 0.03, 0.031, 0.032, 0.033, 0.034, 0.035, 0.036, 0.037, 0.038, 0.039, 0.04, 0.041, 0.042, 0.043, 0.044, 0.045, 0.046, 0.047, 0.048, 0.049, 0.05, 0.051, 0.052, 0.053, 0.054, 0.055, 0.056, 0.057, 0.058, 0.059, 0.06, 0.061, 0.062, 0.063, 0.064, 0.065, 0.066, 0.067, 0.068, 0.069, 0.07, 0.071, 0.072, 0.073, 0.074, 0.075, 0.076, 0.077, 0.078, 0.079, 0.08, 0.081, 0.082, 0.083, 0.084, 0.085, 0.086, 0.087, 0.088, 0.089, 0.09, 0.091, 0.092, 0.093, 0.094, 0.095, 0.096, 0.097, 0.098, 0.099, 0.1, 0.101, 0.102, 0.103, 0.104, 0.105, 0.106, 0.107, 0.108, 0.109, 0.11, 0.111, 0.112, 0.113, 0.114, 0.115, 0.116, 0.117, 0.118, 0.119, 0.12, 0.121, 0.122, 0.123, 0.124, 0.125, 0.126, 0.127, 0.128, 0.129, 0.13, 0.131, 0.132, 0.133, 0.134, 0.135, 0.136, 0.137, 0.138, 0.139, 0.14, 0.141, 0.142, 0.143, 0.144, 0.145, 0.146, 0.147, 0.148, 0.149, 0.15, 0.151, 0.152, 0.153, 0.154, 0.155, 0.156, 0.157, 0.158, 0.159, 0.16, 0.161, 0.162, 0.163, 0.164, 0.165, 0.166, 0.167, 0.168, 0.169, 0.17, 0.171, 0.172, 0.173, 0.174, 0.175, 0.176, 0.177, 0.178, 0.179, 0.18, 0.181, 0.182, 0.183, 0.184, 0.185, 0.186, 0.187, 0.188, 0.189, 0.19, 0.191, 0.192, 0.193, 0.194, 0.195, 0.196, 0.197, 0.198, 0.199, 0.2, 0.201, 0.202, 0.203, 0.204, 0.205, 0.206, 0.207, 0.208, 0.209, 0.21, 0.211, 0.212, 0.213, 0.214, 0.215, 0.216, 0.217, 0.218, 0.219, 0.22, 0.221, 0.222, 0.223, 0.224, 0.225, 0.226,

Description	Value
	0.227, 0.228, 0.229, 0.23, 0.231, 0.232, 0.233, 0.234, 0.235, 0.236, 0.237,<br>0.238, 0.239, 0.24, 0.241, 0.242, 0.243, 0.244, 0.245, 0.246, 0.247, 0.248,<br>0.249, 0.25, 0.251, 0.252, 0.253, 0.254, 0.255, 0.256, 0.257, 0.258, 0.259, 0.26,<br>0.261, 0.262, 0.263, 0.264, 0.265, 0.266, 0.267, 0.268, 0.269, 0.27, 0.271,<br>0.272, 0.273, 0.274, 0.275, 0.276, 0.277, 0.278, 0.279, 0.28, 0.281, 0.282,<br>0.283, 0.284, 0.285, 0.286, 0.287, 0.288, 0.289, 0.29, 0.291, 0.292, 0.293,<br>0.294, 0.295, 0.296, 0.297, 0.298, 0.299, 0.3, 0.301, 0.302, 0.303, 0.304, 0.305,<br>0.306, 0.307, 0.308, 0.309, 0.31, 0.311, 0.312, 0.313, 0.314, 0.315, 0.316,<br>0.317, 0.318, 0.319, 0.32, 0.321, 0.322, 0.323, 0.324, 0.325, 0.326, 0.327,<br>0.328, 0.329, 0.33, 0.331, 0.332, 0.333, 0.334, 0.335, 0.336, 0.337, 0.338,<br>0.339, 0.34, 0.341, 0.342, 0.343, 0.344, 0.345, 0.346, 0.347, 0.348, 0.349, 0.35,<br>0.351, 0.352, 0.353, 0.354, 0.355, 0.356, 0.357, 0.358, 0.359, 0.36, 0.361,<br>0.362, 0.363, 0.364, 0.365, 0.366, 0.367, 0.368, 0.369, 0.37, 0.371, 0.372,<br>0.373, 0.374, 0.375, 0.376, 0.377, 0.378, 0.379, 0.38, 0.381, 0.382, 0.383,<br>0.384, 0.385, 0.386, 0.387, 0.388, 0.389, 0.39, 0.391, 0.392, 0.393, 0.394,<br>0.395, 0.396, 0.397, 0.398, 0.399, 0.4, 0.401, 0.402, 0.403, 0.404, 0.405, 0.406,<br>0.407, 0.408, 0.409, 0.41, 0.411, 0.412, 0.413, 0.414, 0.415, 0.416, 0.417,<br>0.418, 0.419, 0.42, 0.421, 0.422, 0.423, 0.424, 0.425, 0.426, 0.427, 0.428,<br>0.429, 0.43, 0.431, 0.432, 0.433, 0.434, 0.435, 0.436, 0.437, 0.438, 0.439, 0.44,<br>0.441, 0.442, 0.443, 0.444, 0.445, 0.446, 0.447, 0.448, 0.449, 0.45, 0.451,<br>0.452, 0.453, 0.454, 0.455, 0.456, 0.457, 0.458, 0.459, 0.46, 0.461, 0.462,<br>0.463, 0.464, 0.465, 0.466, 0.467, 0.468, 0.469, 0.47, 0.471, 0.472, 0.473,<br>0.474, 0.475, 0.476, 0.477, 0.478, 0.479, 0.48, 0.481, 0.482, 0.483, 0.484,<br>0.485, 0.486, 0.487, 0.488, 0.489, 0.49, 0.491, 0.492, 0.493, 0.494, 0.495,<br>0.496, 0.497, 0.498, 0.499, 0.5, 0.501, 0.502, 0.503, 0.504, 0.505, 0.506, 0.507,<br>0.508, 0.509, 0.51, 0.511, 0.512, 0.513, 0.514, 0.515, 0.516, 0.517, 0.518,<br>0.519, 0.52, 0.521, 0.522, 0.523, 0.524, 0.525, 0.526, 0.527, 0.528, 0.529, 0.53,<br>0.531, 0.532, 0.533, 0.534, 0.535, 0.536, 0.537, 0.538, 0.539, 0.54, 0.541,<br>0.542, 0.543, 0.544, 0.545, 0.546, 0.547, 0.548, 0.549, 0.55, 0.551, 0.552,<br>0.553, 0.554, 0.555, 0.556, 0.557, 0.558, 0.559, 0.56, 0.561, 0.562, 0.563,<br>0.564, 0.565, 0.566, 0.567, 0.568, 0.569, 0.57, 0.571, 0.572, 0.573, 0.574,<br>0.575, 0.576, 0.577, 0.578, 0.579, 0.58, 0.581, 0.582, 0.583, 0.584, 0.585,<br>0.586, 0.587, 0.588, 0.589, 0.59, 0.591, 0.592, 0.593, 0.594, 0.595, 0.596,<br>0.597, 0.598, 0.599, 0.6, 0.601, 0.602, 0.603, 0.604, 0.605, 0.606, 0.607, 0.608,<br>0.609, 0.61, 0.611, 0.612, 0.613, 0.614, 0.615, 0.616, 0.617, 0.618, 0.619, 0.62,<br>0.621, 0.622, 0.623, 0.624, 0.625, 0.626, 0.627, 0.628, 0.629, 0.63, 0.631,<br>0.632, 0.633, 0.634, 0.635, 0.636, 0.637, 0.638, 0.639, 0.64, 0.641, 0.642,<br>0.643, 0.644, 0.645, 0.646, 0.647, 0.648, 0.649, 0.65, 0.651, 0.652, 0.653,<br>0.654, 0.655, 0.656, 0.657, 0.658, 0.659, 0.66, 0.661, 0.662, 0.663, 0.664,<br>0.665, 0.666, 0.667, 0.668, 0.669, 0.67, 0.671, 0.672, 0.673, 0.674, 0.675,<br>0.676, 0.677, 0.678, 0.679, 0.68, 0.681, 0.682, 0.683, 0.684, 0.685, 0.686,<br>0.687, 0.688, 0.689, 0.69, 0.691, 0.692, 0.693, 0.694, 0.695, 0.696, 0.697,<br>0.698, 0.699, 0.7, 0.701, 0.702, 0.703, 0.704, 0.705, 0.706, 0.707, 0.708, 0.709,<br>0.71, 0.711, 0.712, 0.713, 0.714, 0.715, 0.716, 0.717, 0.718, 0.719, 0.72, 0.721,<br>0.722, 0.723, 0.724, 0.725, 0.726, 0.727, 0.728, 0.729, 0.73, 0.731, 0.732,<br>0.733, 0.734, 0.735, 0.736, 0.737, 0.738, 0.739, 0.74, 0.741, 0.742, 0.743,<br>0.744, 0.745, 0.746, 0.747, 0.748, 0.749, 0.75, 0.751, 0.752, 0.753, 0.754,<br>0.755, 0.756, 0.757, 0.758, 0.759, 0.76, 0.761, 0.762, 0.763, 0.764, 0.765,

Description	Value
	0.766, 0.767, 0.768, 0.769, 0.77, 0.771, 0.772, 0.773, 0.774, 0.775, 0.776, 0.777, 0.778, 0.779, 0.78, 0.781, 0.782, 0.783, 0.784, 0.785, 0.786, 0.787, 0.788, 0.789, 0.79, 0.791, 0.792, 0.793, 0.794, 0.795, 0.796, 0.797, 0.798, 0.799, 0.8, 0.801, 0.802, 0.803, 0.804, 0.805, 0.806, 0.807, 0.808, 0.809, 0.81, 0.811, 0.812, 0.813, 0.814, 0.815, 0.816, 0.817, 0.818, 0.819, 0.82, 0.821, 0.822, 0.823, 0.824, 0.825, 0.826, 0.827, 0.828, 0.829, 0.83, 0.831, 0.832, 0.833, 0.834, 0.835, 0.836, 0.837, 0.838, 0.839, 0.84, 0.841, 0.842, 0.843, 0.844, 0.845, 0.846, 0.847, 0.848, 0.849, 0.85, 0.851, 0.852, 0.853, 0.854, 0.855, 0.856, 0.857, 0.858, 0.859, 0.86, 0.861, 0.862, 0.863, 0.864, 0.865, 0.866, 0.867, 0.868, 0.869, 0.87, 0.871, 0.872, 0.873, 0.874, 0.875, 0.876, 0.877, 0.878, 0.879, 0.88, 0.881, 0.882, 0.883, 0.884, 0.885, 0.886, 0.887, 0.888, 0.889, 0.89, 0.891, 0.892, 0.893, 0.894, 0.895, 0.896, 0.897, 0.898, 0.899, 0.9, 0.901, 0.902, 0.903, 0.904, 0.905, 0.906, 0.907, 0.908, 0.909, 0.91, 0.911, 0.912, 0.913, 0.914, 0.915, 0.916, 0.917, 0.918, 0.919, 0.92, 0.921, 0.922, 0.923, 0.924, 0.925, 0.926, 0.927, 0.928, 0.929, 0.93, 0.931, 0.932, 0.933, 0.934, 0.935, 0.936, 0.937, 0.938, 0.939, 0.94, 0.941, 0.942, 0.943, 0.944, 0.945, 0.946, 0.947, 0.948, 0.949, 0.95, 0.951, 0.952, 0.953, 0.954, 0.955, 0.956, 0.957, 0.958, 0.959, 0.96, 0.961, 0.962, 0.963, 0.964, 0.965, 0.966, 0.967, 0.968, 0.969, 0.97, 0.971, 0.972, 0.973, 0.974, 0.975, 0.976, 0.977, 0.978, 0.979, 0.98, 0.981, 0.982, 0.983, 0.984, 0.985, 0.986, 0.987, 0.988, 0.989, 0.99, 0.991, 0.992, 0.993, 0.994, 0.995, 0.996, 0.997, 0.998, 0.999, 1, 1, 2, 3, 4, 5, 6, 7, 8, 9, 10, 11, 12, 13, 14, 15, 16, 17, 18, 19, 20, 21, 22, 23, 24, 25, 26, 27, 28, 29, 30, 31, 32, 33, 34, 35, 36, 37, 38, 39, 40, 41, 42, 43, 44, 45, 46, 47, 48, 49, 50, 51, 52, 53, 54, 55, 56, 57, 58, 59, 60, 61, 62, 63, 64, 65, 66, 67, 68, 69, 70, 71, 72, 73, 74, 75, 76, 77, 78, 79, 80, 81, 82, 83, 84, 85, 86, 87, 88, 89, 90, 91, 92, 93, 94, 95, 96, 97, 98, 99, 100, 101, 102, 103, 104, 105, 106, 107, 108, 109, 110, 111, 112, 113, 114, 115, 116, 117, 118, 119, 120, 120, 130, 140, 150, 160, 170, 180, 190, 200, 210, 220, 230, 240, 250, 260, 270, 280, 290, 300}
Relative tolerance	0.005

TIME STEPPING

Description	Value
Maximum BDF order	2
Nonlinear controller	On

LOG

-	0.32	-	out
-	0.321	-	out
-	0.322	-	out
-	0.323	-	out
-	0.324	-	out
-	0.325	-	out
-	0.326	-	out
-	0.327	-	out
-	0.328	-	out
-	0.329	-	out
-	0.33	-	out
-	0.331	-	out
-	0.332	-	out
-	0.333	-	out
-	0.334	-	out
-	0.335	-	out
-	0.336	-	out
-	0.337	-	out
-	0.338	-	out
-	0.339	-	out
-	0.34	-	out
-	0.341	-	out
-	0.342	-	out
-	0.343	-	out
-	0.344	-	out
-	0.345	-	out
-	0.346	-	out
-	0.347	-	out
-	0.348	-	out
-	0.349	-	out
-	0.35	-	out
-	0.351	-	out
-	0.352	-	out
-	0.353	-	out
-	0.354	-	out
-	0.355	-	out
-	0.356	-	out
-	0.357	-	out
-	0.358	-	out
-	0.359	-	out
-	0.36	-	out
-	0.361	-	out
-	0.362	-	out
-	0.363	-	out
-	0.364	-	out
-	0.365	-	out
-	0.366	-	out
-	0.367	-	out
-	0.368	-	out
-	0.369	-	out
-	0.37	-	out
-	0.371	-	out
-	0.372	-	out
-	0.373	-	out
-	0.374	-	out
-	0.375	-	out

-	0.376	- out								
-	0.377	- out								
-	0.378	- out								
-	0.379	- out								
-	0.38	- out								
-	0.381	- out								
-	0.382	- out								
-	0.383	- out								
-	0.384	- out								
-	0.385	- out								
-	0.386	- out								
-	0.387	- out								
-	0.388	- out								
-	0.389	- out								
-	0.39	- out								
-	0.391	- out								
-	0.392	- out								
-	0.393	- out								
39	0.39374	0.10133	84	42	84	2	0	0	3e-15	9.5e-15
-	0.394	- out								
-	0.395	- out								
-	0.396	- out								
-	0.397	- out								
-	0.398	- out								
-	0.399	- out								
-	0.4	- out								
-	0.401	- out								
-	0.402	- out								
-	0.403	- out								
-	0.404	- out								
-	0.405	- out								
-	0.406	- out								
-	0.407	- out								
-	0.408	- out								
-	0.409	- out								
-	0.41	- out								
-	0.411	- out								
-	0.412	- out								
-	0.413	- out								
-	0.414	- out								
-	0.415	- out								
-	0.416	- out								
-	0.417	- out								
-	0.418	- out								
-	0.419	- out								
-	0.42	- out								
-	0.421	- out								
-	0.422	- out								
-	0.423	- out								
-	0.424	- out								
-	0.425	- out								
-	0.426	- out								
-	0.427	- out								
-	0.428	- out								
-	0.429	- out								
-	0.43	- out								

-	0.431	-	out
-	0.432	-	out
-	0.433	-	out
-	0.434	-	out
-	0.435	-	out
-	0.436	-	out
-	0.437	-	out
-	0.438	-	out
-	0.439	-	out
-	0.44	-	out
-	0.441	-	out
-	0.442	-	out
-	0.443	-	out
-	0.444	-	out
-	0.445	-	out
-	0.446	-	out
-	0.447	-	out
-	0.448	-	out
-	0.449	-	out
-	0.45	-	out
-	0.451	-	out
-	0.452	-	out
-	0.453	-	out
-	0.454	-	out
-	0.455	-	out
-	0.456	-	out
-	0.457	-	out
-	0.458	-	out
-	0.459	-	out
-	0.46	-	out
-	0.461	-	out
-	0.462	-	out
-	0.463	-	out
-	0.464	-	out
-	0.465	-	out
-	0.466	-	out
-	0.467	-	out
-	0.468	-	out
-	0.469	-	out
-	0.47	-	out
-	0.471	-	out
-	0.472	-	out
-	0.473	-	out
-	0.474	-	out
-	0.475	-	out
-	0.476	-	out
-	0.477	-	out
-	0.478	-	out
-	0.479	-	out
-	0.48	-	out
-	0.481	-	out
-	0.482	-	out
-	0.483	-	out
-	0.484	-	out
-	0.485	-	out
-	0.486	-	out

-	0.487	-	out							
-	0.488	-	out							
-	0.489	-	out							
-	0.49	-	out							
-	0.491	-	out							
-	0.492	-	out							
-	0.493	-	out							
-	0.494	-	out							
-	0.495	-	out							
40	0.49507	0.10133	86	43	86	2	0	0	1.9e-15	8.7e-15
-	0.496	-	out							
-	0.497	-	out							
-	0.498	-	out							
-	0.499	-	out							
-	0.5	-	out							
-	0.501	-	out							
-	0.502	-	out							
-	0.503	-	out							
-	0.504	-	out							
-	0.505	-	out							
-	0.506	-	out							
-	0.507	-	out							
-	0.508	-	out							
-	0.509	-	out							
-	0.51	-	out							
-	0.511	-	out							
-	0.512	-	out							
-	0.513	-	out							
-	0.514	-	out							
-	0.515	-	out							
-	0.516	-	out							
-	0.517	-	out							
-	0.518	-	out							
-	0.519	-	out							
-	0.52	-	out							
-	0.521	-	out							
-	0.522	-	out							
-	0.523	-	out							
-	0.524	-	out							
-	0.525	-	out							
-	0.526	-	out							
-	0.527	-	out							
-	0.528	-	out							
-	0.529	-	out							
-	0.53	-	out							
-	0.531	-	out							
-	0.532	-	out							
-	0.533	-	out							
-	0.534	-	out							
-	0.535	-	out							
-	0.536	-	out							
-	0.537	-	out							
-	0.538	-	out							
-	0.539	-	out							
-	0.54	-	out							
-	0.541	-	out							

-	0.542	-	out							
-	0.543	-	out							
-	0.544	-	out							
-	0.545	-	out							
-	0.546	-	out							
-	0.547	-	out							
-	0.548	-	out							
-	0.549	-	out							
-	0.55	-	out							
-	0.551	-	out							
-	0.552	-	out							
-	0.553	-	out							
-	0.554	-	out							
-	0.555	-	out							
-	0.556	-	out							
-	0.557	-	out							
-	0.558	-	out							
-	0.559	-	out							
-	0.56	-	out							
-	0.561	-	out							
-	0.562	-	out							
-	0.563	-	out							
-	0.564	-	out							
-	0.565	-	out							
-	0.566	-	out							
-	0.567	-	out							
-	0.568	-	out							
-	0.569	-	out							
-	0.57	-	out							
-	0.571	-	out							
-	0.572	-	out							
-	0.573	-	out							
-	0.574	-	out							
-	0.575	-	out							
-	0.576	-	out							
-	0.577	-	out							
-	0.578	-	out							
-	0.579	-	out							
-	0.58	-	out							
-	0.581	-	out							
-	0.582	-	out							
-	0.583	-	out							
-	0.584	-	out							
-	0.585	-	out							
-	0.586	-	out							
-	0.587	-	out							
-	0.588	-	out							
-	0.589	-	out							
-	0.59	-	out							
-	0.591	-	out							
-	0.592	-	out							
-	0.593	-	out							
-	0.594	-	out							
-	0.595	-	out							
-	0.596	-	out							
41	0.5964	0.10133	88	44	88	2	0	0	2.3e-15	9.3e-15

-	0.597	- out
-	0.598	- out
-	0.599	- out
-	0.6	- out
-	0.601	- out
-	0.602	- out
-	0.603	- out
-	0.604	- out
-	0.605	- out
-	0.606	- out
-	0.607	- out
-	0.608	- out
-	0.609	- out
-	0.61	- out
-	0.611	- out
-	0.612	- out
-	0.613	- out
-	0.614	- out
-	0.615	- out
-	0.616	- out
-	0.617	- out
-	0.618	- out
-	0.619	- out
-	0.62	- out
-	0.621	- out
-	0.622	- out
-	0.623	- out
-	0.624	- out
-	0.625	- out
-	0.626	- out
-	0.627	- out
-	0.628	- out
-	0.629	- out
-	0.63	- out
-	0.631	- out
-	0.632	- out
-	0.633	- out
-	0.634	- out
-	0.635	- out
-	0.636	- out
-	0.637	- out
-	0.638	- out
-	0.639	- out
-	0.64	- out
-	0.641	- out
-	0.642	- out
-	0.643	- out
-	0.644	- out
-	0.645	- out
-	0.646	- out
-	0.647	- out
-	0.648	- out
-	0.649	- out
-	0.65	- out
-	0.651	- out
-	0.652	- out

-	0.653	-	out							
-	0.654	-	out							
-	0.655	-	out							
-	0.656	-	out							
-	0.657	-	out							
-	0.658	-	out							
-	0.659	-	out							
-	0.66	-	out							
-	0.661	-	out							
-	0.662	-	out							
-	0.663	-	out							
-	0.664	-	out							
-	0.665	-	out							
-	0.666	-	out							
-	0.667	-	out							
-	0.668	-	out							
-	0.669	-	out							
-	0.67	-	out							
-	0.671	-	out							
-	0.672	-	out							
-	0.673	-	out							
-	0.674	-	out							
-	0.675	-	out							
-	0.676	-	out							
-	0.677	-	out							
-	0.678	-	out							
-	0.679	-	out							
-	0.68	-	out							
-	0.681	-	out							
-	0.682	-	out							
-	0.683	-	out							
-	0.684	-	out							
-	0.685	-	out							
-	0.686	-	out							
-	0.687	-	out							
-	0.688	-	out							
-	0.689	-	out							
-	0.69	-	out							
-	0.691	-	out							
-	0.692	-	out							
-	0.693	-	out							
-	0.694	-	out							
-	0.695	-	out							
-	0.696	-	out							
-	0.697	-	out							
42	0.69773	0.10133	90	45	90	2	0	0	2e-15	8.4e-15
-	0.698	-	out							
-	0.699	-	out							
-	0.7	-	out							
-	0.701	-	out							
-	0.702	-	out							
-	0.703	-	out							
-	0.704	-	out							
-	0.705	-	out							
-	0.706	-	out							
-	0.707	-	out							

-	0.708	-	out
-	0.709	-	out
-	0.71	-	out
-	0.711	-	out
-	0.712	-	out
-	0.713	-	out
-	0.714	-	out
-	0.715	-	out
-	0.716	-	out
-	0.717	-	out
-	0.718	-	out
-	0.719	-	out
-	0.72	-	out
-	0.721	-	out
-	0.722	-	out
-	0.723	-	out
-	0.724	-	out
-	0.725	-	out
-	0.726	-	out
-	0.727	-	out
-	0.728	-	out
-	0.729	-	out
-	0.73	-	out
-	0.731	-	out
-	0.732	-	out
-	0.733	-	out
-	0.734	-	out
-	0.735	-	out
-	0.736	-	out
-	0.737	-	out
-	0.738	-	out
-	0.739	-	out
-	0.74	-	out
-	0.741	-	out
-	0.742	-	out
-	0.743	-	out
-	0.744	-	out
-	0.745	-	out
-	0.746	-	out
-	0.747	-	out
-	0.748	-	out
-	0.749	-	out
-	0.75	-	out
-	0.751	-	out
-	0.752	-	out
-	0.753	-	out
-	0.754	-	out
-	0.755	-	out
-	0.756	-	out
-	0.757	-	out
-	0.758	-	out
-	0.759	-	out
-	0.76	-	out
-	0.761	-	out
-	0.762	-	out
-	0.763	-	out

-	0.764	- out
-	0.765	- out
-	0.766	- out
-	0.767	- out
-	0.768	- out
-	0.769	- out
-	0.77	- out
-	0.771	- out
-	0.772	- out
-	0.773	- out
-	0.774	- out
-	0.775	- out
-	0.776	- out
-	0.777	- out
-	0.778	- out
-	0.779	- out
-	0.78	- out
-	0.781	- out
-	0.782	- out
-	0.783	- out
-	0.784	- out
-	0.785	- out
-	0.786	- out
-	0.787	- out
-	0.788	- out
-	0.789	- out
-	0.79	- out
-	0.791	- out
-	0.792	- out
-	0.793	- out
-	0.794	- out
-	0.795	- out
-	0.796	- out
-	0.797	- out
-	0.798	- out
-	0.799	- out
-	0.8	- out
-	0.801	- out
-	0.802	- out
-	0.803	- out
-	0.804	- out
-	0.805	- out
-	0.806	- out
-	0.807	- out
-	0.808	- out
-	0.809	- out
-	0.81	- out
-	0.811	- out
-	0.812	- out
-	0.813	- out
-	0.814	- out
-	0.815	- out
-	0.816	- out
-	0.817	- out
-	0.818	- out
-	0.819	- out

-	0.82	- out
-	0.821	- out
-	0.822	- out
-	0.823	- out
-	0.824	- out
-	0.825	- out
-	0.826	- out
-	0.827	- out
-	0.828	- out
-	0.829	- out
-	0.83	- out
-	0.831	- out
-	0.832	- out
-	0.833	- out
-	0.834	- out
-	0.835	- out
-	0.836	- out
-	0.837	- out
-	0.838	- out
-	0.839	- out
-	0.84	- out
-	0.841	- out
-	0.842	- out
-	0.843	- out
-	0.844	- out
-	0.845	- out
-	0.846	- out
-	0.847	- out
-	0.848	- out
-	0.849	- out
-	0.85	- out
-	0.851	- out
-	0.852	- out
-	0.853	- out
-	0.854	- out
-	0.855	- out
-	0.856	- out
-	0.857	- out
-	0.858	- out
-	0.859	- out
-	0.86	- out
-	0.861	- out
-	0.862	- out
-	0.863	- out
-	0.864	- out
-	0.865	- out
-	0.866	- out
-	0.867	- out
-	0.868	- out
-	0.869	- out
-	0.87	- out
-	0.871	- out
-	0.872	- out
-	0.873	- out
-	0.874	- out
-	0.875	- out

-	0.876	-	out							
-	0.877	-	out							
-	0.878	-	out							
-	0.879	-	out							
-	0.88	-	out							
-	0.881	-	out							
-	0.882	-	out							
-	0.883	-	out							
-	0.884	-	out							
-	0.885	-	out							
-	0.886	-	out							
-	0.887	-	out							
-	0.888	-	out							
-	0.889	-	out							
-	0.89	-	out							
-	0.891	-	out							
-	0.892	-	out							
-	0.893	-	out							
-	0.894	-	out							
-	0.895	-	out							
-	0.896	-	out							
-	0.897	-	out							
-	0.898	-	out							
-	0.899	-	out							
-	0.9	-	out							
43	0.90039	0.20266	92	46	92	2	0	0	4.8e-15	1.2e-14
-	0.901	-	out							
-	0.902	-	out							
-	0.903	-	out							
-	0.904	-	out							
-	0.905	-	out							
-	0.906	-	out							
-	0.907	-	out							
-	0.908	-	out							
-	0.909	-	out							
-	0.91	-	out							
-	0.911	-	out							
-	0.912	-	out							
-	0.913	-	out							
-	0.914	-	out							
-	0.915	-	out							
-	0.916	-	out							
-	0.917	-	out							
-	0.918	-	out							
-	0.919	-	out							
-	0.92	-	out							
-	0.921	-	out							
-	0.922	-	out							
-	0.923	-	out							
-	0.924	-	out							
-	0.925	-	out							
-	0.926	-	out							
-	0.927	-	out							
-	0.928	-	out							
-	0.929	-	out							
-	0.93	-	out							

-	0.931	- out
-	0.932	- out
-	0.933	- out
-	0.934	- out
-	0.935	- out
-	0.936	- out
-	0.937	- out
-	0.938	- out
-	0.939	- out
-	0.94	- out
-	0.941	- out
-	0.942	- out
-	0.943	- out
-	0.944	- out
-	0.945	- out
-	0.946	- out
-	0.947	- out
-	0.948	- out
-	0.949	- out
-	0.95	- out
-	0.951	- out
-	0.952	- out
-	0.953	- out
-	0.954	- out
-	0.955	- out
-	0.956	- out
-	0.957	- out
-	0.958	- out
-	0.959	- out
-	0.96	- out
-	0.961	- out
-	0.962	- out
-	0.963	- out
-	0.964	- out
-	0.965	- out
-	0.966	- out
-	0.967	- out
-	0.968	- out
-	0.969	- out
-	0.97	- out
-	0.971	- out
-	0.972	- out
-	0.973	- out
-	0.974	- out
-	0.975	- out
-	0.976	- out
-	0.977	- out
-	0.978	- out
-	0.979	- out
-	0.98	- out
-	0.981	- out
-	0.982	- out
-	0.983	- out
-	0.984	- out
-	0.985	- out
-	0.986	- out

-	0.987	-	out							
-	0.988	-	out							
-	0.989	-	out							
-	0.99	-	out							
-	0.991	-	out							
-	0.992	-	out							
-	0.993	-	out							
-	0.994	-	out							
-	0.995	-	out							
-	0.996	-	out							
-	0.997	-	out							
-	0.998	-	out							
-	0.999	-	out							
-	1	-	out							
-	1	-	out							
44	1.1031	0.20266	94	47	94	2	0	0	2.7e-15	8.9e-15
45	1.5084	0.40532	96	48	96	2	0	0	5.3e-15	1.6e-14
46	1.9137	0.40532	98	49	98	2	0	0	1.7e-15	1.4e-14
-	2	-	out							
47	2.319	0.40532	100	50	100	2	0	0	2.9e-15	1.6e-14
48	2.7243	0.40532	102	51	102	2	0	0	3.3e-15	1.2e-14
-	3	-	out							
49	3.535	0.81064	104	52	104	2	0	0	4.2e-15	1.5e-14
-	4	-	out							
50	4.3456	0.81064	106	53	106	2	0	0	3.3e-15	1.2e-14
-	5	-	out							
51	5.9669	1.6213	108	54	108	2	0	0	6.3e-15	2.1e-14
-	6	-	out							
-	7	-	out							
52	7.5882	1.6213	110	55	110	2	0	0	1.1e-14	1.8e-14
-	8	-	out							
-	9	-	out							
53	9.2095	1.6213	112	56	112	2	0	0	7.6e-15	1.7e-14
-	10	-	out							
54	10.831	1.6213	114	57	114	2	0	0	3.8e-15	1.3e-14
-	11	-	out							
-	12	-	out							
-	13	-	out							
-	14	-	out							
55	14.073	3.2426	116	58	116	2	0	0	2.2e-15	1.7e-14
-	15	-	out							
-	16	-	out							
-	17	-	out							
56	17.316	3.2426	118	59	118	2	0	0	1.3e-14	1.4e-14
-	18	-	out							
-	19	-	out							
-	20	-	out							
-	21	-	out							
-	22	-	out							
-	23	-	out							
57	23.801	6.4851	120	60	120	2	0	0	4.1e-15	2.2e-14
-	24	-	out							
-	25	-	out							
-	26	-	out							
-	27	-	out							
-	28	-	out							

-	29	-	out							
-	30	-	out							
58	30.286	6.4851	122	61	122	2	0	0	2.9e-15	2.2e-14
-	31	-	out							
-	32	-	out							
-	33	-	out							
-	34	-	out							
-	35	-	out							
-	36	-	out							
59	36.771	6.4851	124	62	124	2	0	0	1.3e-14	2.2e-14
-	37	-	out							
-	38	-	out							
-	39	-	out							
-	40	-	out							
-	41	-	out							
-	42	-	out							
-	43	-	out							
-	44	-	out							
-	45	-	out							
-	46	-	out							
-	47	-	out							
-	48	-	out							
-	49	-	out							
60	49.741	12.97	126	63	126	2	0	0	9.2e-15	2.9e-14
-	50	-	out							
-	51	-	out							
-	52	-	out							
-	53	-	out							
-	54	-	out							
-	55	-	out							
-	56	-	out							
-	57	-	out							
-	58	-	out							
-	59	-	out							
-	60	-	out							
-	61	-	out							
-	62	-	out							
61	62.712	12.97	128	64	128	2	0	0	1.3e-14	2.2e-14
-	63	-	out							
-	64	-	out							
-	65	-	out							
-	66	-	out							
-	67	-	out							
-	68	-	out							
-	69	-	out							
-	70	-	out							
-	71	-	out							
-	72	-	out							
-	73	-	out							
-	74	-	out							
-	75	-	out							
-	76	-	out							
-	77	-	out							
-	78	-	out							
-	79	-	out							
-	80	-	out							

-	81	- out								
-	82	- out								
-	83	- out								
-	84	- out								
-	85	- out								
-	86	- out								
-	87	- out								
-	88	- out								
62	88.652	25.94	130	65	130	2	0	0	1.3e-14	3.9e-14
-	89	- out								
-	90	- out								
-	91	- out								
-	92	- out								
-	93	- out								
-	94	- out								
-	95	- out								
-	96	- out								
-	97	- out								
-	98	- out								
-	99	- out								
-	100	- out								
-	101	- out								
-	102	- out								
-	103	- out								
-	104	- out								
-	105	- out								
-	106	- out								
-	107	- out								
-	108	- out								
-	109	- out								
-	110	- out								
-	111	- out								
-	112	- out								
-	113	- out								
-	114	- out								
63	114.59	25.94	132	66	132	2	0	0	2.7e-15	3.4e-14
-	115	- out								
-	116	- out								
-	117	- out								
-	118	- out								
-	119	- out								
-	120	- out								
-	120	- out								
-	130	- out								
-	140	- out								
64	140.53	25.94	134	67	134	2	0	0	6.4e-15	3.4e-14
-	150	- out								
-	160	- out								
-	170	- out								
65	170.53	30	136	68	136	2	0	0	4.8e-15	3.3e-14
-	180	- out								
-	190	- out								
-	200	- out								
66	200.53	30	138	69	138	2	0	0	8e-15	2.8e-14
-	210	- out								
-	220	- out								

```
  -        230           - out
 67      230.53         30      140   70  140     2     0      0  1.1e-14  2.7e-14
  -        240           - out
  -        250           - out
  -        260           - out
 68      260.53         30      142   71  142     2     0      0  1.9e-14  2.3e-14
  -        270           - out
  -        280           - out
  -        290           - out
 69      290.53         30      143   72  143     2     0      0  8.1e-15    2e-14
  -        300           - out
 70      320.53         30      144   73  144     2     0      0  1.5e-14  2.1e-14
Time-stepping completed.
Solution time: 6 s.
Physical memory: 1.48 GB
Virtual memory: 1.87 GB
Ended at Mar 30, 2025, 4:09:01 PM.
----- Time-Dependent Solver 1 in Study 1/Solution 1 (sol1) -------------------->
```

Advanced (aDef)

ASSEMBLY SETTINGS

Description	Value
Reuse sparsity pattern	On

Fully Coupled 1 (fc1)

GENERAL

Description	Value
Linear solver	Direct, concentrations (tds)

METHOD AND TERMINATION

Description	Value
Damping factor	0.9
Jacobian update	Once per time step
Maximum number of iterations	8
Stabilization and acceleration	Anderson acceleration
Dimension of iteration space	5
Mixing parameter	0.9
Iteration delay	1

Direct, concentrations (tds) (d1)

GENERAL

Description	Value
Solver	PARDISO
Pivoting perturbation	1E-13

Solution Store 1 (su1)

GENERAL

Description	Value
Solution	Solution Store 1

Compile Equations: Pipette Transfer (st2)

STUDY AND STEP

Description	Value
Use study	Study 1
Use study step	Pipette Transfer

GEOMETRIC ENTITY SELECTION

Description	Value
Use entities	Selected
Selections	{Lower pipet_level 1, Lower level 2}

LOG

```
<---- Compile Equations: Pipette Transfer in Study 1/Solution 1 (sol1) ---------
Started at Mar 30, 2025, 4:09:01 PM.
Geometry shape function: Linear Lagrange
Time: 1 s.
Physical memory: 1.53 GB
Virtual memory: 1.92 GB
Ended at Mar 30, 2025, 4:09:02 PM.
----- Compile Equations: Pipette Transfer in Study 1/Solution 1 (sol1) -------->
```

Dependent Variables 2 (v2)

GENERAL

Description	Value
Defined by study step	Pipette Transfer

INITIAL VALUES OF VARIABLES SOLVED FOR

Description	Value
Method	Solution
Solution	Solution 1

RESIDUAL SCALING

Description	Value
Method	Manual

VALUES OF VARIABLES NOT SOLVED FOR

Description	Value

Description	Value
Method	Solution
Solution	Solution 1

INITIAL VALUE CALCULATION CONSTANTS

Constant name	Initial value source
t	range(300,0.001,301), range(301,1,390)
timestep	0.09[s]

LOG

```
<---- Dependent Variables 2 in Study 1/Solution 1 (sol1) -----------------------
Started at Mar 30, 2025, 4:09:02 PM.
Initial values of variables solved for: Solution 1 (sol1), Solution Store 1 (sol2),
t=300 s [Automatic (single solution)].
Values of variables not solved for: Solution 1 (sol1), Solution Store 1 (sol2), t=30
0 s [Automatic (single solution)].
Solution time: 0 s.
Physical memory: 1.53 GB
Virtual memory: 1.92 GB
Ended at Mar 30, 2025, 4:09:02 PM.
----- Dependent Variables 2 in Study 1/Solution 1 (sol1) ---------------------->
```

Concentration (comp1.Cox) (comp1_Cox)

GENERAL

Description	Value
Field components	comp1.Cox
Internal variables	{comp1.uflux.Cox, comp1.dflux.Cox, comp1.tds.dt2Inv_Cox}
Store in output	Selection
Selection	{Lower level 2, Lower pipet_level 1}

Concentration (comp1.Cred) (comp1_Cred)

GENERAL

Description	Value
Field components	comp1.Cred
Internal variables	{comp1.uflux.Cred, comp1.dflux.Cred, comp1.tds.dt2Inv_Cred}
Store in output	Selection
Selection	{Lower level 2, Lower pipet_level 1}

Time-Dependent Solver 2 (t2)

GENERAL

Description	Value
Defined by study step	Pipette Transfer

Description	Value
Output times	{300, 300, 300, 300, 300, 300.01, 300.01, 300.01, 300.01, 300.01, 300.01, 300.01, 300.01, 300.01, 300.01, 300.02, 300.02, 300.02, 300.02, 300.02, 300.02, 300.02, 300.02, 300.02, 300.02, 300.03, 300.03, 300.03, 300.03, 300.03, 300.03, 300.03, 300.03, 300.03, 300.03, 300.04, 300.04, 300.04, 300.04, 300.04, 300.04, 300.04, 300.04, 300.04, 300.04, 300.05, 300.05, 300.05, 300.05, 300.05, 300.05, 300.05, 300.05, 300.05, 300.05, 300.06, 300.06, 300.06, 300.06, 300.06, 300.06, 300.06, 300.06, 300.06, 300.06, 300.07, 300.07, 300.07, 300.07, 300.07, 300.07, 300.07, 300.07, 300.07, 300.07, 300.08, 300.08, 300.08, 300.08, 300.08, 300.08, 300.08, 300.08, 300.08, 300.08, 300.09, 300.09, 300.09, 300.09, 300.09, 300.09, 300.09, 300.09, 300.09, 300.09, 300.1, 300.1, 300.1, 300.1, 300.1, 300.1, 300.1, 300.1, 300.1, 300.1, 300.11, 300.11, 300.11, 300.11, 300.11, 300.11, 300.11, 300.11, 300.11, 300.11, 300.12, 300.12, 300.12, 300.12, 300.12, 300.12, 300.12, 300.12, 300.12, 300.12, 300.13, 300.13, 300.13, 300.13, 300.13, 300.13, 300.13, 300.13, 300.13, 300.13, 300.14, 300.14, 300.14, 300.14, 300.14, 300.14, 300.14, 300.14, 300.14, 300.14, 300.15, 300.15, 300.15, 300.15, 300.15, 300.15, 300.15, 300.15, 300.15, 300.15, 300.16, 300.16, 300.16, 300.16, 300.16, 300.16, 300.16, 300.16, 300.16, 300.16, 300.17, 300.17, 300.17, 300.17, 300.17, 300.17, 300.17, 300.17, 300.17, 300.17, 300.18, 300.18, 300.18, 300.18, 300.18, 300.18, 300.18, 300.18, 300.18, 300.18, 300.19, 300.19, 300.19, 300.19, 300.19, 300.19, 300.19, 300.19, 300.19, 300.19, 300.2, 300.2, 300.2, 300.2, 300.2, 300.2, 300.2, 300.2, 300.2, 300.2, 300.21, 300.21, 300.21, 300.21, 300.21, 300.21, 300.21, 300.21, 300.21, 300.21, 300.22, 300.22, 300.22, 300.22, 300.22, 300.22, 300.22, 300.22, 300.22, 300.22, 300.23, 300.23, 300.23, 300.23, 300.23, 300.23, 300.23, 300.23, 300.23, 300.23, 300.24, 300.24, 300.24, 300.24, 300.24, 300.24, 300.24, 300.24, 300.24, 300.24, 300.25, 300.25, 300.25, 300.25, 300.25, 300.25, 300.25, 300.25, 300.25, 300.25, 300.26, 300.26, 300.26, 300.26, 300.26, 300.26, 300.26, 300.26, 300.26, 300.26, 300.27, 300.27, 300.27, 300.27, 300.27, 300.27, 300.27, 300.27, 300.27, 300.27, 300.28, 300.28, 300.28, 300.28, 300.28, 300.28, 300.28, 300.28, 300.28, 300.28, 300.29, 300.29, 300.29, 300.29, 300.29, 300.29, 300.29, 300.29, 300.29, 300.29, 300.3, 300.3, 300.3, 300.3, 300.3, 300.3, 300.3, 300.3, 300.3, 300.3, 300.31, 300.31, 300.31, 300.31, 300.31, 300.31, 300.31, 300.31, 300.31, 300.31, 300.32, 300.32, 300.32, 300.32, 300.32, 300.32, 300.32, 300.32, 300.32, 300.32, 300.33, 300.33, 300.33, 300.33, 300.33, 300.33, 300.33, 300.33, 300.33, 300.33, 300.34, 300.34, 300.34, 300.34, 300.34, 300.34, 300.34, 300.34, 300.34, 300.34, 300.35, 300.35, 300.35, 300.35, 300.35, 300.35, 300.35, 300.35, 300.35, 300.35, 300.36, 300.36, 300.36, 300.36, 300.36, 300.36, 300.36, 300.36, 300.36, 300.36, 300.37, 300.37, 300.37, 300.37, 300.37, 300.37, 300.37, 300.37, 300.37, 300.37, 300.38, 300.38, 300.38, 300.38, 300.38, 300.38, 300.38, 300.38, 300.38, 300.38, 300.39, 300.39, 300.39, 300.39, 300.39, 300.39, 300.39, 300.39, 300.39, 300.39, 300.4, 300.4, 300.4, 300.4, 300.4, 300.4, 300.4, 300.4, 300.4, 300.4, 300.41, 300.41, 300.41, 300.41, 300.41, 300.41, 300.41, 300.41, 300.41, 300.41, 300.42, 300.42, 300.42, 300.42, 300.42, 300.42, 300.42, 300.42, 300.42, 300.42, 300.43, 300.43, 300.43, 300.43, 300.43, 300.43, 300.43, 300.43, 300.43, 300.43,

Description	Value
	300.44, 300.44, 300.44, 300.44, 300.44, 300.44, 300.44, 300.44, 300.44, 300.44, 300.45, 300.45, 300.45, 300.45, 300.45, 300.45, 300.45, 300.45, 300.45, 300.45, 300.46, 300.46, 300.46, 300.46, 300.46, 300.46, 300.46, 300.46, 300.46, 300.46, 300.47, 300.47, 300.47, 300.47, 300.47, 300.47, 300.47, 300.47, 300.47, 300.47, 300.48, 300.48, 300.48, 300.48, 300.48, 300.48, 300.48, 300.48, 300.48, 300.48, 300.49, 300.49, 300.49, 300.49, 300.49, 300.49, 300.49, 300.49, 300.49, 300.49, 300.5, 300.5, 300.5, 300.5, 300.5, 300.5, 300.5, 300.5, 300.5, 300.5, 300.51, 300.51, 300.51, 300.51, 300.51, 300.51, 300.51, 300.51, 300.51, 300.51, 300.52, 300.52, 300.52, 300.52, 300.52, 300.52, 300.52, 300.52, 300.52, 300.52, 300.53, 300.53, 300.53, 300.53, 300.53, 300.53, 300.53, 300.53, 300.53, 300.53, 300.54, 300.54, 300.54, 300.54, 300.54, 300.54, 300.54, 300.54, 300.54, 300.54, 300.55, 300.55, 300.55, 300.55, 300.55, 300.55, 300.55, 300.55, 300.55, 300.55, 300.56, 300.56, 300.56, 300.56, 300.56, 300.56, 300.56, 300.56, 300.56, 300.56, 300.57, 300.57, 300.57, 300.57, 300.57, 300.57, 300.57, 300.57, 300.57, 300.57, 300.58, 300.58, 300.58, 300.58, 300.58, 300.58, 300.58, 300.58, 300.58, 300.58, 300.59, 300.59, 300.59, 300.59, 300.59, 300.59, 300.59, 300.59, 300.59, 300.59, 300.6, 300.6, 300.6, 300.6, 300.6, 300.6, 300.6, 300.6, 300.6, 300.6, 300.6, 300.6, 300.6, 300.6, 300.61, 300.61, 300.61, 300.61, 300.61, 300.61, 300.61, 300.61, 300.61, 300.61, 300.62, 300.62, 300.62, 300.62, 300.62, 300.62, 300.62, 300.62, 300.62, 300.62, 300.63, 300.63, 300.63, 300.63, 300.63, 300.63, 300.63, 300.63, 300.63, 300.63, 300.64, 300.64, 300.64, 300.64, 300.64, 300.64, 300.64, 300.64, 300.64, 300.64, 300.65, 300.65, 300.65, 300.65, 300.65, 300.65, 300.65, 300.65, 300.65, 300.65, 300.66, 300.66, 300.66, 300.66, 300.66, 300.66, 300.66, 300.66, 300.66, 300.66, 300.67, 300.67, 300.67, 300.67, 300.67, 300.67, 300.67, 300.67, 300.67, 300.67, 300.68, 300.68, 300.68, 300.68, 300.68, 300.68, 300.68, 300.68, 300.68, 300.68, 300.69, 300.69, 300.69, 300.69, 300.69, 300.69, 300.69, 300.69, 300.69, 300.69, 300.7, 300.7, 300.7, 300.7, 300.7, 300.7, 300.7, 300.7, 300.7, 300.7, 300.71, 300.71, 300.71, 300.71, 300.71, 300.71, 300.71, 300.71, 300.71, 300.71, 300.72, 300.72, 300.72, 300.72, 300.72, 300.72, 300.72, 300.72, 300.72, 300.72, 300.73, 300.73, 300.73, 300.73, 300.73, 300.73, 300.73, 300.73, 300.73, 300.73, 300.74, 300.74, 300.74, 300.74, 300.74, 300.74, 300.74, 300.74, 300.74, 300.74, 300.75, 300.75, 300.75, 300.75, 300.75, 300.75, 300.75, 300.75, 300.75, 300.75, 300.76, 300.76, 300.76, 300.76, 300.76, 300.76, 300.76, 300.76, 300.76, 300.76, 300.77, 300.77, 300.77, 300.77, 300.77, 300.77, 300.77, 300.77, 300.77, 300.77, 300.78, 300.78, 300.78, 300.78, 300.78, 300.78, 300.78, 300.78, 300.78, 300.78, 300.79, 300.79, 300.79, 300.79, 300.79, 300.79, 300.79, 300.79, 300.79, 300.79, 300.8, 300.8, 300.8, 300.8, 300.8, 300.8, 300.8, 300.8, 300.8, 300.8, 300.81, 300.81, 300.81, 300.81, 300.81, 300.81, 300.81, 300.81, 300.81, 300.81, 300.82, 300.82, 300.82, 300.82, 300.82, 300.82, 300.82, 300.82, 300.82, 300.82, 300.83, 300.83, 300.83, 300.83, 300.83, 300.83, 300.83, 300.83, 300.83, 300.83, 300.84, 300.84, 300.84, 300.84, 300.84, 300.84, 300.84, 300.84, 300.84, 300.84, 300.85, 300.85, 300.85, 300.85, 300.85, 300.85, 300.85, 300.85, 300.85, 300.85, 300.86, 300.86, 300.86, 300.86, 300.86, 300.86, 300.86, 300.86, 300.86, 300.86, 300.87, 300.87, 300.87, 300.87, 300.87, 300.87, 300.87, 300.87, 300.87, 300.87, 300.88,

Description	Value
	300.88, 300.88, 300.88, 300.88, 300.88, 300.88, 300.88, 300.88, 300.88, 300.89, 300.89, 300.89, 300.89, 300.89, 300.89, 300.89, 300.89, 300.89, 300.89, 300.9, 300.9, 300.9, 300.9, 300.9, 300.9, 300.9, 300.9, 300.9, 300.9, 300.91, 300.91, 300.91, 300.91, 300.91, 300.91, 300.91, 300.91, 300.91, 300.91, 300.92, 300.92, 300.92, 300.92, 300.92, 300.92, 300.92, 300.92, 300.92, 300.92, 300.93, 300.93, 300.93, 300.93, 300.93, 300.93, 300.93, 300.93, 300.93, 300.93, 300.94, 300.94, 300.94, 300.94, 300.94, 300.94, 300.94, 300.94, 300.94, 300.94, 300.95, 300.95, 300.95, 300.95, 300.95, 300.95, 300.95, 300.95, 300.95, 300.95, 300.96, 300.96, 300.96, 300.96, 300.96, 300.96, 300.96, 300.96, 300.96, 300.96, 300.97, 300.97, 300.97, 300.97, 300.97, 300.97, 300.97, 300.97, 300.97, 300.97, 300.98, 300.98, 300.98, 300.98, 300.98, 300.98, 300.98, 300.98, 300.98, 300.98, 300.99, 300.99, 300.99, 300.99, 300.99, 300.99, 300.99, 300.99, 300.99, 300.99, 300.99, 301, 301, 301, 301, 301, 301, 301, 302, 303, 304, 305, 306, 307, 308, 309, 310, 311, 312, 313, 314, 315, 316, 317, 318, 319, 320, 321, 322, 323, 324, 325, 326, 327, 328, 329, 330, 331, 332, 333, 334, 335, 336, 337, 338, 339, 340, 341, 342, 343, 344, 345, 346, 347, 348, 349, 350, 351, 352, 353, 354, 355, 356, 357, 358, 359, 360, 361, 362, 363, 364, 365, 366, 367, 368, 369, 370, 371, 372, 373, 374, 375, 376, 377, 378, 379, 380, 381, 382, 383, 384, 385, 386, 387, 388, 389, 390}
Relative tolerance	0.005

TIME STEPPING

Description	Value
Maximum BDF order	2
Nonlinear controller	On

LOG

-	300.33	-	out							
-	300.33	-	out							
-	300.33	-	out							
-	300.33	-	out							
-	300.34	-	out							
-	300.34	-	out							
-	300.34	-	out							
-	300.34	-	out							
-	300.34	-	out							
-	300.34	-	out							
-	300.34	-	out							
-	300.34	-	out							
-	300.34	-	out							
-	300.35	-	out							
-	300.35	-	out							
-	300.35	-	out							
-	300.35	-	out							
-	300.35	-	out							
-	300.35	-	out							
-	300.35	-	out							
-	300.35	-	out							
-	300.35	-	out							
-	300.35	-	out							
-	300.36	-	out							
-	300.36	-	out							
-	300.36	-	out							
-	300.36	-	out							
-	300.36	-	out							
-	300.36	-	out							
-	300.36	-	out							
-	300.36	-	out							
-	300.36	-	out							
-	300.36	-	out							
-	300.37	-	out							
-	300.37	-	out							
-	300.37	-	out							
-	300.37	-	out							
-	300.37	-	out							
-	300.37	-	out							
-	300.37	-	out							
-	300.37	-	out							
24	300.37	0.067071	52	27	52	2	0	0	2.3e-15	8.7e-15
-	300.37	-	out							
-	300.37	-	out							
-	300.38	-	out							
-	300.38	-	out							
-	300.38	-	out							
-	300.38	-	out							
-	300.38	-	out							
-	300.38	-	out							
-	300.38	-	out							
-	300.38	-	out							
-	300.38	-	out							
-	300.38	-	out							
-	300.38	-	out							
-	300.39	-	out							

-	300.39	-	out							
-	300.39	-	out							
-	300.39	-	out							
-	300.39	-	out							
-	300.39	-	out							
-	300.39	-	out							
-	300.39	-	out							
-	300.39	-	out							
-	300.39	-	out							
-	300.4	-	out							
-	300.4	-	out							
-	300.4	-	out							
-	300.4	-	out							
-	300.4	-	out							
-	300.4	-	out							
-	300.4	-	out							
-	300.4	-	out							
-	300.4	-	out							
-	300.4	-	out							
-	300.41	-	out							
-	300.41	-	out							
-	300.41	-	out							
-	300.41	-	out							
-	300.41	-	out							
-	300.41	-	out							
-	300.41	-	out							
-	300.41	-	out							
-	300.41	-	out							
-	300.42	-	out							
-	300.42	-	out							
-	300.42	-	out							
-	300.42	-	out							
-	300.42	-	out							
-	300.42	-	out							
-	300.42	-	out							
-	300.42	-	out							
-	300.42	-	out							
-	300.42	-	out							
-	300.43	-	out							
-	300.43	-	out							
-	300.43	-	out							
-	300.43	-	out							
-	300.43	-	out							
-	300.43	-	out							
-	300.43	-	out							
-	300.43	-	out							
-	300.43	-	out							
-	300.43	-	out							
-	300.44	-	out							
-	300.44	-	out							
-	300.44	-	out							
-	300.44	-	out							
-	300.44	-	out							
25	300.44	0.067071	54	28	54	2	0	0	3.6e-15	7.5e-15
-	300.44	-	out							
-	300.44	-	out							

-	300.44	- out
-	300.44	- out
-	300.44	- out
-	300.44	- out
-	300.45	- out
-	300.45	- out
-	300.45	- out
-	300.45	- out
-	300.45	- out
-	300.45	- out
-	300.45	- out
-	300.45	- out
-	300.45	- out
-	300.45	- out
-	300.46	- out
-	300.46	- out
-	300.46	- out
-	300.46	- out
-	300.46	- out
-	300.46	- out
-	300.46	- out
-	300.46	- out
-	300.46	- out
-	300.46	- out
-	300.47	- out
-	300.47	- out
-	300.47	- out
-	300.47	- out
-	300.47	- out
-	300.47	- out
-	300.47	- out
-	300.47	- out
-	300.47	- out
-	300.48	- out
-	300.48	- out
-	300.48	- out
-	300.48	- out
-	300.48	- out
-	300.48	- out
-	300.48	- out
-	300.48	- out
-	300.48	- out
-	300.48	- out
-	300.49	- out
-	300.49	- out
-	300.49	- out
-	300.49	- out
-	300.49	- out
-	300.49	- out
-	300.49	- out
-	300.49	- out
-	300.49	- out
-	300.49	- out
-	300.5	- out
-	300.5	- out
-	300.5	- out

-	300.5	-	out							
-	300.5	-	out							
-	300.5	-	out							
-	300.5	-	out							
-	300.5	-	out							
-	300.5	-	out							
-	300.5	-	out							
-	300.5	-	out							
-	300.51	-	out							
26	300.51	0.067071	56	29	56	2	0	0	2.2e-15	7.6e-15
-	300.51	-	out							
-	300.51	-	out							
-	300.51	-	out							
-	300.51	-	out							
-	300.51	-	out							
-	300.51	-	out							
-	300.51	-	out							
-	300.51	-	out							
-	300.51	-	out							
-	300.52	-	out							
-	300.52	-	out							
-	300.52	-	out							
-	300.52	-	out							
-	300.52	-	out							
-	300.52	-	out							
-	300.52	-	out							
-	300.52	-	out							
-	300.52	-	out							
-	300.52	-	out							
-	300.53	-	out							
-	300.53	-	out							
-	300.53	-	out							
-	300.53	-	out							
-	300.53	-	out							
-	300.53	-	out							
-	300.53	-	out							
-	300.53	-	out							
-	300.53	-	out							
-	300.54	-	out							
-	300.54	-	out							
-	300.54	-	out							
-	300.54	-	out							
-	300.54	-	out							
-	300.54	-	out							
-	300.54	-	out							
-	300.54	-	out							
-	300.54	-	out							
-	300.54	-	out							
-	300.55	-	out							
-	300.55	-	out							
-	300.55	-	out							
-	300.55	-	out							
-	300.55	-	out							
-	300.55	-	out							
-	300.55	-	out							
-	300.55	-	out							

-	300.55	- out
-	300.55	- out
-	300.56	- out
-	300.56	- out
-	300.56	- out
-	300.56	- out
-	300.56	- out
-	300.56	- out
-	300.56	- out
-	300.56	- out
-	300.56	- out
-	300.56	- out
-	300.56	- out
-	300.57	- out
-	300.57	- out
-	300.57	- out
-	300.57	- out
-	300.57	- out
-	300.57	- out
-	300.57	- out
-	300.57	- out
-	300.57	- out
-	300.57	- out
-	300.58	- out
-	300.58	- out
-	300.58	- out
-	300.58	- out
-	300.58	- out
-	300.58	- out
-	300.58	- out
-	300.58	- out
-	300.58	- out
-	300.58	- out
-	300.59	- out
-	300.59	- out
-	300.59	- out
-	300.59	- out
-	300.59	- out
-	300.59	- out
-	300.59	- out
-	300.59	- out
-	300.59	- out
-	300.6	- out
-	300.6	- out
-	300.6	- out
-	300.6	- out
-	300.6	- out
-	300.6	- out
-	300.6	- out
-	300.6	- out
-	300.6	- out
-	300.6	- out
-	300.61	- out
-	300.61	- out
-	300.61	- out
-	300.61	- out

-	300.61	-	out							
-	300.61	-	out							
-	300.61	-	out							
-	300.61	-	out							
-	300.61	-	out							
-	300.61	-	out							
-	300.62	-	out							
-	300.62	-	out							
-	300.62	-	out							
-	300.62	-	out							
-	300.62	-	out							
-	300.62	-	out							
-	300.62	-	out							
-	300.62	-	out							
-	300.62	-	out							
-	300.62	-	out							
-	300.63	-	out							
-	300.63	-	out							
-	300.63	-	out							
-	300.63	-	out							
-	300.63	-	out							
-	300.63	-	out							
-	300.63	-	out							
-	300.63	-	out							
-	300.63	-	out							
-	300.63	-	out							
-	300.63	-	out							
-	300.64	-	out							
-	300.64	-	out							
-	300.64	-	out							
-	300.64	-	out							
-	300.64	-	out							
27	300.64	0.13414	58	30	58	2	0	0	5.1e-15	1.2e-14
-	300.64	-	out							
-	300.64	-	out							
-	300.64	-	out							
-	300.64	-	out							
-	300.64	-	out							
-	300.65	-	out							
-	300.65	-	out							
-	300.65	-	out							
-	300.65	-	out							
-	300.65	-	out							
-	300.65	-	out							
-	300.65	-	out							
-	300.65	-	out							
-	300.65	-	out							
-	300.65	-	out							
-	300.66	-	out							
-	300.66	-	out							
-	300.66	-	out							
-	300.66	-	out							
-	300.66	-	out							
-	300.66	-	out							
-	300.66	-	out							
-	300.66	-	out							

-	300.66	- out
-	300.67	- out
-	300.67	- out
-	300.67	- out
-	300.67	- out
-	300.67	- out
-	300.67	- out
-	300.67	- out
-	300.67	- out
-	300.67	- out
-	300.67	- out
-	300.68	- out
-	300.68	- out
-	300.68	- out
-	300.68	- out
-	300.68	- out
-	300.68	- out
-	300.68	- out
-	300.68	- out
-	300.68	- out
-	300.68	- out
-	300.69	- out
-	300.69	- out
-	300.69	- out
-	300.69	- out
-	300.69	- out
-	300.69	- out
-	300.69	- out
-	300.69	- out
-	300.69	- out
-	300.69	- out
-	300.69	- out
-	300.7	- out
-	300.7	- out
-	300.7	- out
-	300.7	- out
-	300.7	- out
-	300.7	- out
-	300.7	- out
-	300.7	- out
-	300.7	- out
-	300.7	- out
-	300.71	- out
-	300.71	- out
-	300.71	- out
-	300.71	- out
-	300.71	- out
-	300.71	- out
-	300.71	- out
-	300.71	- out
-	300.71	- out
-	300.71	- out
-	300.72	- out
-	300.72	- out
-	300.72	- out
-	300.72	- out

-	300.72	-	out							
-	300.72	-	out							
-	300.72	-	out							
-	300.72	-	out							
-	300.72	-	out							
-	300.73	-	out							
-	300.73	-	out							
-	300.73	-	out							
-	300.73	-	out							
-	300.73	-	out							
-	300.73	-	out							
-	300.73	-	out							
-	300.73	-	out							
-	300.73	-	out							
-	300.73	-	out							
-	300.74	-	out							
-	300.74	-	out							
-	300.74	-	out							
-	300.74	-	out							
-	300.74	-	out							
-	300.74	-	out							
-	300.74	-	out							
-	300.74	-	out							
-	300.74	-	out							
-	300.74	-	out							
-	300.75	-	out							
-	300.75	-	out							
-	300.75	-	out							
-	300.75	-	out							
-	300.75	-	out							
-	300.75	-	out							
-	300.75	-	out							
-	300.75	-	out							
-	300.75	-	out							
-	300.75	-	out							
-	300.75	-	out							
-	300.76	-	out							
-	300.76	-	out							
-	300.76	-	out							
-	300.76	-	out							
-	300.76	-	out							
-	300.76	-	out							
-	300.76	-	out							
-	300.76	-	out							
-	300.76	-	out							
-	300.76	-	out							
-	300.77	-	out							
-	300.77	-	out							
-	300.77	-	out							
-	300.77	-	out							
-	300.77	-	out							
-	300.77	-	out							
-	300.77	-	out							
-	300.77	-	out							
-	300.77	-	out							
28	300.77	0.13414	60	31	60	2	0	0	4.9e-15	1e-14

-	300.77	- out
-	300.78	- out
-	300.78	- out
-	300.78	- out
-	300.78	- out
-	300.78	- out
-	300.78	- out
-	300.78	- out
-	300.78	- out
-	300.78	- out
-	300.79	- out
-	300.79	- out
-	300.79	- out
-	300.79	- out
-	300.79	- out
-	300.79	- out
-	300.79	- out
-	300.79	- out
-	300.79	- out
-	300.79	- out
-	300.8	- out
-	300.8	- out
-	300.8	- out
-	300.8	- out
-	300.8	- out
-	300.8	- out
-	300.8	- out
-	300.8	- out
-	300.8	- out
-	300.8	- out
-	300.81	- out
-	300.81	- out
-	300.81	- out
-	300.81	- out
-	300.81	- out
-	300.81	- out
-	300.81	- out
-	300.81	- out
-	300.81	- out
-	300.81	- out
-	300.81	- out
-	300.82	- out
-	300.82	- out
-	300.82	- out
-	300.82	- out
-	300.82	- out
-	300.82	- out
-	300.82	- out
-	300.82	- out
-	300.82	- out
-	300.82	- out
-	300.83	- out
-	300.83	- out
-	300.83	- out
-	300.83	- out
-	300.83	- out

-	300.83	- out
-	300.83	- out
-	300.83	- out
-	300.83	- out
-	300.83	- out
-	300.84	- out
-	300.84	- out
-	300.84	- out
-	300.84	- out
-	300.84	- out
-	300.84	- out
-	300.84	- out
-	300.84	- out
-	300.84	- out
-	300.85	- out
-	300.85	- out
-	300.85	- out
-	300.85	- out
-	300.85	- out
-	300.85	- out
-	300.85	- out
-	300.85	- out
-	300.85	- out
-	300.85	- out
-	300.86	- out
-	300.86	- out
-	300.86	- out
-	300.86	- out
-	300.86	- out
-	300.86	- out
-	300.86	- out
-	300.86	- out
-	300.86	- out
-	300.86	- out
-	300.87	- out
-	300.87	- out
-	300.87	- out
-	300.87	- out
-	300.87	- out
-	300.87	- out
-	300.87	- out
-	300.87	- out
-	300.87	- out
-	300.87	- out
-	300.88	- out
-	300.88	- out
-	300.88	- out
-	300.88	- out
-	300.88	- out
-	300.88	- out
-	300.88	- out
-	300.88	- out
-	300.88	- out
-	300.88	- out
-	300.88	- out
-	300.89	- out

-	300.89	-	out								
-	300.89	-	out								
-	300.89	-	out								
-	300.89	-	out								
-	300.89	-	out								
-	300.89	-	out								
-	300.89	-	out								
-	300.89	-	out								
-	300.89	-	out								
-	300.9	-	out								
-	300.9	-	out								
-	300.9	-	out								
-	300.9	-	out								
-	300.9	-	out								
-	300.9	-	out								
-	300.9	-	out								
-	300.9	-	out								
-	300.9	-	out								
-	300.9	-	out								
-	300.91	-	out								
-	300.91	-	out								
-	300.91	-	out								
-	300.91	-	out								
29	300.91	0.13414		62	32	62	2	0	0	1.7e-15	9.2e-15
-	300.91	-	out								
-	300.91	-	out								
-	300.91	-	out								
-	300.91	-	out								
-	300.91	-	out								
-	300.92	-	out								
-	300.92	-	out								
-	300.92	-	out								
-	300.92	-	out								
-	300.92	-	out								
-	300.92	-	out								
-	300.92	-	out								
-	300.92	-	out								
-	300.92	-	out								
-	300.92	-	out								
-	300.93	-	out								
-	300.93	-	out								
-	300.93	-	out								
-	300.93	-	out								
-	300.93	-	out								
-	300.93	-	out								
-	300.93	-	out								
-	300.93	-	out								
-	300.93	-	out								
-	300.93	-	out								
-	300.94	-	out								
-	300.94	-	out								
-	300.94	-	out								
-	300.94	-	out								
-	300.94	-	out								
-	300.94	-	out								
-	300.94	-	out								

-	300.94	- out
-	300.94	- out
-	300.94	- out
-	300.94	- out
-	300.95	- out
-	300.95	- out
-	300.95	- out
-	300.95	- out
-	300.95	- out
-	300.95	- out
-	300.95	- out
-	300.95	- out
-	300.95	- out
-	300.95	- out
-	300.96	- out
-	300.96	- out
-	300.96	- out
-	300.96	- out
-	300.96	- out
-	300.96	- out
-	300.96	- out
-	300.96	- out
-	300.96	- out
-	300.96	- out
-	300.97	- out
-	300.97	- out
-	300.97	- out
-	300.97	- out
-	300.97	- out
-	300.97	- out
-	300.97	- out
-	300.97	- out
-	300.97	- out
-	300.98	- out
-	300.98	- out
-	300.98	- out
-	300.98	- out
-	300.98	- out
-	300.98	- out
-	300.98	- out
-	300.98	- out
-	300.98	- out
-	300.98	- out
-	300.99	- out
-	300.99	- out
-	300.99	- out
-	300.99	- out
-	300.99	- out
-	300.99	- out
-	300.99	- out
-	300.99	- out
-	300.99	- out
-	300.99	- out
-	301	- out
-	301	- out
-	301	- out

-	301	-	out							
-	301	-	out							
-	301	-	out							
-	301	-	out							
30	301.04	0.13414	64	33	64	2	0	0	1.2e-15	9.2e-15
31	301.18	0.13414	66	34	66	2	0	0	7.5e-15	7.6e-15
32	301.45	0.26828	68	35	68	2	0	0	3.2e-15	9.9e-15
33	301.71	0.26828	70	36	70	2	0	0	1.1e-14	9e-15
34	301.98	0.26828	72	37	72	2	0	0	2e-15	9.4e-15
-	302	-	out							
35	302.25	0.26828	74	38	74	2	0	0	3e-15	9.3e-15
36	302.79	0.53656	76	39	76	2	0	0	3e-15	1.3e-14
-	303	-	out							
37	303.32	0.53656	78	40	78	2	0	0	4.8e-15	1.1e-14
38	303.86	0.53656	80	41	80	2	0	0	6.3e-15	9.9e-15
-	304	-	out							
39	304.4	0.53656	82	42	82	2	0	0	2.6e-15	9.7e-15
40	304.93	0.53656	84	43	84	2	0	0	4.5e-15	8.7e-15
-	305	-	out							
-	306	-	out							
41	306.01	1.0731	86	44	86	2	0	0	5.6e-15	1e-14
-	307	-	out							
42	307.08	1.0731	88	45	88	2	0	0	4.9e-15	9.5e-15
-	308	-	out							
43	308.15	1.0731	90	46	90	2	0	0	4.3e-15	8.4e-15
-	309	-	out							
44	309.23	1.0731	92	47	92	2	0	0	4.9e-15	8e-15
-	310	-	out							
-	311	-	out							
45	311.37	2.1463	94	48	94	2	0	0	9.6e-15	1.1e-14
-	312	-	out							
-	313	-	out							
46	313.52	2.1463	96	49	96	2	0	0	6e-15	1e-14
-	314	-	out							
-	315	-	out							
47	315.66	2.1463	98	50	98	2	0	0	6e-15	9.8e-15
-	316	-	out							
-	317	-	out							
48	317.81	2.1463	100	51	100	2	0	0	5.5e-15	9.1e-15
-	318	-	out							
-	319	-	out							
-	320	-	out							
-	321	-	out							
-	322	-	out							
49	322.1	4.2925	102	52	102	2	0	0	4.7e-15	1.2e-14
-	323	-	out							
-	324	-	out							
-	325	-	out							
-	326	-	out							
50	326.4	4.2925	104	53	104	2	0	0	1.5e-14	1.1e-14
-	327	-	out							
-	328	-	out							
-	329	-	out							
-	330	-	out							
51	330.69	4.2925	106	54	106	2	0	0	8.3e-15	1.1e-14
-	331	-	out							

-	332	-	out							
-	333	-	out							
-	334	-	out							
52	334.98	4.2925	108	55	108	2	0	0	6.1e-15	9.7e-15
-	335	-	out							
-	336	-	out							
-	337	-	out							
-	338	-	out							
-	339	-	out							
53	339.27	4.2925	110	56	110	2	0	0	8.1e-15	7.7e-15
-	340	-	out							
-	341	-	out							
-	342	-	out							
-	343	-	out							
-	344	-	out							
-	345	-	out							
-	346	-	out							
-	347	-	out							
54	347.86	8.585	112	57	112	2	0	0	1.2e-14	1.3e-14
-	348	-	out							
-	349	-	out							
-	350	-	out							
-	351	-	out							
-	352	-	out							
-	353	-	out							
-	354	-	out							
-	355	-	out							
-	356	-	out							
55	356.44	8.585	114	58	114	2	0	0	7.8e-15	1.2e-14
-	357	-	out							
-	358	-	out							
-	359	-	out							
-	360	-	out							
-	361	-	out							
-	362	-	out							
-	363	-	out							
-	364	-	out							
-	365	-	out							
56	365.03	8.585	116	59	116	2	0	0	9.7e-15	1.3e-14
-	366	-	out							
-	367	-	out							
-	368	-	out							
-	369	-	out							
-	370	-	out							
-	371	-	out							
-	372	-	out							
-	373	-	out							
57	373.61	8.585	118	60	118	2	0	0	7.4e-15	1.1e-14
-	374	-	out							
-	375	-	out							
-	376	-	out							
-	377	-	out							
-	378	-	out							
-	379	-	out							
-	380	-	out							
-	381	-	out							

```
   -         382          - out
  58       382.2        8.585       120    61   120      2      0       0   6.2e-15   9.3e-15
   -         383          - out
   -         384          - out
   -         385          - out
   -         386          - out
   -         387          - out
   -         388          - out
   -         389          - out
   -         390          - out
  59      390.78        8.585       122    62   122      2      0       0   9.7e-15     9e-15
Time-stepping completed.
Solution time: 5 s.
Physical memory: 1.54 GB
Virtual memory: 1.92 GB
Ended at Mar 30, 2025, 4:09:07 PM.
----- Time-Dependent Solver 2 in Study 1/Solution 1 (sol1) -------------------->
```

Advanced (aDef)

ASSEMBLY SETTINGS

Description	Value
Reuse sparsity pattern	On

Fully Coupled 1 (fc1)

GENERAL

Description	Value
Linear solver	Direct, concentrations (tds)

METHOD AND TERMINATION

Description	Value
Damping factor	0.9
Jacobian update	Once per time step
Maximum number of iterations	8
Stabilization and acceleration	Anderson acceleration
Dimension of iteration space	5
Mixing parameter	0.9
Iteration delay	1

Direct, concentrations (tds) (d1)

GENERAL

Description	Value
Solver	PARDISO
Pivoting perturbation	1E-13

Solution Store 2 (su2)

GENERAL

Description	Value
Solution	Solution Store 2

4 Results

4.1 DATASETS

4.1.1 Study 1/Solution 1

SOLUTION

Description	Value
Solution	Solution 1
Component	Component 1 (comp1)

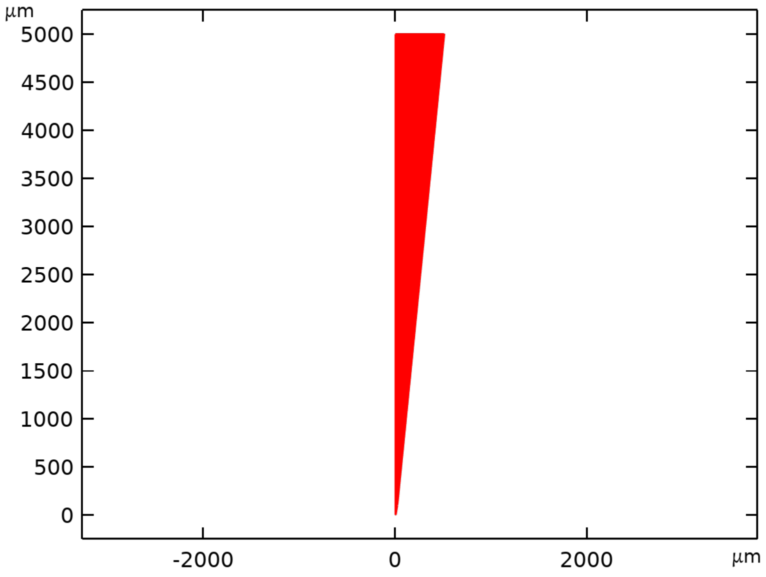

Dataset: Study 1/Solution 1

4.1.2 Revolution 2D 1

DATA

Description	Value
Dataset	Study 1/Solution 1

AXIS DATA

Description	Value
Axis entry method	Two points
Points	{{0, 0}, {0, 1}}

REVOLUTION LAYERS

Description	Value
Start angle	-90

Description	Value
Revolution angle	225

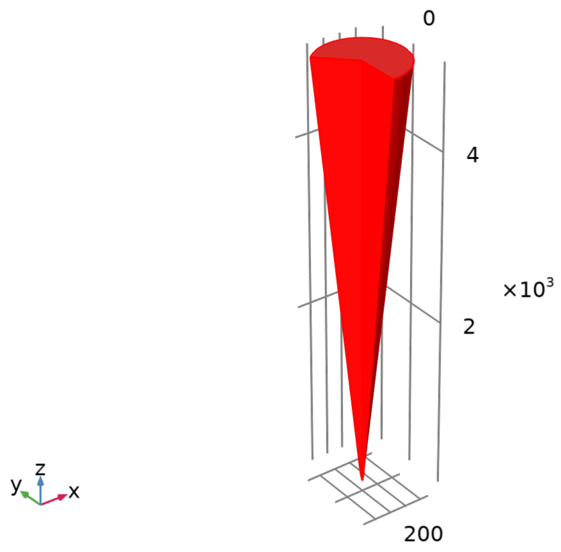

Dataset: Revolution 2D 1

4.1.3 Study 1/Solution 1

SOLUTION

Description	Value
Solution	Solution 1
Component	Component 1 (comp1)

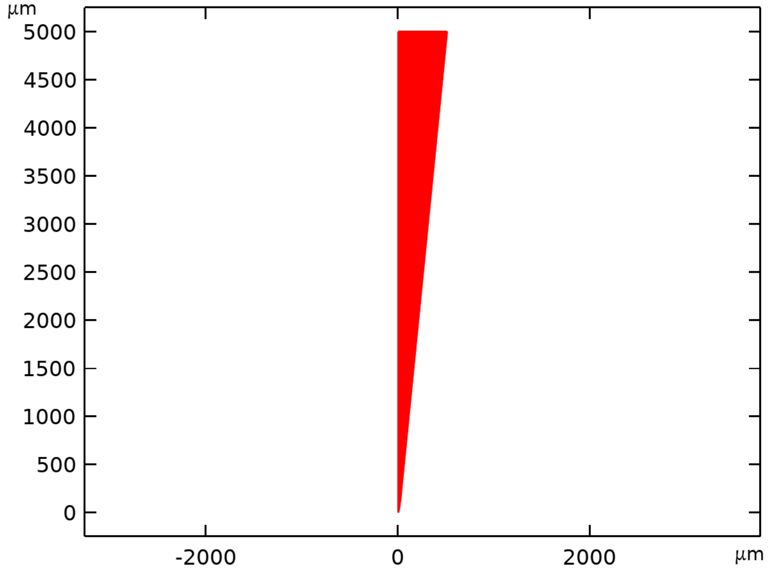

Dataset: Study 1/Solution 1

4.1.4 Boundary Probe 1

SELECTION

Geometric entity level	Boundary
Selection	Geometry geom1: Dimension 1: Boundary 2

DATA

Description	Value
Dataset	Probe Solution 3

SETTINGS

Description	Value
Method	Integration
Integration order	4
Integration order	On

4.1.5 z

DATA

Description	Value
Dataset	Study 1/Solution 1

LINE DATA

Description	Value
Line entry method	Two points
Points	{{0, Dh}, {0, 500}}
Snapping	Snap to closest boundary

ADVANCED

Description	Value
Space variable	cln1x
Normal variables	{cln1nx, cln1ny}
Tangent variables	{cln1tx, cln1ty}

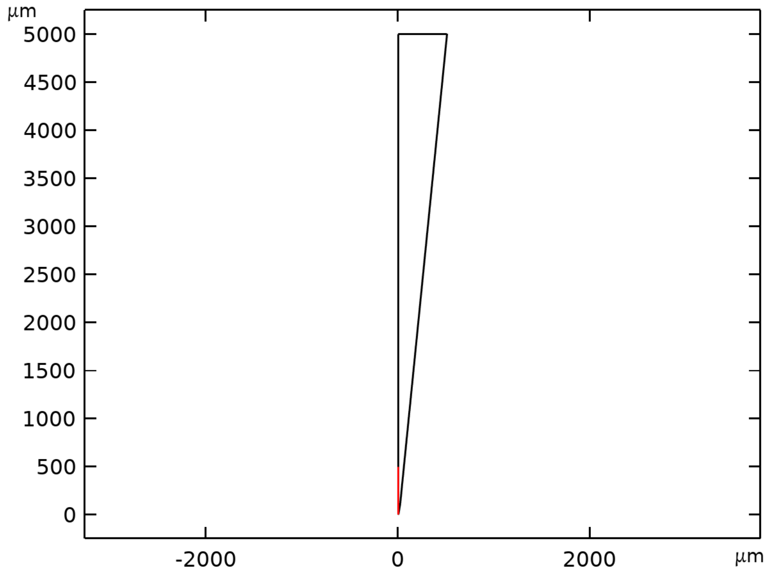

Dataset: z

4.1.6 Probe Solution 3

SOLUTION

Description	Value
Solution	Solution 1
Component	Component 1 (comp1)

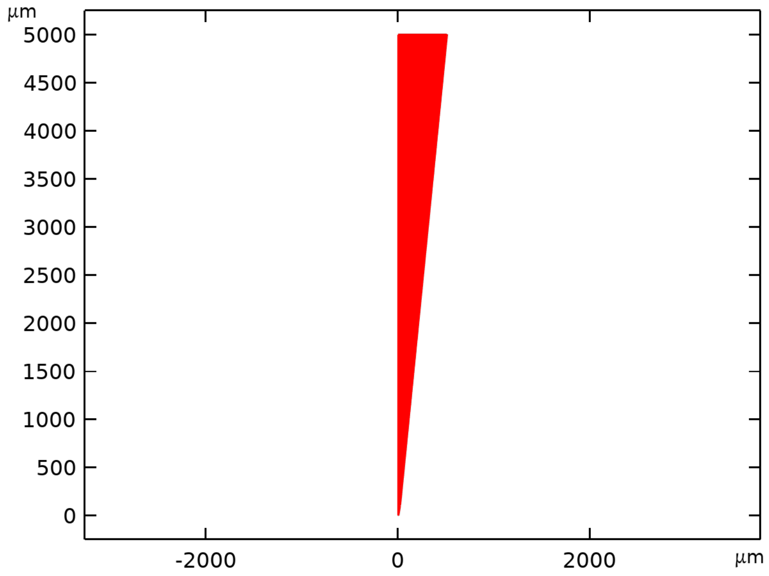

Dataset: Probe Solution 3

4.1.7 Cut Line 3D 3

DATA

Description	Value
Dataset	Revolution 2D 1

LINE DATA

Description	Value
Line entry method	Two points
Points	{{22.333, -0.024582, 0}, {22.333, -0.024582, 5000.2}}

ADVANCED

Description	Value
Space variable	cln3x
Tangent variables	{cln3tx, cln3ty, cln3tz}

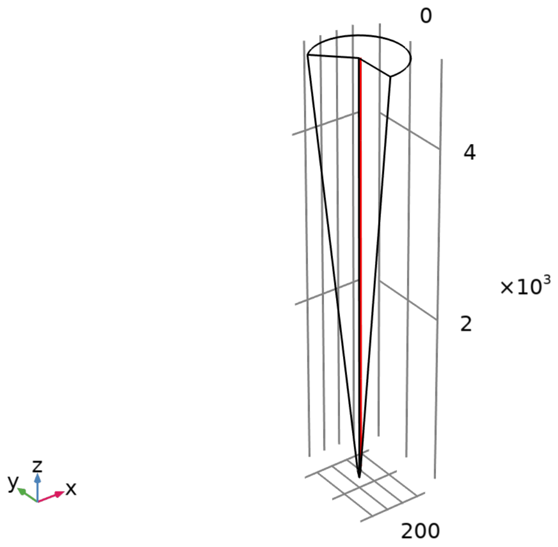

Dataset: Cut Line 3D 3

4.1.8 Cut Plane 1

DATA

Description	Value
Dataset	Revolution 2D 1

PLANE DATA

Description	Value
Plane type	General
Plane entry method	Three points
Points	{{22.333, -0.024582, 2500.1}, {22.333, 0.97542, 2500.1}, {22.333, -0.024582, 2501.1}}

ADVANCED

Description	Value
Space variables	{cpl1x, cpl1y}
Normal variables	{cpl1nx, cpl1ny, cpl1nz}

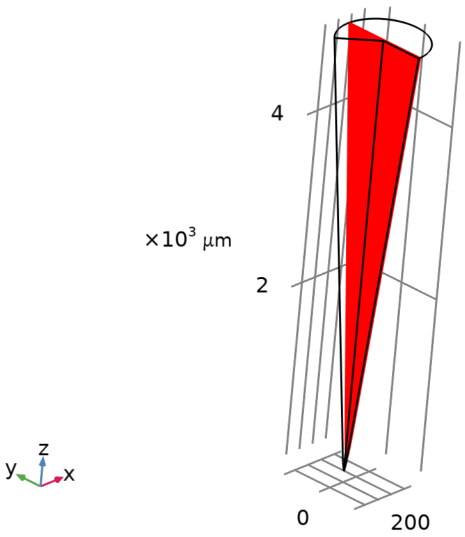

Dataset: Cut Plane 1

4.2 DERIVED VALUES

4.2.1 Point Evaluation 1

DATA

Description	Value
Dataset	Study 1/Solution 1

EXPRESSIONS

Expression	Unit	Description
tds.tflux_Coxz	mol/(m^2*s)	Total flux, z component

4.2.2 Boundary Probe 1

OUTPUT

Evaluated in	Probe Table 1

DATA

Description	Value
Dataset	Boundary Probe 1

EXPRESSIONS

Expression	Unit	Description
(tds.ntflux_Cox)*F*2	nA	

4.3 TABLES

4.3.1 Evaluation 3D

Interactive 3D values

x	y	z	Value
-0.11846	0.11846	10.068	8.5273
0.18383	0.079579	2.7173	9.9998

4.3.2 Evaluation 2D

Interactive 2D values

x	y	Value
7.1636	262.55	10
0.58462	32.843	9.7741

4.3.3 Probe Table 1

Time (s)	(tds.ntflux_Cox)*F*2 (nA), Boundary Probe 1
300	0
300	0
300	0
300	0
300	0
300	0
300	0
300	0
300.01	0
300.01	0
300.01	0
300.02	0
300.02	0
300.03	0
300.04	0
300.05	0

Time (s)	(tds.ntflux_Cox)*F*2 (nA), Boundary Probe 1
300.07	0
300.09	0
300.1	0
300.14	0
300.17	0
300.2	0
300.24	0
300.31	0
300.37	0
300.44	0
300.51	0
300.64	0
300.77	0
300.91	0
301.04	0
301.18	0
301.45	0
301.71	0
301.98	0
302.25	0
302.79	0
303.32	0
303.86	0
304.4	0
304.93	0
306.01	0
307.08	0
308.15	0
309.23	0
311.37	0
313.52	0
315.66	0
317.81	0
322.1	0
326.4	0

Time (s)	(tds.ntflux_Cox)*F*2 (nA), Boundary Probe 1
330.69	0
334.98	0
339.27	0
347.86	0
356.44	0
365.03	0
373.61	0
382.2	0
390.78	0

4.4 PLOT GROUPS

4.4.1 Concentration, Cox (tds)

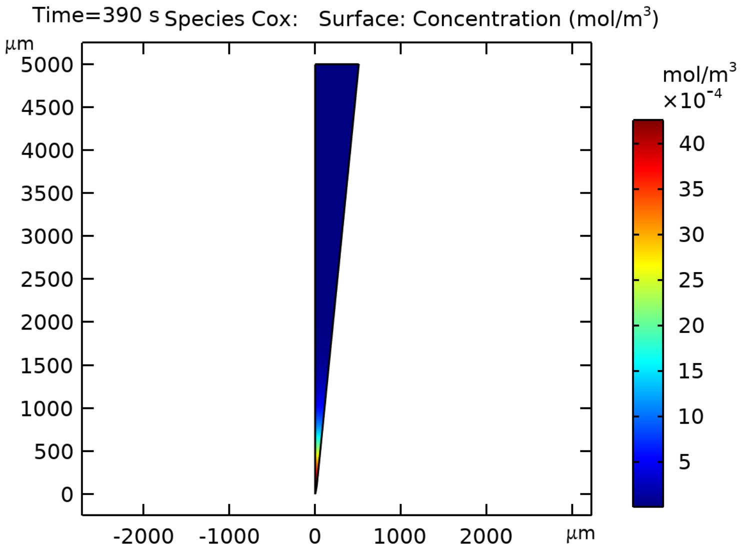

Species Cox: Surface: Concentration (mol/m^3)

4.4.2 Concentration, Cox, 3D (tds)

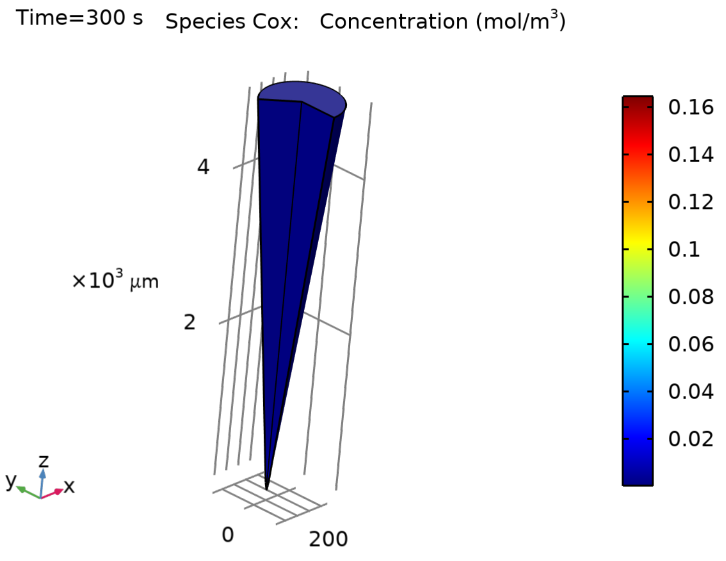

Species Cox: Concentration (mol/m^3*)*

4.4.3 Concentration, Cred (tds)

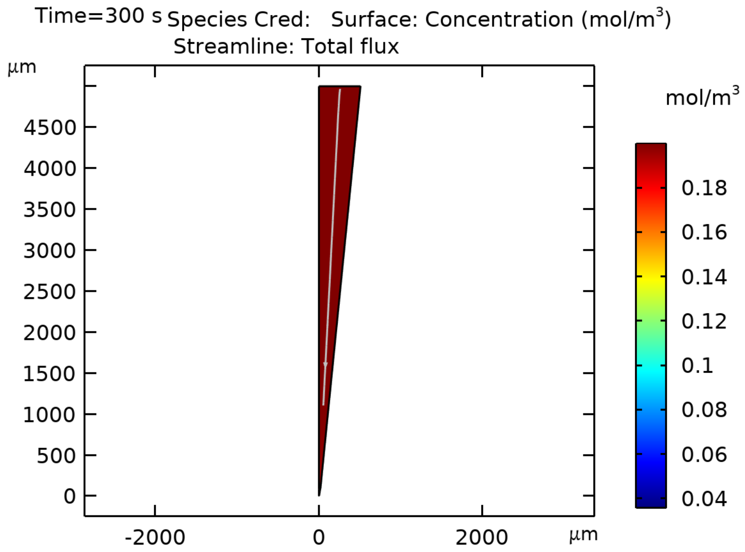

Species Cred: Surface: Concentration (mol/m^3*) Streamline: Total flux*

4.4.4 Concentration, Cred, 3D (tds)

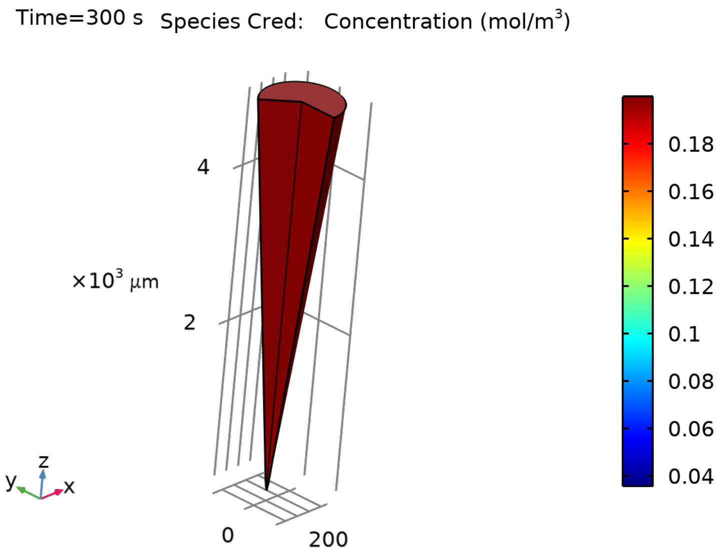

Species Cred: Concentration (mol/m³)

4.4.5 Probe Plot Group 5

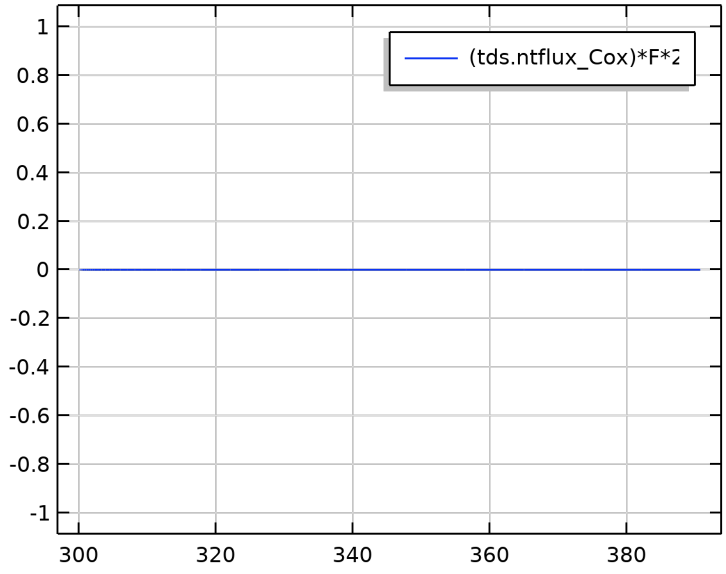

Line Graph: (mol/L)

4.4.6 1D Plot Group 6

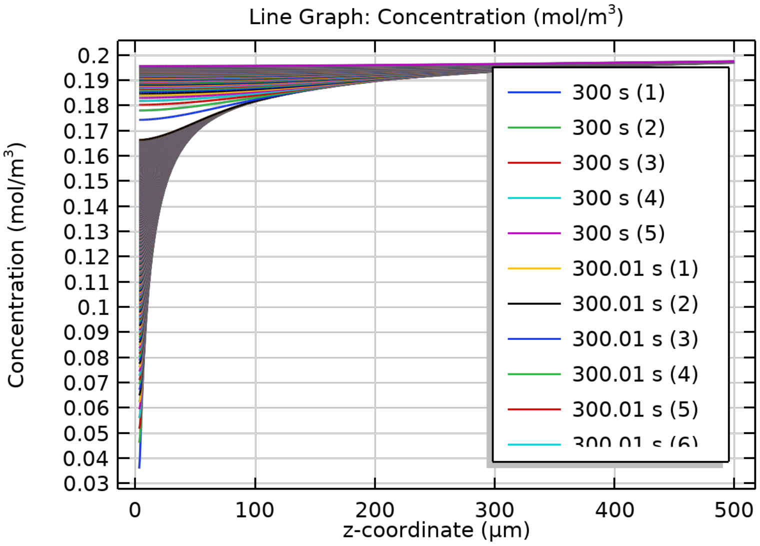

Line Graph: Concentration (mol/m³)

4.4.7 1D Plot Group 7

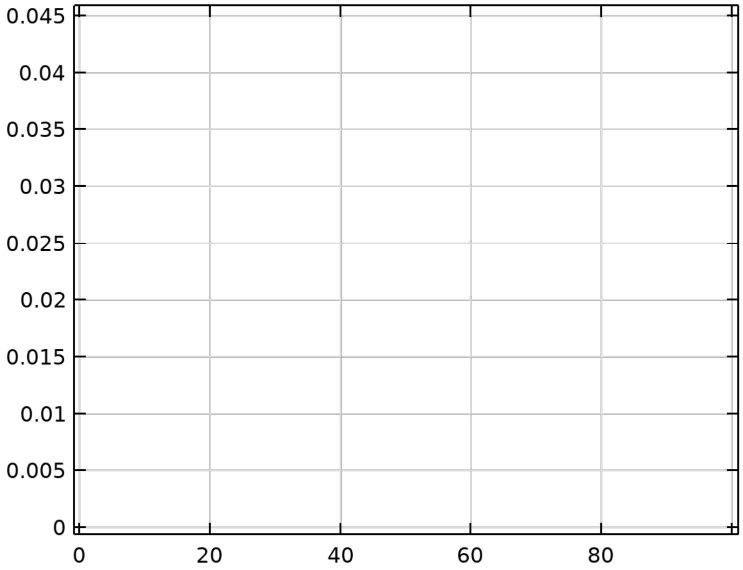

4.4.8 1D Plot Group 8

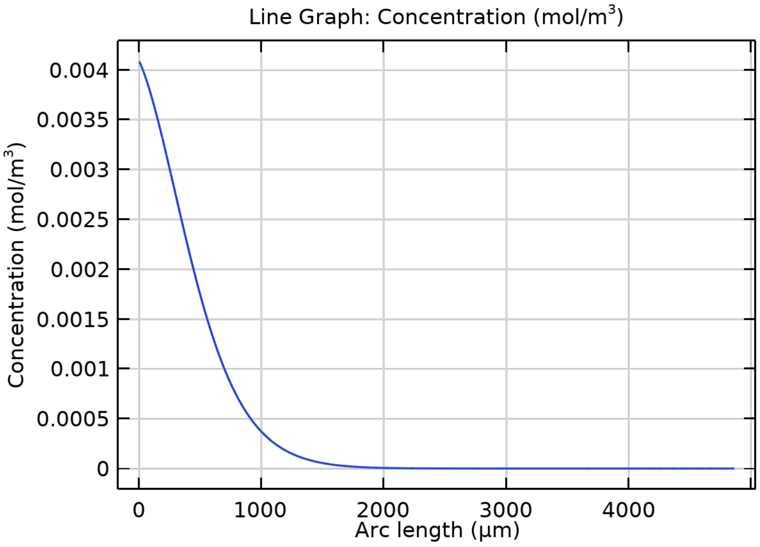

Line Graph: Concentration (mol/m^3)

4.4.9 2D Plot Group 9

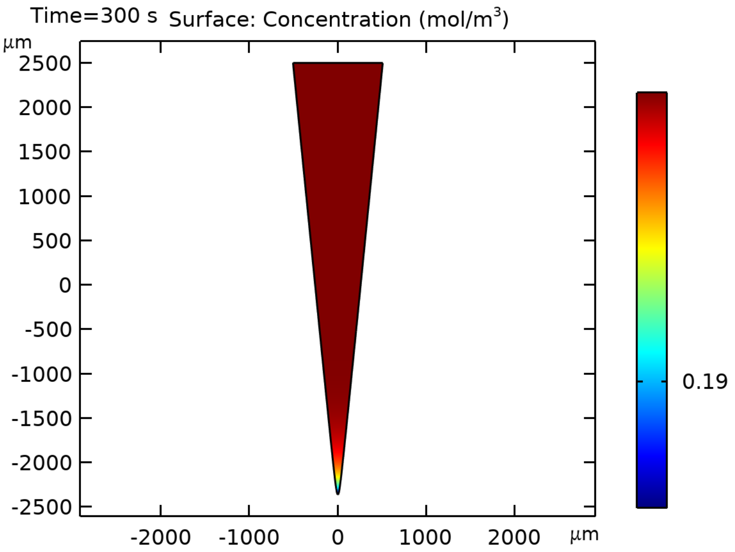

Surface: Concentration (mol/m^3)
